# Benzothiazole and Chromone Derivatives as Potential ATR Kinase Inhibitors and Anticancer Agents

**DOI:** 10.3390/molecules27144637

**Published:** 2022-07-20

**Authors:** Mykhaylo Frasinyuk, Dimple Chhabria, Victor Kartsev, Haritha Dilip, Samvel N. Sirakanyan, Sivapriya Kirubakaran, Anthi Petrou, Athina Geronikaki, Domenico Spinelli

**Affiliations:** 1 V.P. Kukhar Institute of Bioorganic Chemistry and Petrochemistry, National Academy of Science of Ukraine, 02094 Kiev, Ukraine; mykhaylo.frasinyuk@ukr.net; 2 Discipline of Chemistry, Indian Institute of Technology Gandhinagar, Gandhinagar 382055, India; dimple.c@iitgn.ac.in (D.C.); d_haritha@iitgn.ac.in (H.D.); 3 InterBioScreen, 119019 Chernogolovka, Russia; vkartsev@ibscreen.chg.ru; 4 Scientific Technological Center of Organic and Pharmaceutical Chemistry, National Academy of Science of the Republic of Armenia, Institute of Fine Organic Chemistry, Yerevan 0014, Armenia; shnnr@mail.ru; 5 School of Pharmacy, Aristotle University of Thessaloniki, 54124 Thessaloniki, Greece; anthi.petrou.thessaloniki1@gmail.com; 6 Dipartimento di Chimica “G. Ciamician”, Alma Mater Studiorum-Università di Bologna, 40126 Bologna, Italy

**Keywords:** benzothiazole, chromone, kinase inhibitor, anticancer

## Abstract

Despite extensive studies and the great variety of existing anticancer agents, cancer treatment remains an aggravating and challenging problem. Therefore, the development of novel anticancer drugs with a better therapeutic profile and fewer side effects to combat this persistent disease is still necessary. In this study, we report a novel series of benzothiazole and chromone derivatives that were synthesized and evaluated for their anticancer activity as an inhibitor of ATR kinase, a master regulator of the DDR pathway. The cell viability of a set of 25 compounds was performed using MTT assay in HCT116 and HeLa cell lines, involving 72 h incubation of the compounds at a final concentration of 10 µM. Cells incubated with compounds **2c**, **7h** and **7l** were found to show viability ≤50%, and were taken forward for dose–response studies. Among the tested compounds, three of them (**2c**, **7h** and **7l**) showed higher potency, with compound **7l** exhibiting the best IC_50_ values in both the cell lines. Compounds **2c** and **7l** were found to be equally cytotoxic towards both the cell lines, namely, HCT116 and HeLa, while compound **7h** showed better cytotoxicity towards HeLa cell line. For these three compounds, an immunoblot assay was carried out in order to analyze the inhibition of phosphorylation of Chk1 at Ser 317 in HeLa and HCT116 cells. Compound **7h** showed inhibition of pChk1 at Ser 317 in HeLa cells at a concentration of 3.995 µM. Further analysis for Chk1 and pChk1 expression was carried out in Hela cells by treatment against all the three compounds at a range of concentrations of 2, 5 and 10 µM, wherein compound **7h** showed Chk1 inhibition at 2 and 5 µM, while pChk1 expression was observed for compound **7l** at a concentration of 5 µM. To support the results, the binding interactions of the compounds with the ATR kinase domain was studied through molecular docking, wherein compounds **2c**, **7h** and **7l** showed binding interactions similar to those of Torin2, a known mTOR/ATR inhibitor. Further studies on this set of molecules is in progress for their specificity towards the ATR pathway.

## 1. Introduction

Cancer remains one of the main leading causes of mortality all over the world. Despite extensive studies and the great variety of existing anticancer agents, cancer treatment remains an aggravating and challenging problem. Therefore, the development of novel anticancer drugs with a better therapeutic profile and fewer side effects to combat this persistent disease is still necessary.

Cells are prone to a multitude of stress factors including endogenous and exogenous factors, such as reactive oxygen species, radiation, etc., which result in genomic instability through either a single strand or a double strand break in their DNA [1]. In order to cope with the stress, and to maintain genomic integrity as well as cell viability, cells develop DNA damage response (DDR) signaling pathways which regulate and repair the damage caused [2,3]. Targeting such crucial signaling pathways in cancer cells can prevent these cells from repairing their damaged single and/or double stranded DNA, hence promoting an efficient therapeutic strategy for cancer. Rad-3 related (ATR) and ataxia telangiectasia mutated (ATM) kinases from the phosphatidylinositol-3-kinase-related kinases (PIKK) family are two major signaling proteins which phosphorylate Ser/Thr residues of their downstream substrates Chk1 and CHk2, respectively [2,3,4]. The significant role of benzothiazoles as potential kinase inhibitors generates interest in exploring the biological activity of these compounds further in the area of kinases and thus, as potential candidates for cancer therapy [5].

**Figure 1:**
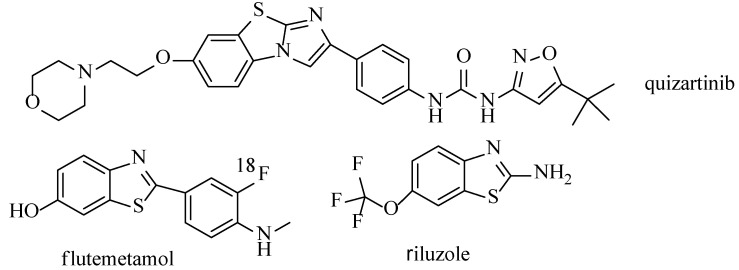
FDA-approved drugs.

Benzothiazole is a heterocyclic organic compound with a wide range of biological activities such as antimicrobial [6,7], anti-inflammatory [8,9], antioxidant [10,11], analgesic [8,12], anticancer [13,14,15,16], antiviral [17,18,19,20], anti-HIV [21,22], antidiabetic [23], anticonvulsant [24,25], antimalarial [26], antitubercular [27,28] and kinase inhibitor [29,30]. It should be mentioned that some FDA-approved drugs involve a benzothiazole carrier scaffold (Figure 1). They include quizartinib, a receptor tyrosine kinase inhibitor, flutemetamol, a diagnostic tool for Alzheimer disease, and riluzole, a drug for treatment of amyotrophic lateral sclerosis.

**Figure 2:**
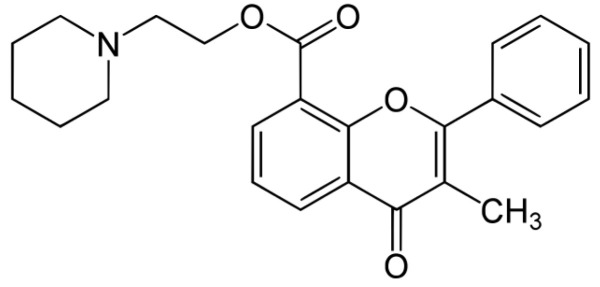
Flavoxate, FDA-approved drug.

On the other hand, chromone derivatives possess many different biological activities such as anticancer [31,32,33], antimicrobial [34], anti-inflammatory [35,36], antitubercular [37,38] and others activities [39,40,41], which have generated an interest in developing further this scaffold. The chromone scaffold is also present in some approved drugs such as flavoxate (Figure 2), a muscarinic antagonist and spasmolytic. This drug is used to treat urinary bladder spasms and is indicated for the symptomatic relief of conditions associated with lack of muscle control in the bladder, such as dysuria, urgency, and nocturia.

Taking into account these facts, we designed and synthesized new derivatives incorporating two pharmacophores, benzothiazole and chromone, in the frame of one molecule using the hybridization approach [42]. Hence, in the present study, the synthesized hybrid molecules have been analyzed for their inhibitory potential against cancer cell viability, especially on the colon cancer and cervical cancer cell lines. Further, to identify their potential in inhibiting the DDR signaling pathway, the compounds have also been subjected to assays that specifically inhibit ATR expression in these cancer cells.

## 2. Results and Discussion

### 2.1. Chemistry

The general routes for the synthesis of the target 3-hetarylchromones include the synthesis of α-[benzo]thiazolyl acetophenones **1**, ring-closure reaction for synthesis of 3-(2[benzo])thiazolyl chromones **2**–**4**, modification of the substituent in position 2 of the chromone ring, and aminomethylation of 7-hydroxychromones. The starting ketones **1a**–**1f** were synthesized by Hoesch reaction of (un)substituted resorcinol with heteroaryl acetonitriles followed by subsequent acidic hydrolysis of intermediate ketone imines [43,44,45].

Subsequent reaction of ketones **1** with ethyl orthoformate, acetic anhydride or ethyl oxalyl chloride in pyridine afforded 3-heteroaryl chromones **2**–**4**.

Thus, refluxing of ketones **1a**–**1c** and **1e** with ethyl orthoformate in dry pyridine led to the formation of 2-unsubstituted chromones **2a**–**2c** and **2e**. Ring-closure reaction of ketones **1a** or **1c** with excess of acetic anhydride in dry pyridine with subsequent saponification led to 2-methylchromones **3a** and **3c**. Reaction of ketones **1a,b,d** with ethyl oxalyl chloride in dry pyridine at room temperature afforded 2-ethoxycarbonyl-3-benzotiazolylchromones **4a**, **4b**, and **4c**. Saponification of ester **4c** in EtOH gave carboxylic acid **4d**.

**Scheme 1:**
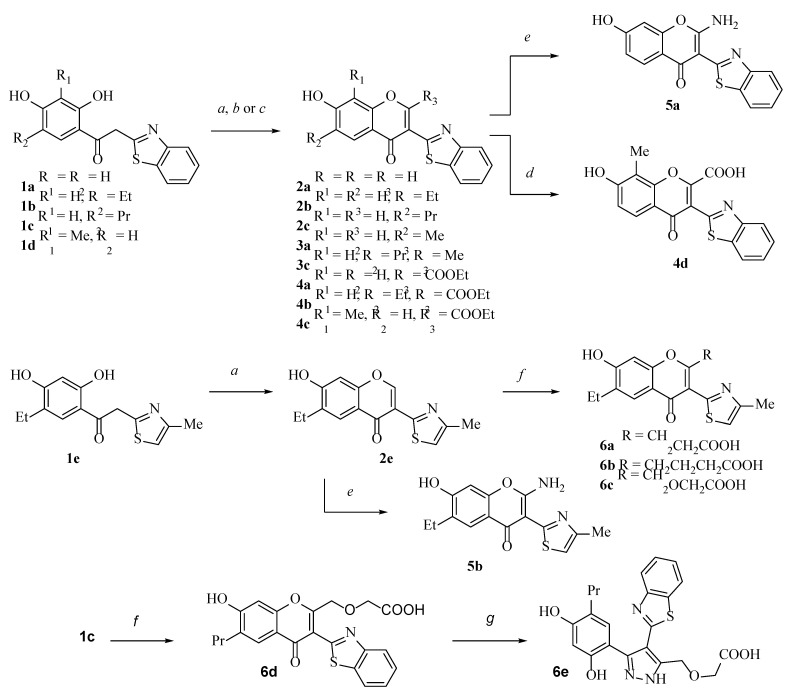
Synthesis of the key intermediates 3-hetaryl chromones **2**–**5** and target compounds (**2c, 4a,b**, and **5a**). Reagents and conditions: (a) HC(OEt)_3_, pyridine, 120 °C, 4–6 h; (b) *i* (CH_3_CO)_2_O, pyridine, r.t. 24–48 h; *ii* NaOH, EtOH, reflux, 1 min; (c) EtOOCCOCl, pyridine, r.t., 24–48 h; (d) LiOH, THF, r. t. 72 h; (e) NH_2_OH⋅HCl, pyridine, reflux, 6–8 h; (f) succinic, glutaric or diglycolic anhydride, pyridine, r.t. 72 h; (g) hydrazine, EtOH, reflux, 4 h.

The reaction of 2-unsubstituted chromones **2a** or **2e [46]** with hydroxylamine in pyridine afforded 2-aminochromones **5a** or **5b** as a result of subsequent ring-opening and ring-closure reactions. This unusual behavior of chromones **2a** or **2e** toward hydroxylamine can be explained by the strong electron-withdrawing effect of the aza-heterocyclic moiety in position 3 of the chromone ring. The bearing of ω-carboxyalkyl group 3-hetarylchromones **6a**–**6d** and related 3-aryl-4-(2-benzothiazolyl)pyrazole **6e** were synthesized as we have earlier reported [47] (Scheme 1).

**Scheme 2:**
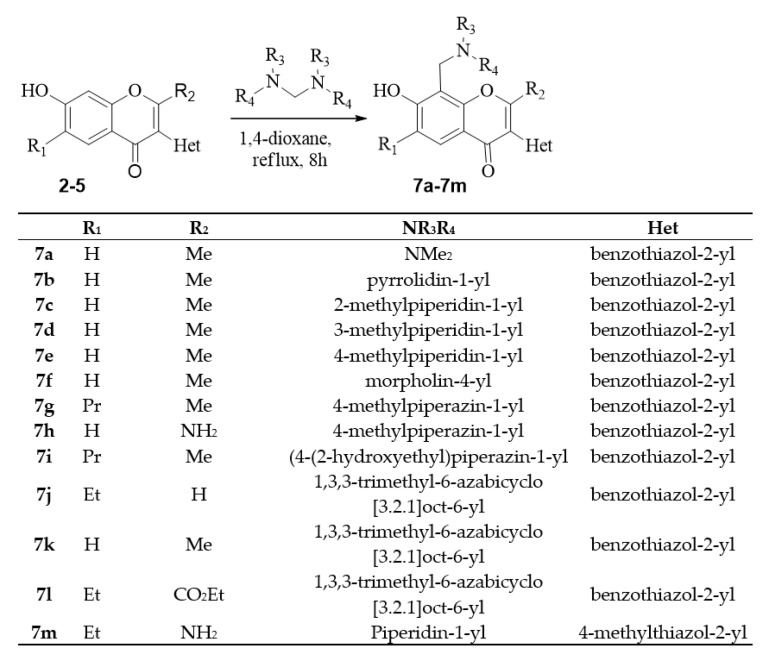
Synthesis of compounds **7a**–**7m**.

The aminomethylation of chromones with electron-withdrawing substituent in position 3 is possible by applying corresponding aminals [48]. Thus, the reaction of chromones **2**–**5** with various aminals led to regiospecific formation of 8-aminomethylchromones **7a**–**7m** (Scheme 2) which was confirmed by the disappearance in ^1^H-NMR spectra of the upfield (for 6-alkyl substituted chromones **2**–**4**) peak or presence of H-6 doublet at 6.84–6.98 ppm with ^3^*J* 8.3–8.8 Hz. The best yields were achieved using 1,4-dioxane as solvent.

Thus, in chromone Mannich bases **7** the singlet peak (except for compounds **7c**, **7j**–**7l**) of methylene group at 4.02–4.20 ppm was present as well as the secondary amine moiety. In the instance of piperidine and piperazine derivatives, in ^1^H-NMR spectra signals of cyclic amine protons usually appear as wide non-resolved multiplets. In the case of chromones **7j**–**7l** with 1,3,3-trimethyl-6-azabicyclo [3.2.1]octane moiety, we observed two doublets of diastereotopic 8-CH_2_ group at 4.00–4.05 ppm and 4.28–4.32 ppm with heminal *J* 14.3–14.5 Hz. In ^1^H-NMR peaks of 8-CH_2_ group, we observed two multiplets at 3.90 and 4.33 ppm due to difficult conversion of the piperidine ring and/or the presence of stereocenter.

**Scheme 3:**
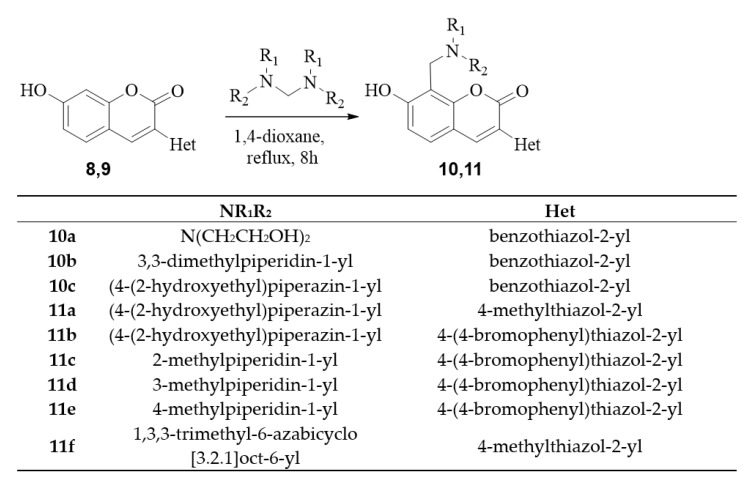
Synthesis of coumarin Mannich bases **10**–**11**.

Isomeric Mannich bases of 7-hydroxy-3-arylcoumarins were synthesized in similar conditions applying 3-(2-benzothiazolyl)-7-hydroxycoumarin (**8**) [49] and substituted 3-(2-thiazolyl)coumarins **9a,b [50]** (Scheme 3).

The synthesis of 8-aminomethyl derivatives was confirmed by splitting of H-5 and H-6 of coumarin core with ^3^*J* 8.6–8.7 Hz. Similar to chromone **7** derivatives, the signal of 8-CH_2_ group was found as a singlet at 3.99–4.23 ppm for diethanolamine, piperidine or piperazine derivatives. In the case of compound **11f**, the signals of 8-CH_2_ group were found as two doublets at 4.07 and 4.37 ppm with ^2^*J* 14.6 Hz.

### 2.2. Biological Evaluation

#### 2.2.1. Cell Viability Studies—Initial Screening

**Figure 3:**
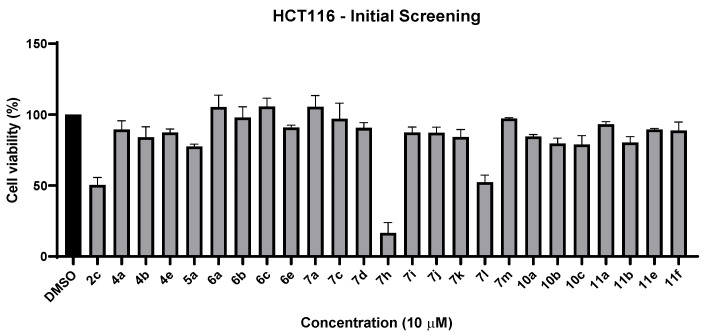
Initial screening results for HCT116 cells after 72 h incubation.

In order to determine the effect of the compounds on cell viability, the studies were carried out using MTT assay in HCT116 and HeLa cell lines. Initial screening of the compounds was carried out at a final concentration of 10 µM. The cell viability was reduced by ≤50% in the presence of three compounds, namely **2c**, **7h** and **7l**, hence showing cell toxicity in both cell lines at a final concentration of 10 µM. Figure 3 represents the initial screening results for HCT116 cells after 72 h incubation.

**Figure 4:**
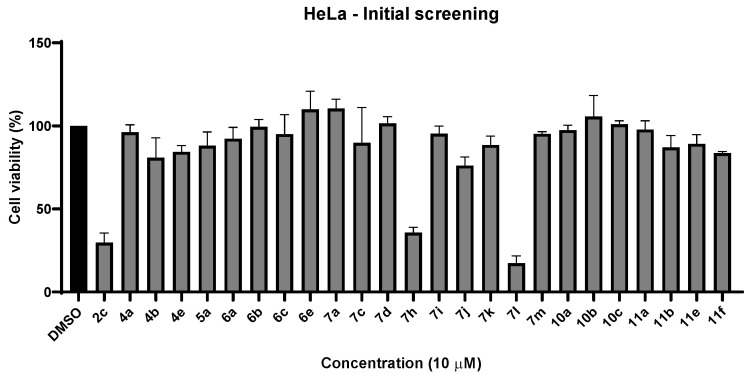
Initial screening results for HeLa cells after 72 h incubation.

Similarly, Figure 4 represents the initial screening results for HeLa cells after 72 h incubation.

Compounds **2c**, **7h** and **7l** showed a significant reduction in cell viability for both the cervical cancer and colon cancer cell lines, suggesting that these compounds could be potent anticancer agents, especially for these two types of cancer. In order to identify the minimum concentration of these compounds required to reduce the cell viability of these cell lines by 50% or more, these compounds were further analyzed for their cytotoxicity effects using dose-response studies. The dose-response studies would be performed under similar conditions as those for the initial screening, for a concentration range of the potent compounds, so as to identify the half maximal inhibitory concentration (IC_50_) of each compound required to cause cell toxicity.

#### 2.2.2. Cell Viability Studies—Dose Response

The dose–response studies were conducted using the MTT assay for **2c**, **7h** and **7l**, for a dose range of 0–100 µM similar to the initial screening protocol. Further, the absorbance was measured at 570 nm using the PerkinElmer EnVision Multilabel Reader and the IC_50_ values calculated for all the three compounds (Table 1).

**Figure 5:**
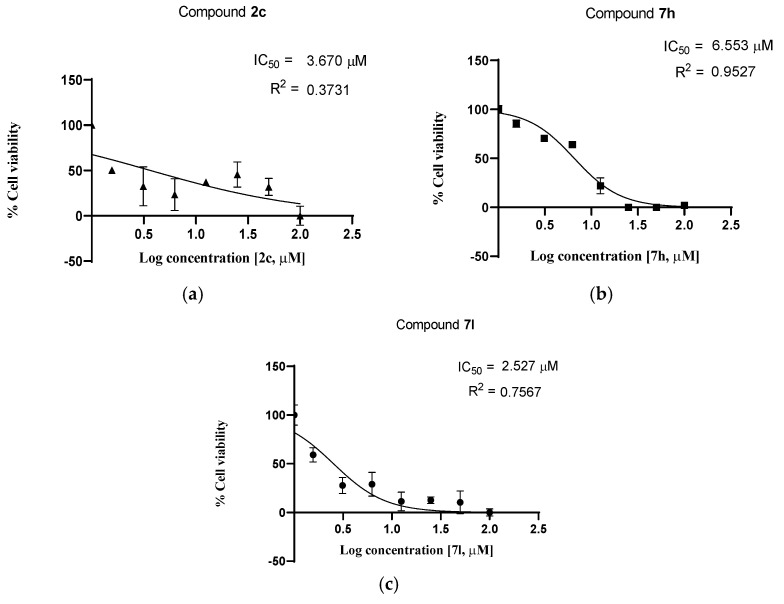
Dose response curves for the compounds in HCT116 cells after 72 h incubation.

Figure 5 represents the dose–response curves for the compounds **2c**, **7h** and **7l** in HCT116 cells after 72 h incubation.

**Figure 6:**
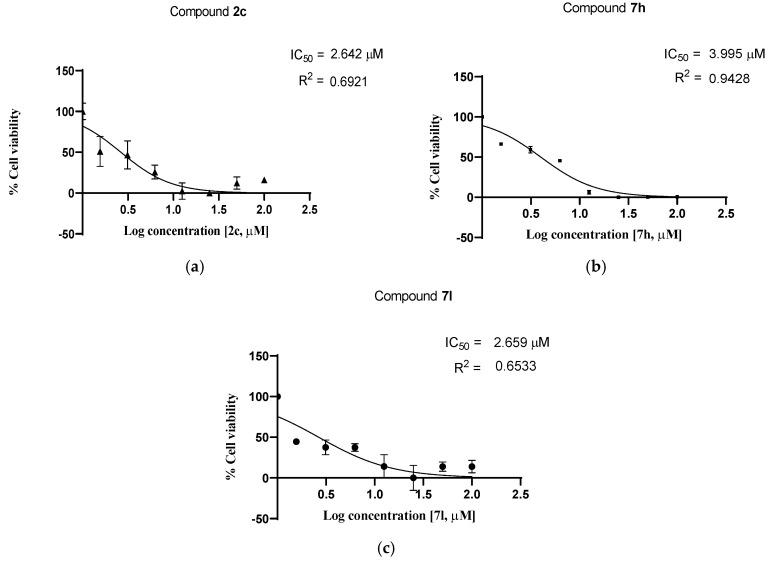
Dose–response curves for the compounds in HeLa cells after 72 h incubation.

Similarly, Figure 6 represents the dose–response curves for the compounds **2c**, **7h** and **7l** in HCT116 cells after 72 h incubation.

Compounds **2c** and **7h** showed IC_50_ values of 3.670 µM and 6.553 µM against HCT116 cell lines, while **7l** showed an IC_50_ value of 2.527 µM. However, all three compounds were equally potent against the HeLa cell line with IC_50_ values of 2.642 µM, 3.995 µM and 2.659 µM, respectively. Further, to analyze the effect of these compounds specifically on the DDR signaling pathway, an essential pathway for cancer cell survival, the compounds were subjected to cell-based assays that would determine the inhibition of these pathways.

#### 2.2.3. Immunoblot Assay

The effect of the synthesized compounds to inhibit the cancer cell’s DNA damage response (DDR) signalling pathway, a common pathway preferred by cancer cells to ensure genomic integrity, as well as cell viability [51], was analyzed by carrying out an immunoblot assay for one of the major kinases involved in the regulation of the pathway. Ataxia telangiectasia mutated and Rad-3 related (ATR) kinase, a member of the the PIKK (phosphatidylinositol-3-kinase-related kinases) family, are involved in the phosphorylation of Ser/Thr residues of its downstream substrate Chk1. The inhibition of Chk1 phosphorylation has been proved to be a clear indication of the inhibition of ATR signalling. Hence, the immunoblot assay helps in the determination of the inhibitory potential of the synthesized compounds against ATR kinase in the DDR pathway.

**Figure 7:**
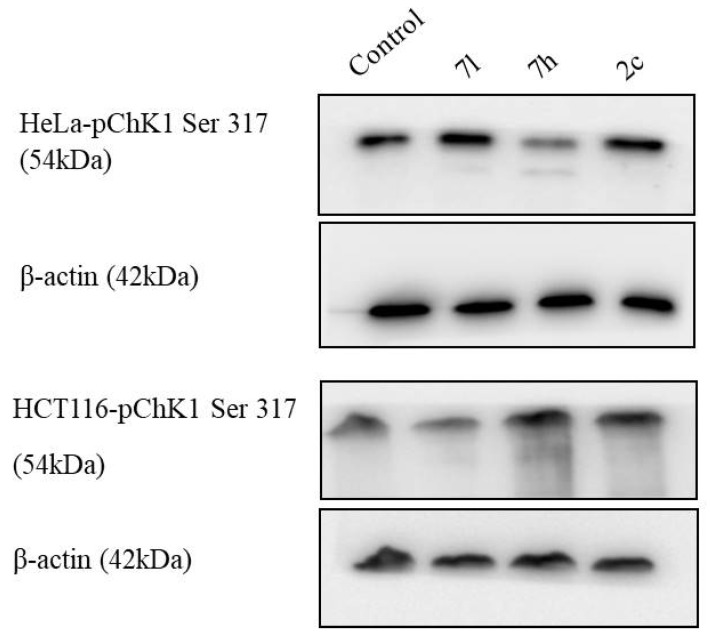
Inhibition of Chk1 at Ser 317 in HeLa and HCT116 cells analyzed by immunoblot assay.

The analysis of inhibition of phosphorylation of Chk1 at Ser 317 in HeLa and HCT116 cells was carried out by immunoblotting using anti-pChk1 Ser 317 (rabbit) primary antibody and anti-rabbit IgG HRP-linked secondary antibody. Of the three compounds tested, compound **7h** showed inhibition of ATR signaling which was observed by checking the status of ChK1 in HeLa cells (Figure 7). However, the results for HCT116 do not show significant inhibition of ATR signaling in the cases of compounds **7h** and **2c**, while **7l** showed inhibition with respect to the control.

**Figure 8:**
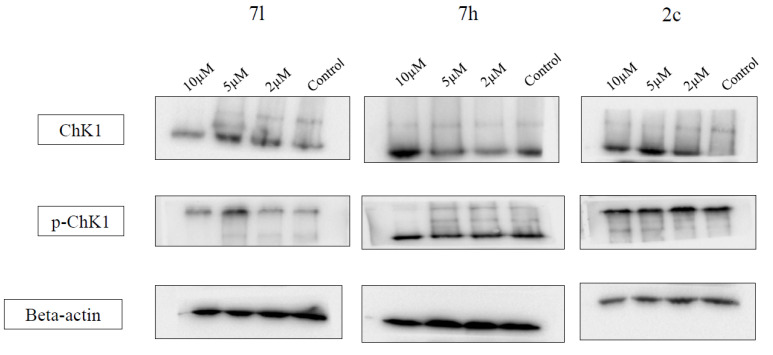
Expression of Chk1 and pChk1 at Ser 317 in cells analyzed by immunoblot assay with respect to treatment against compounds **7l**, **7h** and **2c**.

Further, to analyze the effect of the concentration of the compounds on the expression of Chk1 and pChk1 at Ser 317 in HeLa cells, immunoblotting assay using ChK1 mouse monoclonal antibody, and anti-pChk1 Ser 317 (rabbit) primary antibody were carried out. Anti-mouse IgG HRP-linked antibody and anti-rabbit IgG HRP-linked secondary antibody were used as secondary antibodies, respectively. The inhibition of Chk1 was observed at 2 µM and 5 µM concentrations of compound **7h**. In the cases of compounds **2c** and **7l**, the ChK1 and pChK1 expression in the control lanes did not provide a clear insight into the inhibition of ATR signaling (Figure 8).

### 2.3. Molecular Docking

The use of molecular docking as a successful computational tool in drug discovery, to model and visualize the interactions between small molecules and their specific targets, has been prevalent since the 1980s. The conformations and binding interactions corresponding to the behavior of the small molecules at the binding site of the drug target have also been an essential factor in identifying the mechanism of enzyme inhibition, which further enhance the drug development processes [52].

Molecular docking resulted in an in-depth analysis of the major binding interactions between the compounds with the ATR kinase domain. Compound **2c** showed the same GScore and DockScore of −5.5 kcal/mol, followed by a GScore and DockScore of −5.2 kcal/mol and −5.1 kcal/mol for Torin2. Compound **7l** showed the GScore and DockScore of −5.2 kcal/mol and −4.8 kcal/mol. This was followed by compound **7h** with a GScore and DockScore of −4.3 kcal/mol and −3.4 kcal/mol (Table 2).

**Figure 9:**
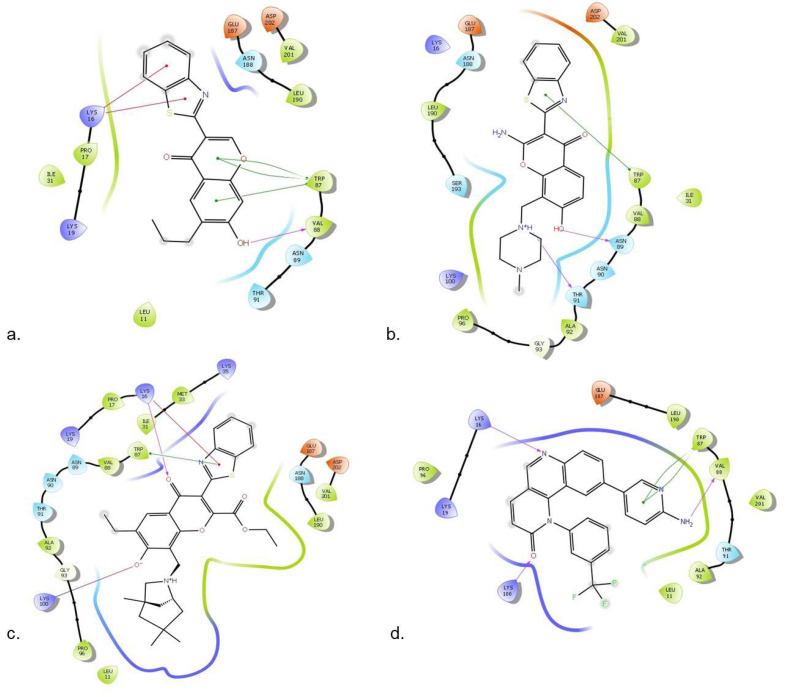
Ligand interaction diagrams for the compounds (**a**) **2c**, (**b**) **7h**, (**c**) **7l** and (**d**) Torin2.

Two-dimensional ligand interaction diagrams show the major binding interactions between the compounds and the binding site of the ATR kinase domain. Torin2, the standard ligand had hydrogen bonding and π-π stacking interactions as the major interactions at the binding site. Compound **2c** had the highest docking score of −5.5 kcal/mol, wherein hydrogen bonding, π-π stacking and π-cation interactions played the major role. Compound **7h** had similar interactions in comparison to Torin2, with a lowest docking score of −3.4 kcal/mol. However, unlike all the ligands in the molecular docking study, compound **7l** had the highest number of diverse interactions at the binding site, including hydrogen bonding, π-π stacking, π-cation interactions and salt bridge formation. The ligand interaction diagrams for the binding interactions of compounds **2c**, **7h** and **7l** along with Torin2 are shown in Figure 9.

**Figure 10:**
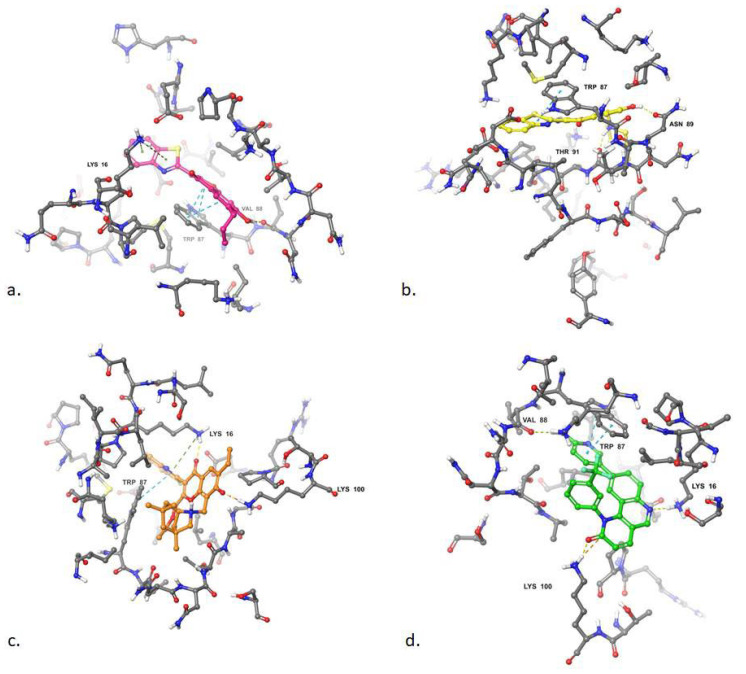
Binding pose for the compounds (**a**) 2c, (**b**) 7h, (**c**) 7l and (**d**) Torin2 at the binding site.

The three-dimensional view of the ligand conformations in the binding site is crucial to visualize the interactions determined through ligand interaction diagrams. The binding poses of the compounds, in comparison to the standard ligand helps in deciphering the nature of binding of these compounds at the binding site. The 3D diagrams indicating the binding poses of the compounds **2c** (pink), **7h** (yellow), **7l** (orange) and Torin2 (green) are depicted in Figure 10.

## 3. Materials and Methods

### 3.1. General Information

^1^H and ^13^C spectra were recorded on Varian 500 (500/125 MHz) or Varian 400 (400/100 MHz) and/or Varian VXR300 spectrometers in CDCl_3_ [residual CHCl_3_ (δ_H_ = 7.26 ppm) or CDCl_3_ (δ_C_ = 77.16 ppm) as internal standard] or DMSO-*d*_6_ [residual SO(CD_3_)(CD_2_H) (δ_H_ = 2.50 ppm) or SO(CD_3_)_2_ (δ_C_ = 39.52 ppm) as internal standard]. Melting points were determined in open capillary tubes using Buchi B-535 apparatus (Buchi Labortechnik AG, Flawil, Switzerland) and were uncorrected. Mass spectra were obtained using an Agilent 1100 spectrometer (Agilent Technologies, Waldbronn, Germany) using APCI (atmospheric-pressure chemical ionization). All spectra are available in Supplementary Materials.

Colon cancer cell line HCT116 and human cervical cancer cell line HeLa were furnished by National Centre for Cell Science (NCCS), Pune, India, DMEM from LONZA, Fetal bovine serum, pen-strap, and trypsin-EDTA from Invitrogen corporation (Carlsbad, CA, USA), MTT [3-(4,5-Dimethyl-2-thiazolyl)-2,5-diphenyl tetrazolium bromide] dye from HIMEDIA (Mumbai, India), and transparent 96-well plates from TARSON (Tarsons Products Pvt. Ltd., Mumbai, India).

Sodium dodecyl sulphate (SDS), tetramethylenediamine (TEMED), ammonium persulphate (APS), bromophenol blue and beta-mercaptoethanol were purchased from HIMEDIA (Mumbai, India), Tween-20 and Bovine serum albumin (BSA) from Sigma-Aldrich (Darmstadt, Germany), complete-EDTA free protease inhibitor tablets from Roche (Basel, Switzerland), immunoblot PVDF Western blotting membrane and Clarity ECL Western blotting substrate from Bio-Rad laboratories (Hercules, CA, USA), ChK1- phosphor Ser 317 (Cat. 12302S), ChK1 Mouse (Cat. 2360S) monoclonal antibody, Anti-mouse IgG HRP-linked antibody (Cat. 7076P2) and Anti-rabbit IgG HRP- linked secondary antibody (Cat. 7074) from Cell Signaling Technology (Danvers, MA, USA), and mouse anti-human Beta-actin (Cat. SC47778) from Santa Cruz Biotechnology (Dallas, TX, USA).

### 3.2. Chemistry

Compounds **1a** [43], **1b** [45], **1c** [44], **1e** [46], **2a** [43], **2b** [45], **2c** [44], **2e** [46], **3c** [44], **6a**–**6d** [47], **8** [48], and **9a,b** [49] were synthesized by procedures reported earlier.

*2-(1,3-Benzothiazol-2-yl)-1-(2,4-dihydroxy-3-methylphenyl)ethanone* (**1d**) was synthesized according to the literature procedure [44]. Yield 53%, m.p. 255–256 °C. LC-MS: *m*/*z* 300.0 [M + H]^+^. ^1^H-NMR (400 MHz, DMSO-d_6_): δ ppm 14.52 (s, 1H), 12.65 (s, 1H), 9.96 (s, 1H), 7.76 (d, *J* = 7.7 Hz, 1H), 7.48 (d, *J* = 8.7 Hz, 1H), 7.39–7.30 (m, 2H), 7.21–7.11 (m, 1H), 6.69 (s, 1H), 6.43 (d, *J* = 8.6 Hz, 1H), 2.01 (s, 3H). ^13^C-NMR (125 MHz, DMSO-*d*_6_): δ ppm 186.0, 162.4, 161.9, 159.9, 138.8, 126.8, 126.7, 125.7, 122.5, 122.3, 112.1, 111.6, 110.4, 106.3, 85.9, 7.9. Elemental analysis for C_16_H_13_NO_3_S; Calc.: C, 64.20; H, 4.38; N, 4.68%. Found: C, 64.22; H, 4.35; N, 4.70%.

*3-(1,3-Benzothiazol-2-yl)-7-hydroxy-2-methyl-4H-chromen-4-one* (**3a**) was synthesized according to the literature procedure [45]. Yield 78%, m.p. 292–293 °C. LC-MS: *m*/*z* 310.1 [M + H]^+^. ^1^H-NMR (400 MHz, DMSO-*d*_6_): δ ppm 10.94 (s, 1H), 8.08 (d, *J* = 7.9 Hz, 1H), 8.04–7.93 (m, 2H), 7.53–7.45 (m, 1H), 7.44–7.37 (m, 1H), 6.95 (d, *J* = 8.7 Hz, 1H), 6.85 (s, 1H), 2.95 (s, 3H). ^13^C-NMR (125 MHz, DMSO-*d*_6_): δ ppm 173.7, 168.9, 163.1, 159.9, 156.4, 150.9, 135.0, 127.1, 125.8, 124.8, 122.3, 121.4, 115.6, 114.5, 114.4, 102.0, 21.7. Elemental analysis for C_17_H_13_NO_3_S; Calc.: C, 66.01; H, 3.58; N, 4.53%. Found: C, 66.05; H, 3.60; N, 4.56%.

#### 3.2.1. General Procedure for the Synthesis of 2-Ethoxycarbonyl Chromones **4a**–**4c**

To a solution of the corresponding starting compounds **1a,b,d** (5 mmol) in dry pyridine (10 mL) ethyl oxalyl chloride (15 mmol) was added dropwise under cooling. The reaction mixture was stirred for 24–48 h at room temperature and poured into 200 mL of 1N HCl. The formed precipitate was filtered off, dried, and re-crystallized from EtOH to furnish the desired products **4a**–**4c**.

*Ethyl 3-(1,3-benzothiazol-2-yl)-7-hydroxy-4-oxo-4H-chromene-2-carboxylate* (**4a**). Yield 67%, m.p. 223–224 °C, yellow crystals. LC-MS: *m*/*z* 368.0 [M + H]^+^. ^1^H-NMR (400 MHz, DMSO-*d*_6_): δ ppm 11.23 (s, 1H), 8.17 (d, *J* = 7.9 Hz, 1H), 8.07 (d, *J* = 8.7 Hz, 1H), 7.98 (d, *J* = 8.1 Hz, 1H), 7.57–7.51 (m, 1H), 7.46 (t, *J* = 7.4 Hz, 1H), 7.06 (d, *J* = 8.8 Hz, 1H), 7.01 (s, 1H), 4.45 (q, *J* = 7.0 Hz, 2H), 1.24 (t, *J* = 7.0 Hz, 3H). ^13^C-NMR (125 MHz, DMSO-*d*_6_): δ ppm 173.5, 164.1, 161.1, 156.8, 156.6, 154.5, 150.8, 135.6, 127.4, 126.5, 125.4, 122.4, 122.1, 116.7, 115.1, 113.8, 102.5, 63.0, 13.6. Elemental analysis for C_19_H_13_NO_5_S; Calc.: C, 62.12; H, 3.57; N, 3.81%. Found: C, 62.09; H, 3.60; N, 3.79%.

*Ethyl 3-(1,3-benzothiazol-2-yl)-6-ethyl-7-hydroxy-4-oxo-4H-chromene-2-carboxylate* (**4b**). Yield 74%, m.p. 192–193 °C, colorless. LC-MS: *m*/*z* 396.0 [M + H]^+^. ^1^H-NMR (500 MHz, DMSO-*d*_6_): δ ppm 11.30 (s, 1H), 8.19 (d, *J* = 7.9 Hz, 1H), 7.98 (d, *J* = 8.1 Hz, 1H), 7.92 (s, 1H), 7.59–7.52 (m, 1H), 7.50–7.44 (m, 1H), 7.01 (s, 1H), 4.45 (q, *J* = 7.1 Hz, 2H), 2.67 (q, *J* = 7.4 Hz, 2H), 1.23 (t, *J* = 7.1 Hz, 3H), 1.21 (t, *J* = 7.4 Hz, 3H). ^13^C-NMR (125 MHz, DMSO-*d*_6_): δ ppm 173.3, 162.1, 161.2, 156.9, 154.9, 154.3, 150.8, 135.5, 131.9, 126.5, 125.4, 124.9, 122.4, 122.1, 114.8, 113.7, 101.7, 62.9, 22.4, 13.6, 13.5. Elemental analysis for C_21_H_17_NO_5_S; Calc.: C, 63.79; H, 4.53; N, 3.54%. Found: C, 63.77; H, 4.49; N, 3.57%.

*Ethyl 3-(1,3-benzothiazol-2-yl)-7-hydroxy-8-methyl-4-oxo-4H-chromene-2-carboxylate* (**4c**). Yield 69%, m.p. 265–266 °C. LC-MS: *m*/*z* 382.0 [M + H]^+^. ^1^H-NMR (400 MHz, DMSO-*d*_6_): δ ppm 11.07 (s, 1H), 8.16 (d, *J* = 7.7 Hz, 1H), 7.98 (d, *J* = 8.0 Hz, 1H), 7.93 (d, *J* = 8.7 Hz, 1H), 7.58–7.50 (m, 1H), 7.49–7.41 (m, 1H), 7.11 (d, *J* = 8.7 Hz, 1H), 4.47 (q, *J* = 7.0 Hz, 2H), 2.24 (s, 3H), 1.23 (t, *J* = 7.1 Hz, 3H). ^13^C-NMR (125 MHz, DMSO-*d*_6_): δ ppm 173.9, 161.6, 161.2, 156.9, 154.5, 154.5, 150.8, 135.6, 126.4, 125.3, 124.0, 122.4, 122.0, 115.2, 115.1, 113.4, 111.5, 62.9, 13.6, 7.9. Elemental analysis for C_20_H_15_NO_5_S; Calc.: C, 62.98; H, 3.96; N, 3.67%. Found: C, 62.94; H, 3.99; N, 3.63%.

*3-(1,3-Benzothiazol-2-yl)-7-hydroxy-8-methyl-4-oxo-4H-chromene-2-carboxylic acid* (**4d**). To a stirred solution of ester 4**d** (1 mmol) in THF (10 mL), a solution of LiOH⋅2H_2_O (2 mmol) in water (2 mL) was added at room temperature. After stirring for 24 h, reaction was quenched by 1N HCl (5 mL), formed precipitate was filtered off, dried, and re-crystallized from EtOH to furnish the desired products **4e** as yellow crystals. Yield 58%, m.p. 342–343 °C. LC-MS: *m*/*z* 354.0 [M + H]^+^. ^1^H-NMR (400 MHz, DMSO-*d*_6_): δ ppm 11.16 (s, 1H), 8.16 (d, *J* = 7.8 Hz, 1H), 7.98 (d, *J* = 8.0 Hz, 1H), 7.92 (d, *J* = 8.7 Hz, 1H), 7.62–7.50 (m, 1H), 7.50–7.41 (m, 1H), 7.17 (d, *J* = 8.7 Hz, 1H), 2.26 (s, 3H). ^13^C-NMR (125 MHz, DMSO-*d*_6_): δ ppm 174.2, 162.4, 161.6, 157.5, 156.0, 154.5, 151.2, 135.7, 126.2, 125.2, 123.9, 122.5, 122.0, 115.2, 115.1, 113.0, 111.5, 7.9. Elemental analysis for C_18_H_11_NO_5_S; Calc.: C, 61.18; H, 3.14; N, 3.96%. Found: C, 61.21; H, 3.12 N, 3.98%.

#### 3.2.2. General Procedure for the Synthesis of 2-Aminochromones **5a,b**

A mixture of chromone **2a,e** (1 mmol) and hydroxylamine hydrochloride (2 mmol) in pyridine (5 mL) was refluxed for 6–8 h. The reaction mixture was cooled, and the formed precipitate was filtered off, dried, and re-crystallized from EtOH to furnish the desired products **5a,b**.

*2-Amino-3-(1,3-benzothiazol-2-yl)-7-hydroxy-4H-chromen-4-one* (**5a**). Yield 46%, m.p. 364–365, colorless. °C. LC-MS: *m*/*z* 311.0 [M + H]^+^. ^1^H-NMR (400 MHz, DMSO-*d*_6_): δ ppm 10.86 (s, 1H), 10.68 (s, 1H), 9.46 (s, 1H), 8.02 (d, *J* = 7.7 Hz, 1H), 7.98–7.90 (m, 2H), 7.49–7.40 (m, 1H), 7.36–7.28 (m, 1H), 6.89 (d, *J* = 8.6 Hz, 1H), 6.77 (s, 1H). ^13^C-NMR (125 MHz, DMSO-*d*_6_): δ ppm 172.2, 163.7, 162.8, 162.1, 153.6, 149.9, 133.0, 127.0, 125.7, 123.7, 121.3, 120.6, 114.0, 113.4, 101.6, 92.0. Elemental analysis for C_16_H_10_N_2_O_3_S; Calc.: C, 61.93; H, 3.25; N, 9.03%. Found: C, 61.91; H, 3.22; N, 9.00%.

*2-Amino-6-ethyl-7-hydroxy-3-(4-methyl-1,3-thiazol-2-yl)-4H-chromen-4-one* (**5b**). Yield 74%, m.p. 290–291 °C. LC-MS: *m*/*z* 303.2 [M + H]^+^. ^1^H-NMR (400 MHz, DMSO-*d*_6_): δ ppm 10.57 (s, 2H), 9.00 (s, 1H), 7.76 (s, 1H), 6.96 (s, 1H), 6.78 (s, 1H), 2.60 (q, *J* = 7.3 Hz, 2H), 2.39 (s, 3H), 1.17 (t, *J* = 7.4 Hz, 3H). ^13^C-NMR (125 MHz, DMSO-*d*_6_): δ ppm 171.6, 162.9, 161.1, 159.5, 151.6, 147.9, 128.6, 124.8, 113.2, 111.2, 100.9, 92.2, 22.3, 16.8, 13.8. Elemental analysis for C_15_H_14_N_2_O_3_S; Calc.: C, 59.59; H, 4.67; N, 9.27%. Found: C, 59.57; H, 4.65; N, 9.30%.

#### 3.2.3. General Procedure for Synthesis of 8-Aminomethyl Derivatives **7**

A stirring mixture of the corresponding chromone (1 mmol) and aminal [44] (1.2 mmol) in 1,4-dioxane (5 mL) was refluxed for 6–8 h. The reaction mixture was cooled, diluted with 10 mL of hexane. The formed precipitate was filtered off, dried, and re-crystallized from iPrOH-hexanes mixture to furnish the desired chromone Mannich bases **7**.

*3-(1,3-Benzothiazol-2-yl)-8-[(dimethylamino)methyl]-7-hydroxy-2-methyl-4H-chromen-4-one* (**7a**). Yield 71%, m.p. 224–225 °C, pale pink. LC-MS: *m*/*z* 367.2 [M + H]^+^. ^1^H-NMR (500 MHz, CDCl_3_): δ ppm 8.13 (d, *J* = 8.8 Hz, 1H), 8.05 (d, *J* = 8.1 Hz, 1H), 7.98 (d, *J* = 7.7 Hz, 1H), 7.52–7.45 (m, 1H), 7.43–7.35 (m, 1H), 6.92 (d, *J* = 8.8 Hz, 1H), 4.02 (s, 2H), 3.04 (s, 3H), 2.46 (s, 6H). ^13^C-NMR (125 MHz, CDCl_3_): δ ppm 175.2, 167.9, 164.7, 160.5, 154.3, 151.8, 136.1, 126.9, 125.8, 124.9, 122.8, 121.4, 116.2, 115.7, 115.2, 107.3, 55.3, 44.7, 22.0. Elemental analysis for C_20_H_18_N_2_O_3_S; Calc.: C, 65.55; H, 4.95; N, 7.64%. Found: C, 65.57; H, 4.93; N, 7.66%.

*3-(1,3-Benzothiazol-2-yl)-7-hydroxy-2-methyl-8-(pyrrolidin-1-ylmethyl)-4H-chromen-4-one* (**7b**). Yield 67%, m.p. 214–217 °C, colorless. LC-MS: *m*/*z* 393.0 [M + H]^+^. ^1^H-NMR (300 MHz, CDCl_3_): δ ppm 11.47 (s, 1H), 8.12 (d, *J* = 8.8 Hz, 1H), 8.07–8.01 (m, 1H), 8.00–7.94 (m, 1H), 7.51–7.43 (m, 1H), 7.43–7.35 (m, 1H), 6.90 (d, *J* = 8.8 Hz, 1H), 4.19 (s, 2H), 3.03 (s, 3H), 2.91–2.66 (m, 4H), 2.03–1.83 (m, 4H). ^13^C-NMR (125 MHz, CDCl_3_): δ ppm 175.2, 167.9, 164.9, 160.6, 154.0, 151.8, 136.1, 126.7, 125.7, 124.8, 122.8, 121.4, 116.2, 115.7, 115.1, 107.8, 53.9, 51.4, 23.9, 22.0. Elemental analysis for C_22_H_20_N_2_O_3_S; Calc.: C, 67.33; H, 5.14; N, 7.14%. Found: C, 67.36; H, 5.12; N, 7.17%.

*3-(1,3-Benzothiazol-2-yl)-7-hydroxy-2-methyl-8-[(2-methylpiperidin-1-yl)methyl]-4H-chromen-4-one* (**7c**). Yield 79%, m.p. 204–205 °C, colorless. LC-MS: *m*/*z* 421.2 [M + H]^+^. ^1^H-NMR (400 MHz, CDCl_3_): δ ppm 9.83 (s, 1H), 8.08 (d, *J* = 8.8 Hz, 1H), 8.03 (d, *J* = 8.1 Hz, 1H), 7.96 (d, *J* = 7.9 Hz, 1H), 7.50–7.41 (m, 1H), 7.40–7.32 (m, 1H), 6.86 (d, *J* = 8.8 Hz, 1H), 4.48–4.23 (m, 1H), 3.90 (d, *J* = 15.0 Hz, 1H), 3.14–2.85 (m, 4H), 2.75–2.13 (m, 2H), 1.91–1.34 (m, 6H), 1.24 (d, *J* = 6.1 Hz, 3H); ^13^C-NMR (125 MHz, CDCl_3_): δ ppm 175.0, 167.9, 164.5, 160.5, 154.2, 151.7, 136.0, 126.3, 125.6, 124.7, 122.8, 121.3, 116.3, 115.5, 114.9, 107.4, 57.8, 54.5, 50.8, 35.4, 34.2, 25.6, 25.2, 21.9. Elemental analysis for C_24_H_24_N_2_O_3_S; Calc.: C, 68.55; H, 5.75; N, 6.66%. Found: C, 68.57; H, 5.73; N, 6.67%.

*3-(1,3-Benzothiazol-2-yl)-7-hydroxy-2-methyl-8-[(3-methylpiperidin-1-yl)methyl)]-4H-chromen-4-one* (**7d**). Yield 83%, m.p. 200–201 °C, colorless. LC-MS: *m*/*z* 421.0 [M + H]^+^. ^1^H-NMR (500 MHz, CDCl_3_): δ ppm 8.11 (d, *J* = 8.8 Hz, 1H), 8.03 (d, *J* = 8.1 Hz, 1H), 7.96 (d, *J* = 7.9 Hz, 1H), 7.53–7.43 (m, 2H), 7.41–7.32 (m, 1H), 6.89 (d, *J* = 8.8 Hz, 1H), 4.03 (s, 2H), 3.02 (s, 3H), 3.00–2.85 (m, 1H), 2.14–1.56 (m, 6H), 1.14–0.95 (m, 1H), 0.92 (d, *J* = 6.2 Hz, 3H). ^13^C-NMR (125 MHz, CDCl_3_): δ ppm 175.2, 167.9, 164.9, 160.5, 154.4, 151.8, 136.1, 126.7, 125.7, 124.8, 122.8, 121.4, 116.2, 115.7, 115.1, 107.0, 61.1, 54.4, 53.7, 32.3, 25.4, 21.9, 19.5. Elemental analysis for C_24_H_24_N_2_O_3_S; Calc.: C, 68.55; H, 5.75; N, 6.66%. Found: C, 68.57; H, 5.73; N, 6.67%.

*3-(1,3-Benzothiazol-2-yl)-7-hydroxy-2-methyl-8-[(4-methylpiperidin-1-yl)methyl]-4H-chromen-4-one* (**7e**). Yield 85%, m.p. 216–218 °C, colorless. LC-MS: *m*/*z* 421.0 [M + H]^+^. ^1^H-NMR (400 MHz, CDCl_3_): δ ppm 8.13 (d, *J* = 8.8 Hz, 1H), 8.06 (d, *J* = 8.1 Hz, 1H), 7.99 (d, *J* = 7.8 Hz, 1H), 7.54–7.46 (m, 1H), 7.45–7.34 (m, 1H), 6.92 (d, *J* = 8.8 Hz, 1H), 4.07 (s, 2H), 3.16–3.05 (m, 2H), 3.04 (s, 3H), 2.43–2.20 (m, 2H), 1.85–1.72 (m, 2H), 1.60–1.48 (m, 1H), 1.45–1.30 (m, 2H), 1.00 (d, *J* = 6.4 Hz, 3H). ^13^C-NMR (100 MHz, CDCl_3_): δ ppm 175.0, 168.0, 164.8, 160.4, 154.3, 151.7, 136.0, 126.5, 125.7, 124.8, 122.8, 121.3, 116.1, 115.5, 115.0, 107.0, 54.2, 53.6, 34.0, 30.4, 22.0, 21.6. Elemental analysis for C_24_H_24_N_2_O_3_S; Calc.: C, 68.55; H, 5.75; N, 6.66%. Found: C, 68.56; H, 5.73; N, 6.65%.

*3-(1,3-Benzothiazol-2-yl)-7-hydroxy-2-methyl-8-(morpholin-4-ylmethyl)-4H-chromen-4-one* (**7f**), pale yellow crystals. Yield 88%, m.p. 243–244 °C. LC-MS: *m*/*z* 409.2 [M + H]^+^. ^1^H-NMR (400 MHz, CDCl_3_): δ ppm 8.14 (d, *J* = 8.8 Hz, 1H), 8.05 (d, *J* = 8.0 Hz, 1H), 7.98 (d, *J* = 7.8 Hz, 1H), 7.54–7.45 (m, 1H), 7.44–7.35 (m, 1H), 6.92 (d, *J* = 8.8 Hz, 1H), 4.07 (s, 2H), 3.97–3.67 (m, 4H), 3.04 (s, 3H), 2.88–2.53 (m, 4H). ^13^C-NMR (100 MHz, CDCl_3_): δ ppm 175.1, 168.1, 163.8, 160.3, 154.5, 151.7, 136.0, 127.1, 125.8, 124.9, 122.9, 121.4, 116.1, 115.8, 115.6, 106.5, 66.7, 54.2, 53.2, 22.0. Elemental analysis for C_22_H_20_N_2_O_4_S; Calc.: C, 64.69; H, 4.94; N, 6.86%. Found: C, 64.67; H, 4.96; N, 6.85%.

*3-(1,3-Benzothiazol-2-yl)-7-hydroxy-2-methyl-8-[(4-methylpiperazin-1-yl)methyl]-6-propyl-4H-chromen-4-one* (**7g**), colorless. Yield 68%, m.p. 230–231 °C. LC-MS: *m*/*z* 464.2 [M + H]^+^. ^1^H-NMR (400 MHz, CDCl_3_): δ ppm 8.04 (d, *J* = 8.1 Hz, 1H), 8.00–7.94 (m, 2H), 7.51–7.43 (m, 1H), 7.41–7.34 (m, 1H), 4.06 (s, 2H), 3.03 (s, 3H), 2.99–2.40 (m, 10H), 2.35 (s, 3H), 1.80–1.59 (m, 2H), 0.98 (t, *J* = 7.3 Hz, 3H). ^13^C-NMR (125 MHz, CDCl_3_): δ ppm 175.0, 167.8, 162.4, 160.5, 152.8, 151.6, 136.0, 129.5, 125.6, 125.3, 124.6, 122.7, 121.2, 115.3, 114.6, 106.0, 54.7, 53.8, 52.6, 45.9, 31.7, 22.5, 22.0, 14.0. Elemental analysis for C_26_H_29_N_3_O_3_S; Calc.: C, 67.36; H, 6.31; N, 9.06%. Found: C, 67.32; H, 6.29; N, 9.07%.

*2-Amino-3-(1,3-benzothiazol-2-yl)-7-hydroxy-8-[(4-methylpiperazin-1-yl)methyl]-4H-chromen-4-one* (**7h**), yellow. Yield 59%, m.p. 248–250 °C. LC-MS: *m*/*z* 423.2 [M + H]^+^. ^1^H-NMR (400 MHz, DMSO-*d*_6_): δ ppm 10.76 (s, 1H), 9.39 (s, 1H), 8.02 (d, *J* = 7.4 Hz, 1H), 7.94 (d, *J* = 7.8 Hz, 1H), 7.89 (d, *J* = 8.4 Hz, 1H), 7.50–7.41 (m, 1H), 7.38–7.29 (m, 1H), 6.84 (d, *J* = 8.3 Hz, 1H), 4.02 (s, 2H), 2.70–2.57 (m, 4H), 2.46–2.29 (m, 4H), 2.18 (s, 3H). J^13^C-NMR (125 MHz, CDCl_3_): δ ppm 172.3, 163.3, 162.7, 162.3, 151.2, 149.9, 132.9, 125.7, 125.3, 123.6, 121.3, 120.5, 113.7, 113.0, 108.1, 91.7, 54.4, 52.1, 51.5, 45.5. Elemental analysis for C_22_H_24_N_4_O_3_S; Calc.: C, 62.54; H, 5.25; N, 13.26%. Found: C, 62.52; H, 5.27; N, 13.24%.

*3-(1,3-Benzothiazol-2-yl)-7-hydroxy-8-{[4-(2-hydroxyethyl)piperazin-1-yl]methyl}-2-methyl-6-propyl-4H-chromen-4-one* (**7i**), colorless. Yield 76%, m.p. 203–204 °C. LC-MS: *m*/*z* 494.0 [M + H]^+^. ^1^H-NMR (500 MHz, CDCl_3_): δ ppm 8.05 (d, *J* = 8.1 Hz, 1H), 8.01–7.96 (m, 2H), 7.52–7.44 (m, 1H), 7.45–7.34 (m, 1H), 4.08 (s, 2H), 3.69–3.62 (m, 2H), 3.04 (s, 3H), 3.00–2.28 (m, 13H), 1.75–1.60 (m, 2H), 0.99 (t, *J* = 7.3 Hz, 3H). ^13^C-NMR (125 MHz, CDCl_3_): δ ppm 175.2, 167.8, 162.4, 160.7, 153.0, 151.8, 136.1, 129.6, 125.7, 125.7, 124.8, 122.8, 121.4, 115.6, 114.8, 106.0, 59.2, 57.9, 53.8, 52.7, 52.6, 31.8, 22.6, 22.0, 14.1. Elemental analysis for C_27_H_31_N_3_O_4_S; Calc.: C, 65.70; H, 6.33; N, 8.51%. Found: C, 65.68; H, 6.35; N, 8.53%.

*3-(1,3-Benzothiazol-2-yl)-6-ethyl-7-hydroxy-8-[(1,3,3-trimethyl-6-azabicyclo [3.2.1]oct-6-yl)methyl]-4H-chromen-4-one* (**7j**), pale yellow. Yield 84%, m.p. 183–185 °C. LC-MS: *m*/*z* 489.2 [M + H]^+^. ^1^H-NMR (300 MHz, CDCl_3_): δ ppm 9.14 (s, 1H), 8.07 (s, 1H), 8.05–7.94 (m, 2H), 7.55–7.44 (m, 1H), 7.42–7.33 (m, 1H), 4.31 (d, *J* = 14.3 Hz, 1H), 4.04 (d, *J* = 14.3 Hz, 1H), 3.36–3.28 (m, 1H), 3.27–3.16 (m, 1H), 2.83–2.62 (m, 2H), 2.33–2.24 (m, 1H), 1.93–1.71 (m, 2H), 1.59–1.37 (m, 2H), 1.38–1.23 (m, 7H), 1.13 (s, 4H), 0.96 (s, 3H). ^13^C-NMR (125 MHz, CDCl_3_): δ ppm 174.4, 164.4, 159.5, 155.1, 153.5, 151.8, 136.4, 132.1, 126.1, 124.7, 124.5, 122.4, 121.7, 117.6, 115.2, 108.0, 64.8, 62.6, 54.2, 51.5, 44.0, 41.7, 40.7, 36.9, 32.2, 29.6, 25.9, 22.9, 13.8. Elemental analysis for C_29_H_32_N_2_O_3_S; Calc.: C, 71.26; H, 6.60; N, 5.73%. Found: C, 71.25; H, 6.58; N, 5.76%.

*3-(1,3-Benzothiazol-2-yl)-7-hydroxy-2-methyl-8-[(1,3,3-trimethyl-6-azabicyclo [3.2.1]oct-6-yl)methyl]-4H-chromen-4-one* (**7k**), colorless. Yield 87%, m.p. 158–159 °C. LC-MS: *m*/*z* 475.2 [M + H]^+^. ^1^H-NMR (400 MHz, CDCl_3_): δ ppm 10.43 (s, 1H), 8.12 (d, *J* = 8.8 Hz, 1H), 8.04 (d, *J* = 8.0 Hz, 1H), 7.97 (d, *J* = 7.9 Hz, 1H), 7.52–7.43 (m, 1H), 7.43–7.33 (m, 1H), 6.91 (d, *J* = 8.8 Hz, 1H), 4.28 (d, *J* = 14.5 Hz, 1H), 4.00 (d, *J* = 14.5 Hz, 1H), 3.34 (d, *J* = 11.0 Hz, 1H), 3.27–3.16 (m, 1H), 3.02 (s, 3H), 2.32–2.22 (m, 1H), 1.92–1.78 (m, 2H), 1.59–1.48 (m, 1H), 1.47–1.40 (m, 1H), 1.36–1.28 (m, 2H), 1.26 (s, 3H), 1.14 (s, 3H), 0.97 (s, 3H); ^13^C-NMR (125 MHz, CDCl_3_): δ ppm 175.0, 167.8, 165.6, 160.5, 154.1, 151.7, 136.1, 126.7, 125.7, 124.8, 122.8, 121.3, 116.3, 115.5, 114.7, 108.1, 65.1, 62.7, 54.4, 51.5, 43.9, 41.7, 40.6, 36.9, 32.1, 29.6, 25.9, 22.0. Elemental analysis for C_28_H_30_N_2_O_3_S; Calc.: C, 70.86; H, 6.37; N, 5.90%. Found: C, 70.90; H, 6.38; N, 5.88%.

*Ethyl 3-(1,3-benzothiazol-2-yl)-6-ethyl-7-hydroxy-4-oxo-8-[(1,3,3-trimethyl-6-azabicyclo[3.2.1]oct-6-yl)methyl]-4H-chromene-2-carboxylate* (**7l**), colorless. Yield 78%, m.p. 153–154 °C. LC-MS: *m*/*z* 561.2 [M + H]^+^. ^1^H-NMR (400 MHz, CDCl_3_): δ ppm 12.15 (s, 1H), 8.04 (s, 1H), 8.01–7.92 (m, 2H), 7.52–7.44 (m, 1H), 7.39 (t, *J* = 7.5 Hz, 1H), 4.51 (q, *J* = 7.2 Hz, 2H), 4.32 (d, *J* = 14.4 Hz, 1H), 4.05 (d, *J* = 14.4 Hz, 1H), 3.35 (d, *J* = 11.0 Hz, 1H), 3.28–3.17 (m, 1H), 2.88–2.63 (m, 2H), 2.31 (d, *J* = 11.0 Hz, 1H), 1.96–1.70 (m, 2H), 1.60–.50 (m, 1H), 1.48–1.39 (m, 1H), 1.35–1.28 (m, 8H), 1.27 (s, 3H), 1.13 (s, 3H), 0.97 (s, 3H); ^13^C-NMR (125 MHz, CDCl_3_): δ ppm 174.6, 165.2, 162.2, 157.7, 153.9, 152.9, 151.6, 136.6, 132.7, 126.0, 125.0, 124.4, 122.9, 121.7, 114.9, 114.5, 107.6, 64.7, 63.1, 62.6, 54.1, 51.5, 43.8, 41.7, 40.6, 36.9, 32.2, 29.6, 25.8, 22.9, 13.9, 13.7. Elemental analysis for C_32_H_36_N_2_O_5_S; Calc.: C, 68.55; H, 6.47; N, 5.00%. Found: C, 68.53; H, 6.51; N, 5.03%.

*2-Amino-6-ethyl-7-hydroxy-3-(4-methyl-1,3-thiazol-2-yl)-8-(piperidin-1-ylmethyl)-4H-chromen-4-one* (**7m**), yellow. Yield 49%, m.p. 174–175 °C. LC-MS: *m*/*z* 400.2 [M + H]^+^. ^1^H-NMR (500 MHz, CDMSO-*d*_6_): δ ppm 10.47 (s, 1H), 9.00 (s, 1H), 7.70 (s, 1H), 6.99 (s, 1H), 4.05 (s, 2H), 2.66–2.51 (m, 6H), 2.40 (s, 3H), 1.67–1.55 (m, 4H), 1.53–1.42 (m, 2H), 1.17 (t, *J* = 7.5 Hz, 3H). ^13^C-NMR (125 MHz, CDCl_3_): δ ppm 173.5, 162.5, 161.6, 161.5, 149.6, 148.5, 129.4, 124.1, 113.2, 111.7, 106.0, 93.6, 54.5, 53.9, 25.7, 23.8, 22.7, 17.2, 13.7. Elemental analysis for C_21_H_25_N_3_O_3_S; Calc.: C, 63.13; H, 6.31; N, 10.52%. Found: C, 63.10; H, 6.15; N, 10.50%.

#### 3.2.4. General Procedure for Synthesis of Coumarin Aminomethyl Derivatives **10**,**11**

A stirring mixture of the corresponding 3-hetarylcoumarin **9** or **10a,b** chromone (1 mmol) and aminal (1.2 mmol) in 1,4-dioxane (5 mL) was refluxed for 6–8 h. The reaction mixture was cooled, diluted with 10 mL of hexane. The formed precipitate was filtered off, dried, and re-crystallized from toluene-hexane mixture to furnish the desired chromone Mannich bases **7**.

*3-(1,3-Benzothiazol-2-yl)-8-{[bis(2-hydroxyethyl)amino]methyl}-7-hydroxy-2H-chromen-2-one* (**10a**). Yield 56%, m.p. 186–187 °C, yellow. LC-MS: *m*/*z* 413.2 [M + H]^+^. ^1^H-NMR (400 MHz, DMSO-*d*_6_): δ ppm 9.01 (s, 1H), 8.09 (d, *J* = 7.9 Hz, 1H), 7.98 (d, *J* = 8.1 Hz, 1H), 7.74 (d, *J* = 8.7 Hz, 1H), 7.54–7.48 (m, 1H), 7.45–7.32 (m, 1H), 6.69 (d, *J* = 8.6 Hz, 1H), 4.23 (s, 2H), 3.65 (t, *J* = 5.5 Hz, 4H), 2.87 (t, *J* = 5.5 Hz, 4H). ^13^C-NMR (125 MHz, CDCl_3_): δ ppm 168.4, 160.9, 159.8, 153.9, 152.1, 142.9, 135.4, 130.8, 126.3, 124.6, 122.0, 121.9, 116.0, 111.4, 109.6, 107.4, 57.3, 55.1, 49.8. Elemental analysis for C_21_H_20_N_2_O_5_S; Calc.: C, 61.15; H, 4.89; N, 6.79%. Found: C, 61.12; H, 4.91; N, 6.77%.

*3-(1,3-Benzothiazol-2-yl)-8-[(3,3-dimethylpiperidin-1-yl)methyl]-7-hydroxy-2H-chromen-2-one* (**10b)**. Yield 85%, m.p. 188–189 °C, green (fluorescent). LC-MS: *m*/*z* 421.0 [M + H]^+^. ^1^H-NMR (500 MHz, CDCl_3_): δ ppm 8.97 (s, 1H), 8.05 (d, *J* = 8.1 Hz, 1H), 7.95 (d, *J* = 7.8 Hz, 1H), 7.54–7.47 (m, 2H), 7.41–7.35 (m, 1H), 6.83 (d, *J* = 8.6 Hz, 1H), 4.17–3.95 (m, 2H), 3.19–2.45 (m, 2H), 2.32–1.91 (m, 1H), 1.91–1.16 (m, 5H), 1.02 (s, 6H). ^13^C-NMR (125 MHz, CDCl_3_): δ ppm 166.0, 161.0, 160.4, 153.5, 152.6, 142.8, 136.6, 130.1, 126.4, 125.0, 122.6, 121.8, 115.5, 114.8, 111.3, 107.5, 65.8, 54.7, 53.8, 36.7, 31.2, 21.9. Elemental analysis for C_24_H_24_N_2_O_3_S; Calc.: C, 68.55; H, 5.75; N, 6.66%. Found: C, 68.52; H, 5.77; N, 6.67%.

*3-(1,3-Benzothiazol-2-yl)-7-hydroxy-8-{[4-(2-hydroxyethyl)piperazin-1-yl]methyl}-2H-chromen-2-one* (**10c**). Yield 64%, m.p. 207–208 °C, yellow. LC-MS: *m*/*z* 438.0 [M + H]^+^. ^1^H-NMR (400 MHz, DMSO-*d*_6_): δ ppm 9.06 (s, 1H), 8.11 (d, *J* = 7.9 Hz, 1H), 8.00 (d, *J* = 8.1 Hz, 1H), 7.79 (d, *J* = 8.6 Hz, 1H), 7.56–7.48 (m, 1H), 7.45–7.37 (m, 1H), 6.79 (d, *J* = 8.6 Hz, 1H), 3.99 (s, 2H), 3.50 (t, *J* = 6.0 Hz, 2H), 2.80–2.53 (m, 8H), 2.45–2.36 (m, 2H). ^13^C-NMR (125 MHz, DMSO-*d*_6_): δ ppm 165.9, 160.6, 159.7, 153.7, 152.0, 142.9, 135.5, 130.8, 126.4, 124.8, 122.0, 118.8, 115.2, 112.7, 110.4, 107.4, 59.8, 58.4, 52.6, 51.9, 51.7. Elemental analysis for C_23_H_23_N_3_O_4_S; Calc.: C, 63.14; H, 5.30; N, 9.60%. Found: C, 63.12; H, 5.28; N, 9.63%.

*7-Hydroxy-8-{[4-(2-hydroxyethyl)piperazin-1-yl]methyl}-3-(4-methyl-1,3-thiazol-2-yl)-2H-chromen-2-one* (**11a**). Yield 58%, m.p. 157–159 °C, yellow. LC-MS: *m*/*z* 402.2 [M + H]^+^. ^1^H-NMR (500 MHz, CDCl_3_): δ ppm 8.74 (s, 1H), 7.45 (d, *J* = 8.6 Hz, 1H), 7.04–6.98 (m, 1H), 6.81 (d, *J* = 8.6 Hz, 1H), 4.11 (s, 2H), 3.68–3.60 (m, 2H), 3.20–2.53 (m, 10H), 2.52 (s, 3H). ^13^C-NMR (125 MHz, CDCl_3_): δ ppm 163.8, 160.2, 159.2, 152.9, 152.8, 139.9, 129.6, 116.4, 115.8, 115.0, 111.8, 107.4, 59.3, 58.0, 53.7, 52.8, 52.6, 17.3. Elemental analysis for C_20_H_23_N_3_O_4_S; Calc.: C, 59.83; H, 5.77; N, 10.47%. Found: C, 59.85; H, 5.79; N, 10.44%.

*3-[4-(4-Bromophenyl)-1,3-thiazol-2-yl]-7-hydroxy-8-{[4-(2-hydroxyethyl)piperazin-1-yl]methyl}-2H-chromen-2-one* (**11b**). Yield 71%, m.p. 224–225 °C, colorless. LC-MS: *m*/*z* 542.0 [M + H]^+^. ^1^H-NMR (300 MHz, CDCl_3_): δ ppm 8.88 (s, 1H), 7.88 (d, *J* = 8.5 Hz, 2H), 7.62 (s, 1H), 7.58 (d, *J* = 8.6 Hz, 2H), 7.52 (d, *J* = 8.6 Hz, 1H), 6.85 (d, *J* = 8.6 Hz, 1H), 4.13 (s, 2H), 3.68–3.59 (m, 2H), 3.03–2.29 (m, 10H). ^13^C-NMR (100 MHz, CDCl_3_): δ ppm 164.1, 160.2, 159.8, 154.1, 152.9, 140.5, 133.5, 131.9, 129.7, 128.0, 122.2, 115.8, 115.5, 115.1, 111.7, 107.4, 59.2, 57.9, 53.7, 52.8, 52.6. Elemental analysis for C_25_H_24_BrN_3_O_4_S; Calc.: C, 55.35; H, 4.46; N, 7.75%. Found: C, 55.32; H, 4.49; N, 7.73%.

*3-[4-(4-Bromophenyl)-1,3-thiazol-2-yl]-7-hydroxy-8-[(2-methylpiperidin-1-yl)methyl]-2H-chromen-2-one* (**11c**). Yield 68%, m.p. 184–186 °C, yellow. LC-MS: *m*/*z* 511.0 [M + H]^+^. ^1^H-NMR (300 MHz), δ 11.31 (s, 1H), 8.85 (s, 1H), 7.87 (d, *J* = 8.5 Hz, 2H), 7.61–7.54 (m, 3H), 7.47 (d, *J* = 8.6 Hz, 1H), 6.79 (d, *J* = 8.6 Hz, 1H), 4.55–4.23 (m, 1H), 4.08–3.91 (m, 1H), 3.20–2.15 (m, 3H), 1.91–1.34 (m, 6H), 1.25 (d, *J* = 5.5 Hz, 3H). ^13^C-NMR (125 MHz, CDCl_3_–TFA 1:1): δ ppm 165.1, 160.6, 155.7, 148.4, 148.1, 135.8, 133.8, 129.5, 128.7, 127.7, 125.1, 118.1, 116.8, 112.7, 109.0, 105.0, 64.4, 54.0, 47.4, 32.1, 23.3, 21.8, 18.1. Elemental analysis for C_25_H_23_BrN_2_O_3_S; Calc.: C, 58.71; H, 4.53; N, 5.48%. Found: C, 58.68; H, 4.50; N, 5.50%.

*3-[4-(4-Bromophenyl)-1,3-thiazol-2-yl]-7-hydroxy-8-[(3-methylpiperidin-1-yl)methyl]-2H-chromen-2-one* (**11d**). Yield 83%, m.p. 216–217 °C, yellow. LC-MS: *m*/*z* 511.0 [M + H]^+^. ^1^H-NMR (300 MHz, CDCl_3_): δ ppm 11.45 (s, 1H), 8.84 (s, 1H), 7.87 (d, *J* = 8.5 Hz, 2H), 7.61–7.53 (m, 3H), 7.48 (d, *J* = 8.6 Hz, 1H), 6.81 (d, *J* = 8.6 Hz, 1H), 4.06 (s, 2H), 3.10–2.85 (m, 2H), 2.39–1.52 (m, 6H), 1.15–0.73 (m, 4H); ^13^C-NMR (125 MHz, DMSO-*d*_6_): δ ppm 162.4, 159.4, 159.2, 154.5, 153.2, 140.5, 133.3, 132.8, 131.9, 128.4, 121.6, 117.4, 115.2, 114.1, 111.7, 103.6, 58.5, 52.4, 48.7, 29.7, 29.0, 22.7, 18.8. Elemental analysis for C_25_H_23_BrN_2_O_3_S; Calc.: C, 58.71; H, 4.53; N, 5.48%. Found: C, 58.72; H, 4.51; N, 5.50%.

*3-[4-(4-Bromophenyl)-1,3-thiazol-2-yl]-7-hydroxy-8-[(4-methylpiperidin-1-yl)methyl]-2H-chromen-2-one* (**11e**). Yield 85%, m.p. 229–230 °C, yellow. LC-MS: *m*/*z* 511.0 [M + H]^+^. ^1^H-NMR (400 MHz, DMSO-*d*_6_): δ ppm 8.94 (s, 1H), 8.18 (s, 1H), 8.03 (d, *J* = 8.1 Hz, 2H), 7.73 (d, *J* = 8.7 Hz, 1H), 7.66 (d, *J* = 8.2 Hz, 2H), 6.75 (d, *J* = 8.7 Hz, 1H), 4.07 (s, 2H), 3.08–2.97 (m, 2H), 2.48–2.36 (m, 2H), 1.79–1.65 (m, 2H), 1.58–1.42 (m, 1H), 1.33–1.16 (m, 2H), 0.94 (d, *J* = 6.1 Hz, 3H). ^13^C-NMR (125 MHz, CDCl_3_–TFA 1:1): δ ppm 165.3, 164.9, 160.6, 155.6, 148.4, 148.0, 135.8, 133.8, 128.7, 127.7, 125.0, 118.0, 116.8, 112.5, 108.8, 104.5, 55.3, 50.3, 31.6, 29.0, 20.3. Elemental analysis for C_25_H_23_BrN_2_O_3_S; Calc.: C, 58.71; H, 4.53; N, 5.48%. Found: C, 58.70; H, 4.54; N, 5.51%.

*7-Hydroxy-3-(4-methyl-1,3-thiazol-2-yl)-8-[(1,3,3-trimethyl-6-azabicyclo [3.2.1]oct-6-yl)methyl]-2H-chromen-2-one* (**11f**). Yield 70%, m p. 159–160 °C, yellow (fluorescent). LC-MS: *m*/*z* 425.0 [M + H]^+^. ^1^H-NMR (500 MHz, CDCl_3_): δ ppm 8.73 (s, 1H), 7.44 (d, *J* = 8.6 Hz, 1H), 6.99 (s, 1H), 6.80 (d, *J* = 8.6 Hz, 1H), 4.37 (d, *J* = 14.6 Hz, 1H), 4.07 (d, *J* = 14.6 Hz, 1H), 3.36 (d, *J* = 11.2 Hz, 1H), 3.30–3.19 (m, 1H), 2.52 (s, 3H), 2.30 (d, *J* = 11.2 Hz, 1H), 1.96–1.76 (m, 2H), 1.53 (d, *J* = 14.0 Hz, 1H), 1.42 (d, *J* = 13.7 Hz, 1H), 1.32 (dd, *J* = 14.3, 2.2 Hz, 1H), 1.28 (d, *J* = 11.5 Hz, 1H), 1.24 (s, 3H), 1.12 (s, 3H), 0.95 (s, 3H). ^13^C-NMR (125 MHz, CDCl_3_): δ ppm 166.6, 160.5, 159.6, 152.8, 152.8, 140.4, 129.8, 116.0, 115.7, 114.6, 110.7, 108.1, 64.9, 63.1, 54.8, 51.5, 43.8, 41.7, 40.6, 36.9, 32.1, 29.6, 25.8, 17.4. Elemental analysis for C_24_H_28_rN_2_O_3_S; Calc.: C, 67.90; H, 6.65; N, 6.60%. Found: C, 67.88; H, 6.67; N, 6.63%.

## 4. Biological Evaluation

### 4.1. Cell Viability Studies

A set of 25 compounds was tested for cell viability studies using MTT assay, in HCT116 and HeLa cell lines. All experiments were carried out in triplicate as per previously reported methods [53,54]. For the viability studies, HCT116 and HeLa cells were seeded at the count of 7 × 10^3^ cells per well in a transparent 96-well plates, and incubated for 24 h at 37 °C, in a humidified incubator with 5% CO_2_. Following this, the cells were treated with the compounds at a final concentration of 10 µM and incubated further for 72 h at 37 °C. After incubation, MTT [3-(4,5-Dimethyl-2-thiazolyl)-2,5-diphenyl tetrazolium bromide] was added at a final concentration of 0.5 mg/mL. The cells were further incubated for 4 h at 37 °C in an incubator. The formazan crystals formed were dissolved using 50 µL of DMSO. Thereafter, the absorbance was measured at 570 nm using the PerkinElmer EnVision Multilabel Reader (Perkin Elmer, Inc., Waltham, MA, USA). The cells treated with DMSO were used as a control for all the experiments. Both the cell lines showed viability ≤ 50% for three compounds in the initial screening, namely **2c**, **7h** and **7l**. Hence, these three compounds were selected from the initial screening and were sensitized against HCT116 and HeLa, across a dose range of 0–100 µM by performing another cell viability assay. Finally, the results obtained were plotted using Graphpad Prism 8.0.2 to obtain the respective IC_50_ values.

### 4.2. Immunoblot Assay

The immunoblot assay was carried out as per previous reports [2]. HCT116 and HeLa cells were seeded at the count of 0.5 × 10^5^ cells per ml in a 6-well plate and incubated overnight at 37 °C, in a humidified CO_2_ incubator. For this assay, the cells were treated with the hit compounds with their concentration range approximately equal to their IC_50_ values (Table 3), and incubated for 1 h at 37 °C. After the incubation, the cells were given UV radiation energy via UVP cross linker at 50 mJ/cm^2^ and further incubated for 1 h at 37 °C. The media was stored before UV radiation and added back after the treatment. The cells were then trypsinized, washed with ice cold 1X PBS, and lysed with RIPA buffer. The cell lysates were isolated by centrifugation at 4 °C, 13,500 rpm for 10 min. The protein concentration was normalized by Bradford assay. The samples were loaded on SDS-PAGE gel and subjected to immunoblotting. β-actin was taken as a loading control in the experiment.

Further, to identify the pattern of inhibition of Chk1 phosphorylation, the immunoblot assay was repeated for a range of concentrations of the compounds **2c**, **7h**, and **7l** in HeLa cell line against Chk1 and pChk1 antibodies. The experiments were conducted in a similar way for a concentration range of 2 µM, 5 µM and 10 µM. β-actin was taken as a loading control in the experiment.

### 4.3. Molecular Docking

In the present study, the compounds **2c**, **7h** and **7l** were subjected to molecular docking studies at the Torin2 binding site of homology modelled ATR kinase domain [55], using the Glide module (XP) of Maestro 12.7.156 version of Schrödinger software [56]. The homology modelled ATR kinase domain was further prepared for molecular docking, using the Protein Preparation Wizard by including Epik state penalties (pH 7.0 ± 2.0), and by performing the minimization using 0.3 Å RMSD and OPLS4 force field [57,58,59]. Further, the compounds **2c**, **7h** and **7l** were included in the project using the 2D sketcher option in the software, following which the 3D structures were incorporated in the workspace. The standard molecule for the study was selected to be Torin2, the standard inhibitor in the modelled structure, which is a well-known mTOR and ATR/ATM inhibitor known for its potency against the p-Chk1 Ser 317 and p70 S6K Thr 389 substrates in the DDR pathway through cell studies. The ligands were then prepared using the LigPrep module [57].

The ligand preparation was followed by receptor grid generation, wherein the grid was generated using the Glide module, by selecting the Torin2 ligand from the minimized protein structure. Ligand docking for the standard molecule and the synthesized compounds was conducted in the Glide XP (Extra Precision) module [56].

The docking results were analyzed in the XP Visualizer tool, wherein the binding energies were analyzed using the docking score instead of GlideScore, since the Epik state penalties were included during the protein and ligand preparations. The best pose of each of the compounds **2c**, **7h** and **7l** with higher negative value of DockScore were considered for the study.

## 5. Conclusions

A series of benzothiazole and chromone derivatives were synthesized and evaluated for their anticancer activity as inhibitors of ATR kinase. Cell viability of a set of 25 compounds was performed using MTT assay in HCT116 and HeLa cell lines, involving 72 h incubation of the compounds at a final concentration of 10 µM. Cells incubated with compounds **2c**, **7h** and **7l** were found to show a viability ≤50, and were taken forward for dose–response studies. The three compounds showed IC_50_ values in micromolar range. Among all the compounds tested, **7l** had the best IC_50_ values in both the cell lines. Compounds **2c** and **7l** were found to be equally cytotoxic towards both the cell lines, namely, HCT116 and Hela, while compound **7h** showed better cytotoxicity towards HeLa cell line.

To further identify the inhibitory potential of the synthesized compounds in the DNA damage response (DDR) signalling pathway, an immunoblot assay was carried out for these three compounds in order to analyze the inhibition of phosphorylation of Chk1 at Ser 317 in HeLa and HCT116 cells. Compound **7h** showed inhibition of pChk1 at Ser 317 in HeLa cells at a concentration of 3.995 µM. Further analysis for Chk1 and pChk1 expression was analyzed in Hela cells by treatment against all the three compounds at a range of concentrations such as 2, 5 and 10 µM, wherein compound **7h** showed Chk1 inhibition at 2 and 5 µM, while pChk1 expression was observed for compound **7l** at a concentration of 5 µM. The binding interactions of the compounds with the ATR kinase domain were studied through molecular docking, wherein compound **2c**, **7h** and **7l** showed binding interactions similar and/or lesser than the standard ligand Torin2. Thus, these compounds can serve as starting point for further modifications in order to improve the activity.

## Figures and Tables

**Table 1:** IC_50_ values of compounds in HCT116 and HeLa.

Sl. No.	Compound	IC_50_ Value (µM)	
		HCT116	HeLa
1	**2c**	3.670	2.642
2	**7h**	6.553	3.995
3	**7l**	2.527	2.659

**Table 2:** Molecular docking results for Torin2 and compounds **2c**, **7h** and **7l**.

Sl. No.	Interaction	Point Interaction	Donor Atom	Acceptor Atom	Type of Interaction	Bond Distance (Å)	Binding Energy (Dock Score) (kcal/mol)
1.	**2c**	**2c** OH–Val 88	**2c**: O	Val 88	Hydrogen bond	1.72	−5.5
		**2c** aromatic ring–Trp 87	**2c**: aromatic ring	Trp 87	π-π stacking	3.37, 3.81, 3.87	
		**2c** benzothiazole–Lys 16	**2c**: benzothiazole	Lys 16	π-Cation interaction	5.15, 5.37	
2.	**Torin2**	Lys 100–Torin2 O	Lys 100	Torin2: O	Hydrogen bond	1.80	−5.1
		Torin2 NH_2_–Val 88	Torin2: N	Val 88	Hydrogen bond	2.31	
		Lys 16–Torin2 N	Lys 16	Torin2: N	Hydrogen bond	2.01	
		Torin2 pyridine–Trp 87	Torin2: pyridine	Trp 87	π-π stacking	3.47, 3.77	
3.	**7h**	**7h** OH–Asn 89	**7h**: O	Asn 89	Hydrogen bond	1.90	−3.4
		**7h** NH–Thr 91	**7h**: N	Thr 91	Hydrogen bond	1.93	
		**7h** benzothiazole–Trp 87	**2c**: benzothiazole	Trp 87	π-π stacking	4.00	
4.	**7l**	Lys 16–7l O	Lys 16	7l: O	Hydrogen bond	1.94	−4.8
		**7l** benzothiazole–Trp 87	**7l**: benzothiazole	Trp 87	π-π stacking	5.02	
		Lys 16–**7l** benzothiazole	Lys 16	**7l**: benzothiazole	π-Cation interaction	5.01	
		Lys 100–**7l** O	Lys 100	**7l**: O	Salt Bridge	2.80	

**Table 3:** Concentration of compounds **2c**, **7h** and **7l** for immunoblot assay in HCT116 and HeLa cell lines.

Sl. No.	Compound	Concentration for Blot (µM)	
		HCT116	HeLa
1	**2c**	3.6	2.6
2	**7h**	6.5	4.0
3	**7l**	2.5	2.6

## Supplementary Materials

The following supporting information can be downloaded at: https://www.mdpi.com/article/10.3390/molecules27144637/s1. The copies of ^1^H- and ^13^C-NMR spectra for all new synthesized compounds have been submitted along with the manuscript.

## Sample Availability

Samples of the compounds are available from the authors.
