# Supplementary material for: Benzothiazole and Chromone Derivatives as Potential ATR Kinase Inhibitors and Anticancer Agents
Source: Molecules. 2022 Jul 20;27(14):4637. doi: 10.3390/molecules27144637 (PMC9324009; doi:10.3390/molecules27144637)
Supplement: Supplementary file 1 [file molecules-27-04637-s001.zip › molecules-1799118-supplementary.pdf]

Benzothiazole and Chromone Derivatives as Potential ATR Kinase Inhibitors and Anticancer Agents

Mykhaylo Frasinyuk [1,†], Chhabria Dimple [2,†], Victor Kartsev [3,‡], Dilip Haritha [2,‡], Samvel Sirakanyan [4], Sivapriya Kirubakaran [2,*], Anthi Petrou [5], Athina Geronikaki [5,*] and Domenico Spinelli [6,*]

1 V.P. Kukhar Institute of Bioorganic Chemistry and Petrochemistry, National Academy of Science of Ukraine, 02094 Kiev, Ukraine; mykhaylo.frasinyuk@ukr.net
2 Discipline of Chemistry, Indian Institute of Technology Gandhinagar, Gandhinagar 382055, India; dimple.c@iitgn.ac.in (D.C.); d_haritha@iitgn.ac.in (H.D.)
3 InterBioScreen, 119019 Chernogolovka, Russia; vkartsev@ibscreen.chg.ru
4 Scientific Technological Center of Organic and Pharmaceutical Chemistry, National Academy of Science of the Republic of Armenia, Institute of Fine Organic Chemistry, Yerevan 0014, Armenia; shnnr@mail.ru
5 School of Pharmacy, Aristotle University of Thessaloniki, 54124 Thessaloniki, Greece; anthi.petrou.thessaloniki1@gmail.com
6 Dipartimento di Chimica "G. Ciamician", Alma Mater Studiorum-Università di Bologna, 40126 Bologna, Italy

* Correspondence: priyak@iitgn.ac.in (S.K.); geronik@pharm.auth.gr (A.G.); domenico.spinelli@unibo.it (D.S.)
† These authors contributed equally to this work.
‡ These authors contributed equally to this work.

Supplementary Material

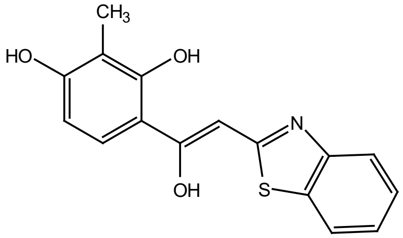

^{1}H NMR spectrum of compound **1d** in DMSO-d_6

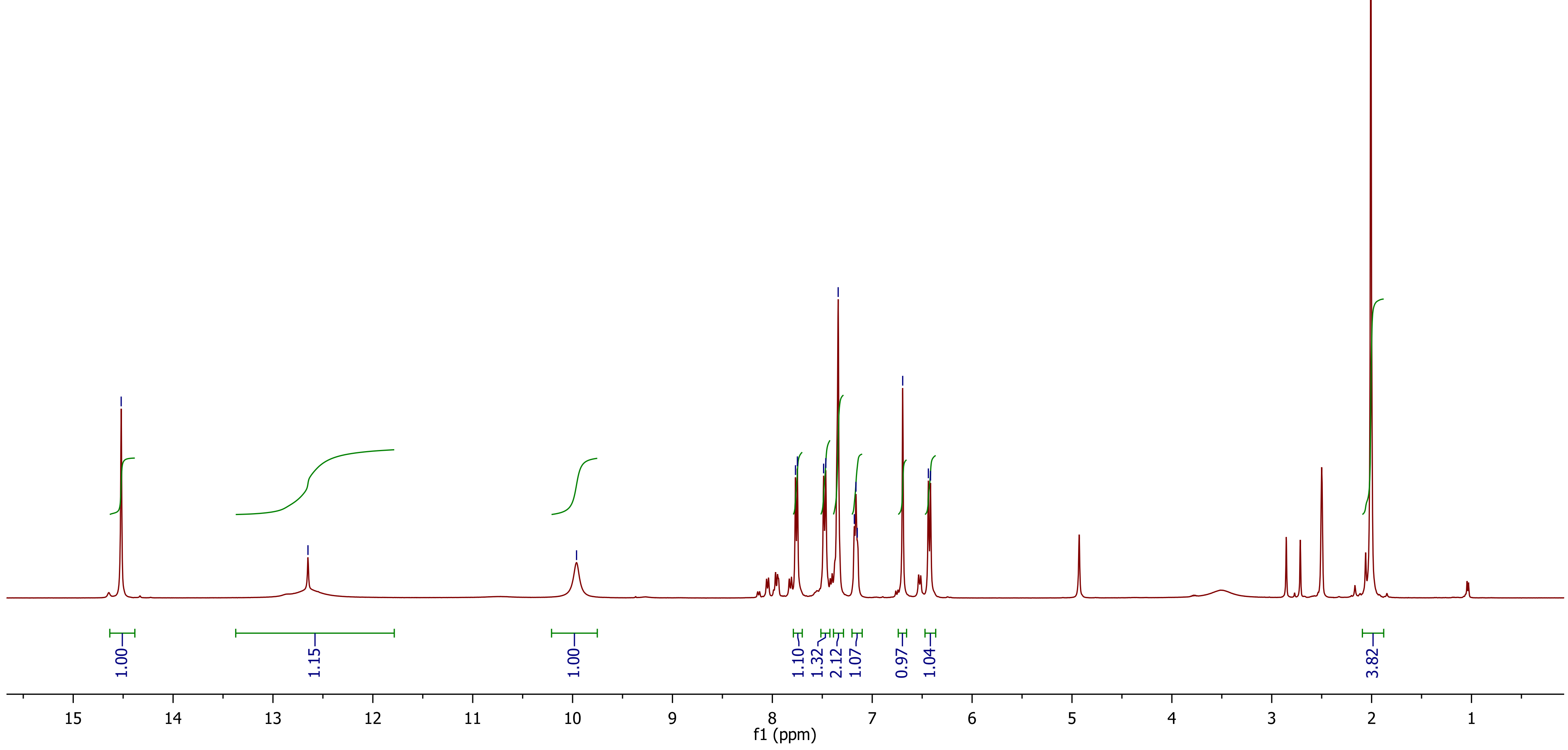

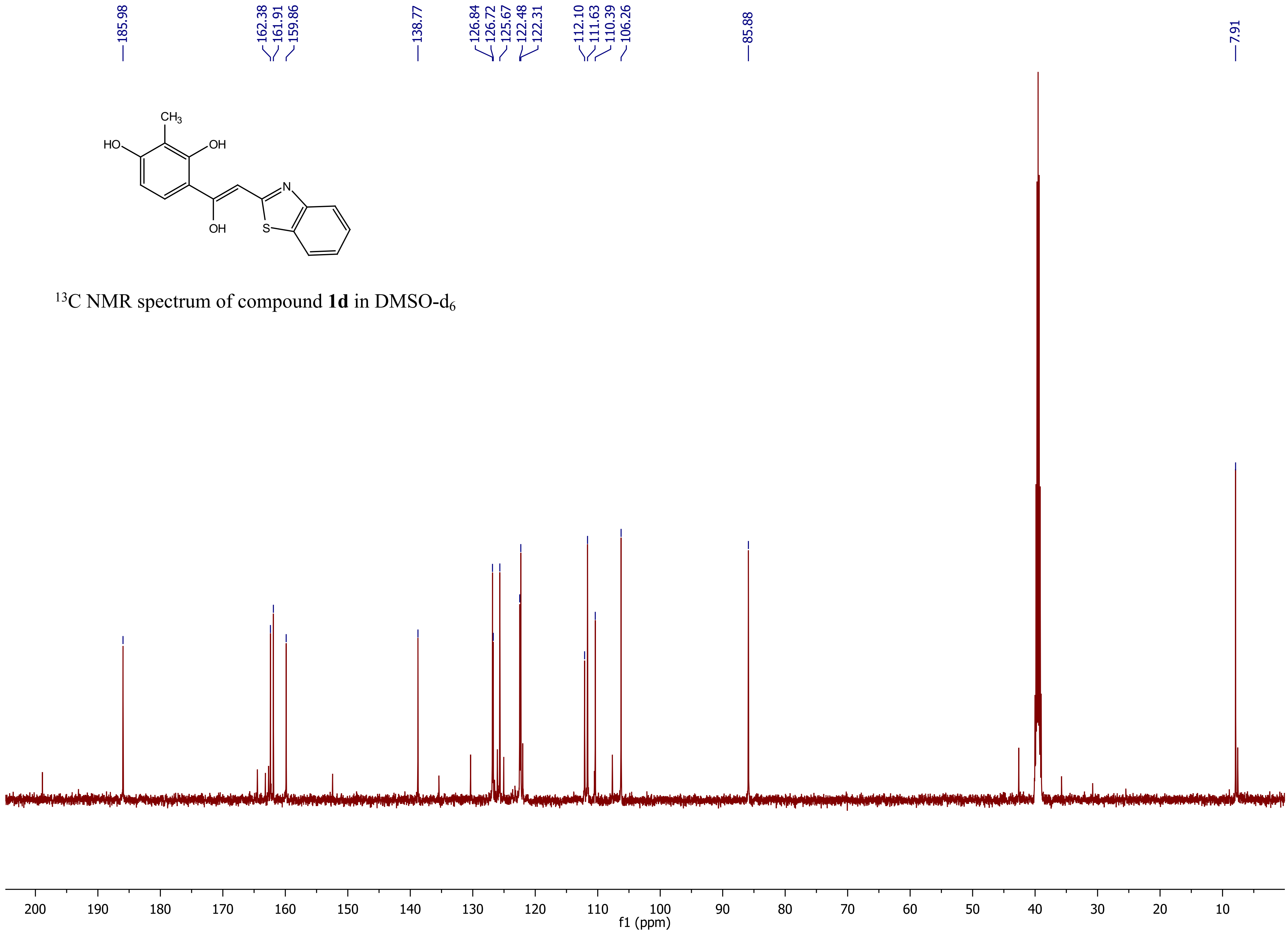

^{13}C NMR spectrum of compound **1d** in DMSO-d_6

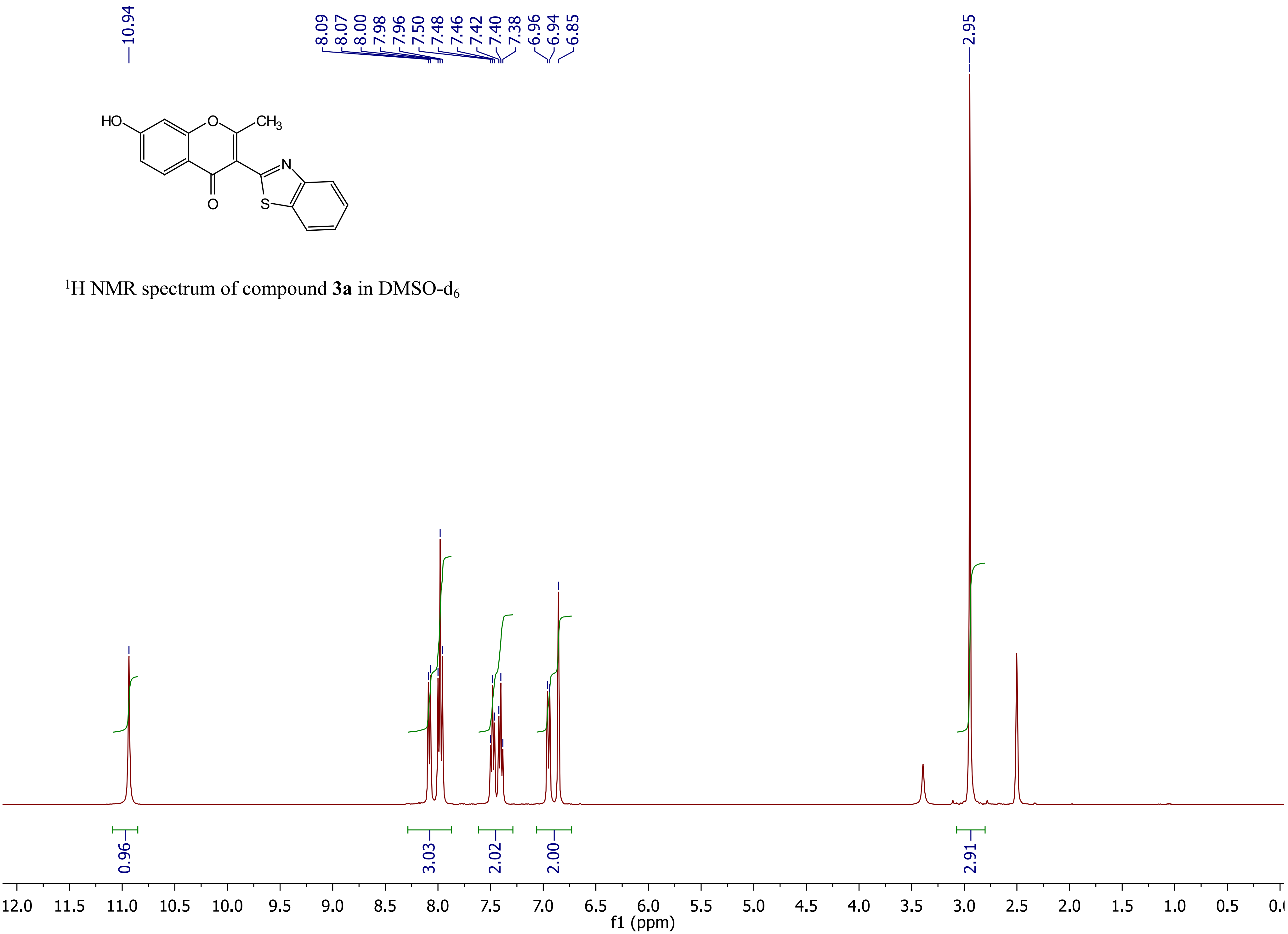

^{1}H NMR spectrum of compound **3a** in DMSO-d_6

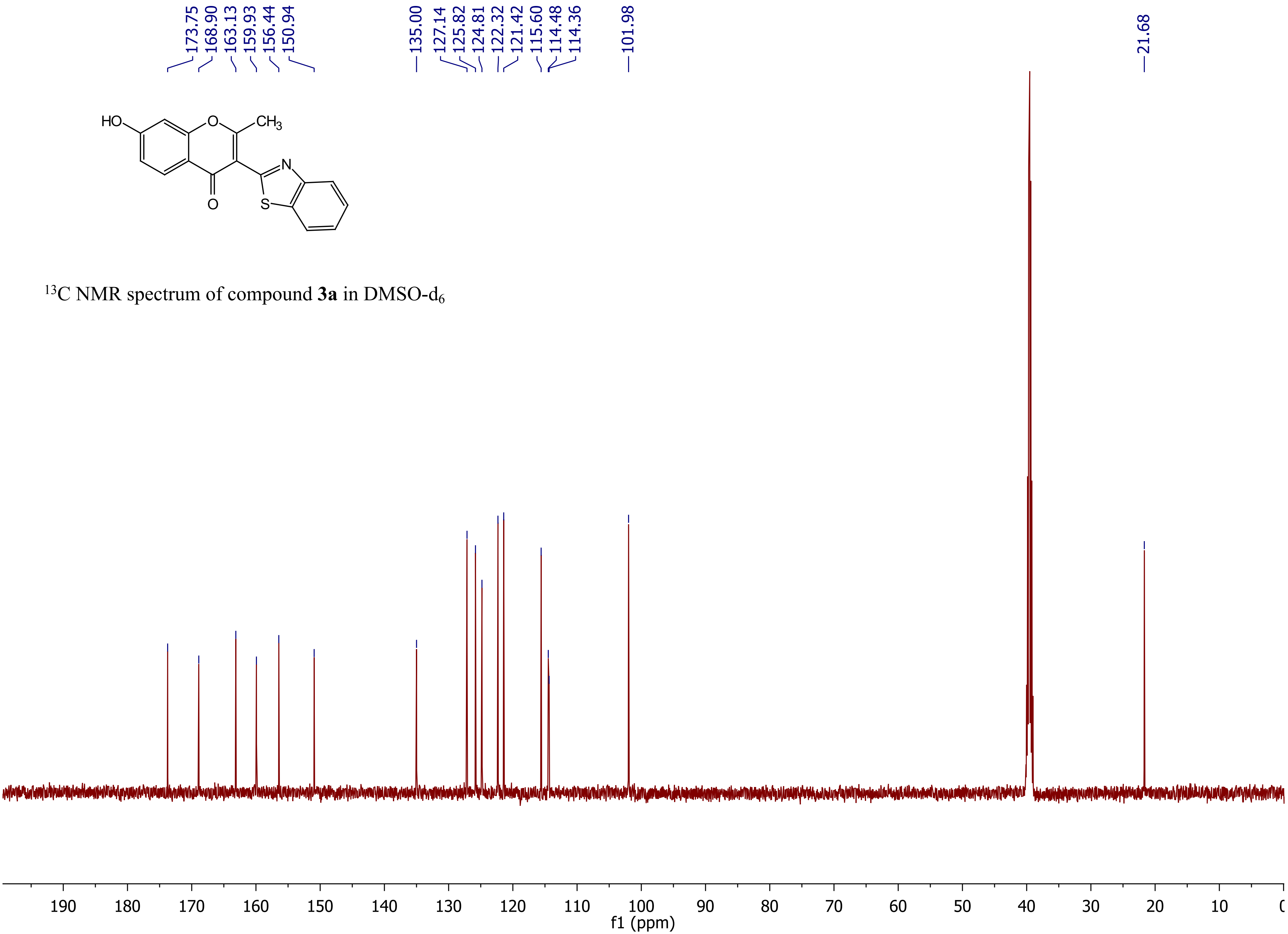

^{13}C NMR spectrum of compound **3a** in DMSO-d_6

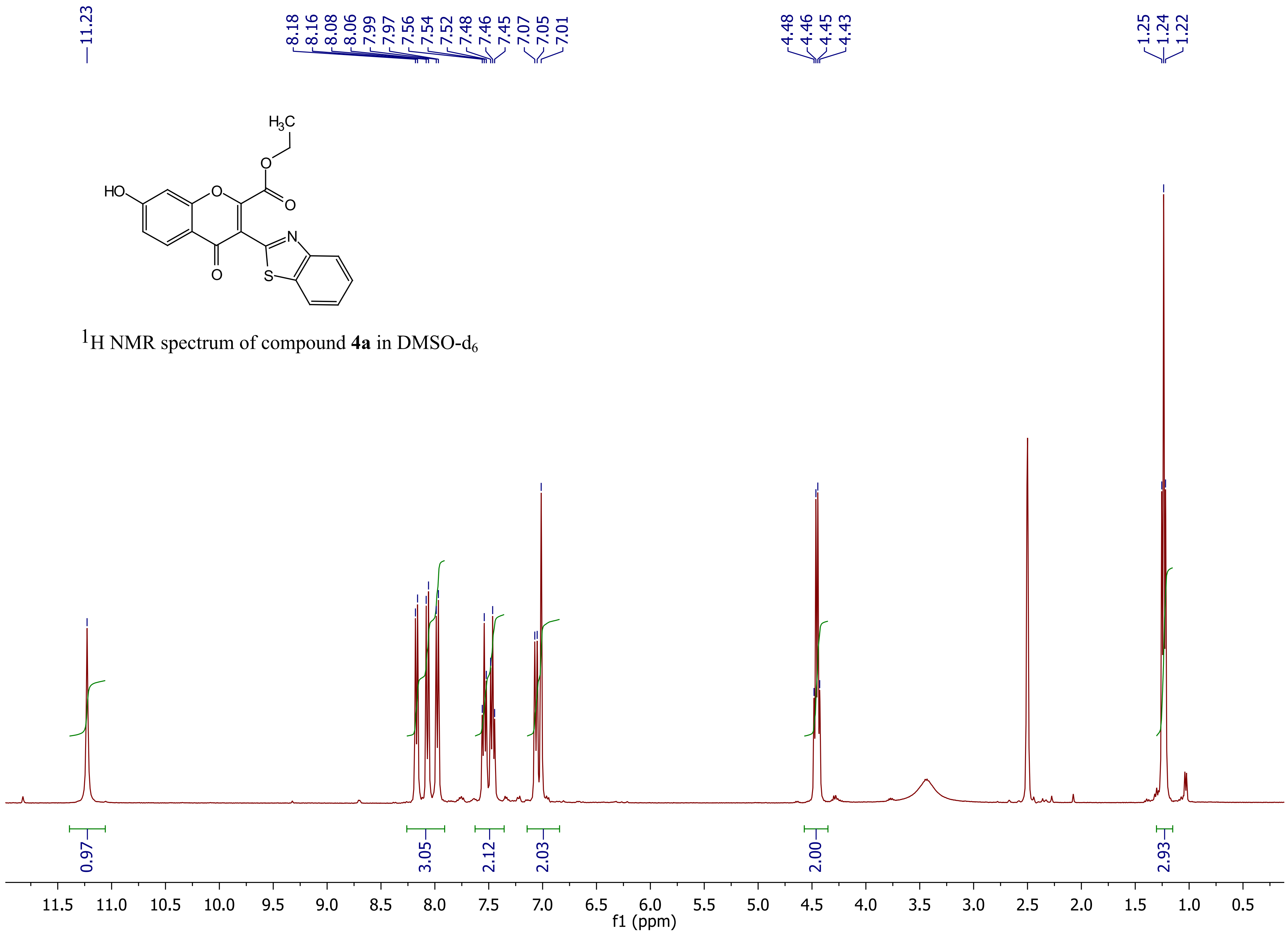

^{1}H NMR spectrum of compound **4a** in DMSO-d_6

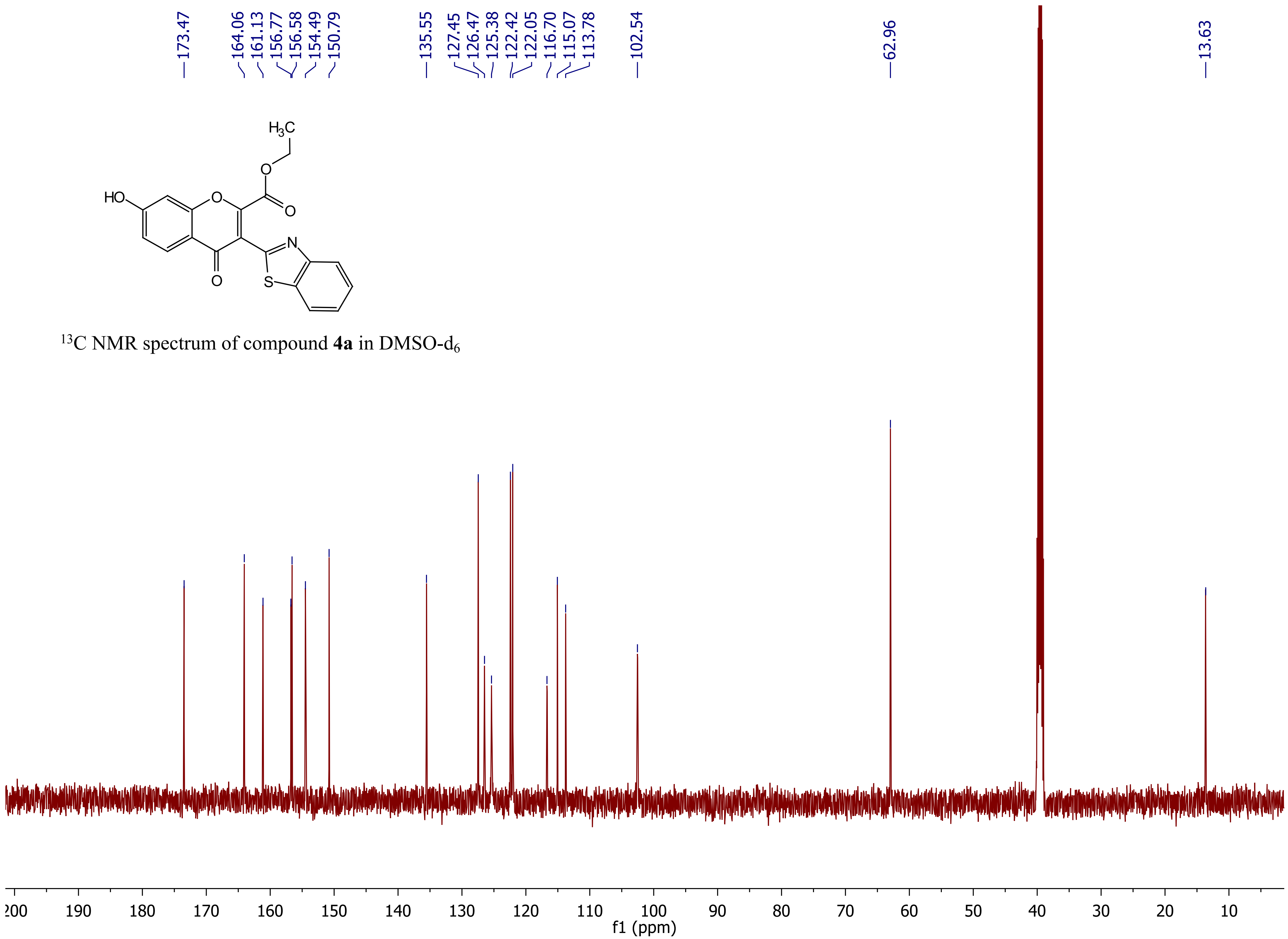

^{13}C NMR spectrum of compound **4a** in DMSO-d_6

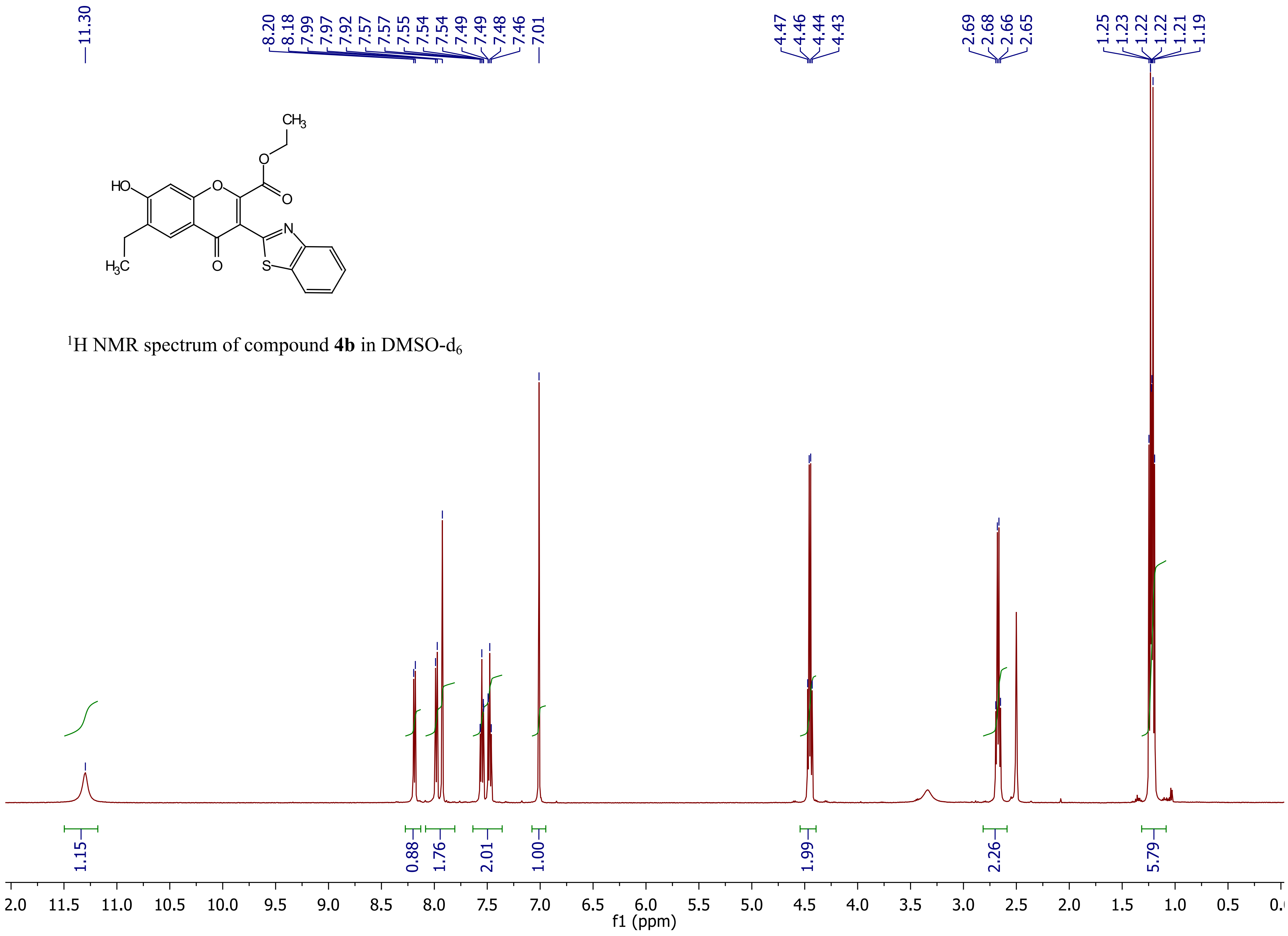

^{1}H NMR spectrum of compound **4b** in DMSO-d_6

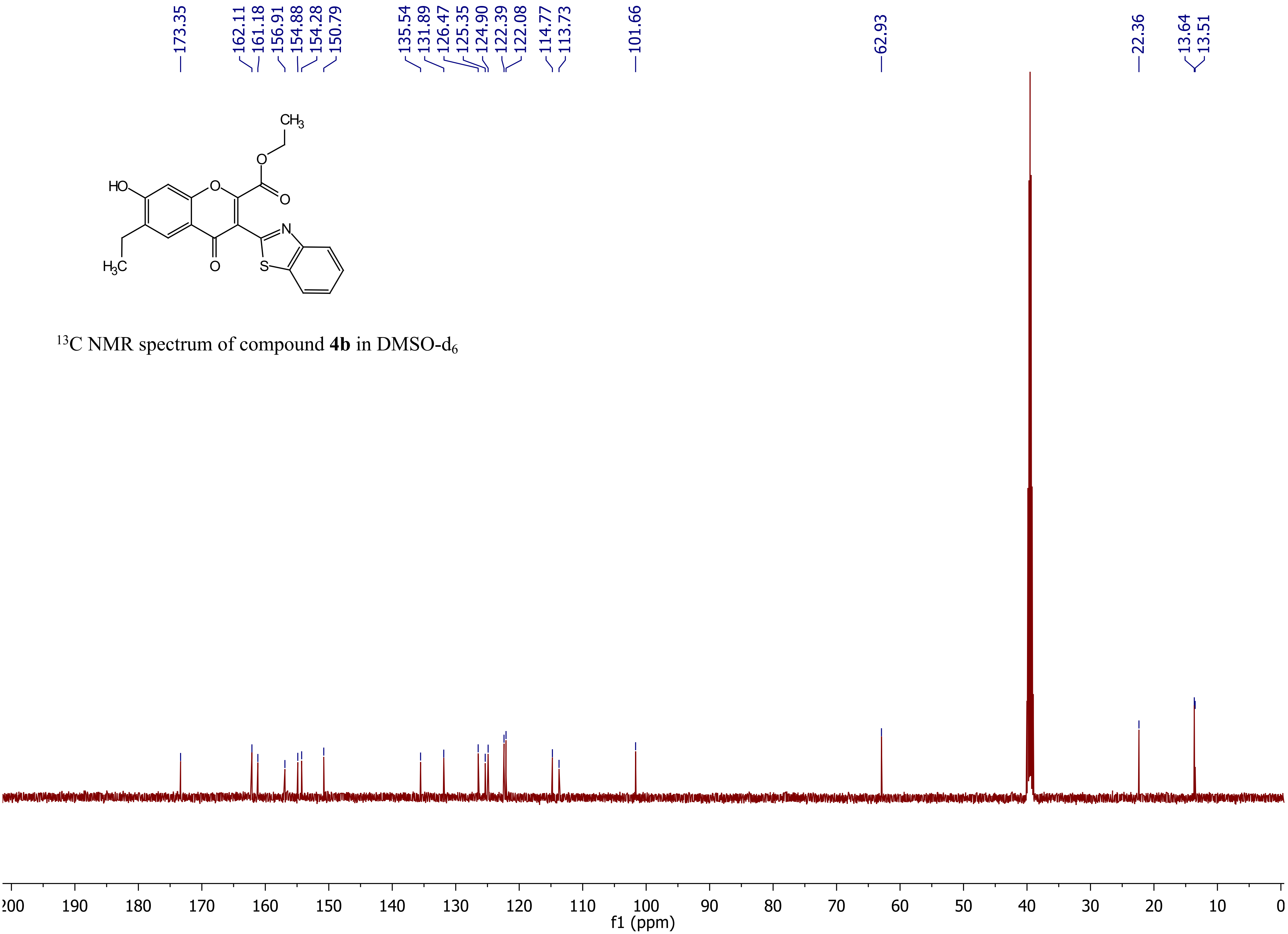

^{13}C NMR spectrum of compound **4b** in DMSO-d_6

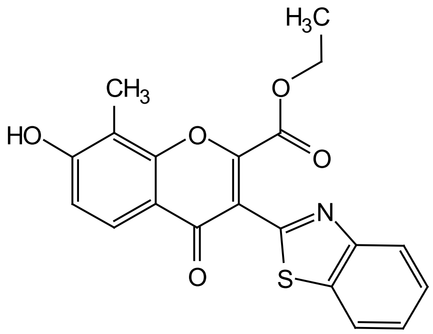

^{1}H NMR spectrum of compound **4c** in DMSO-d_6

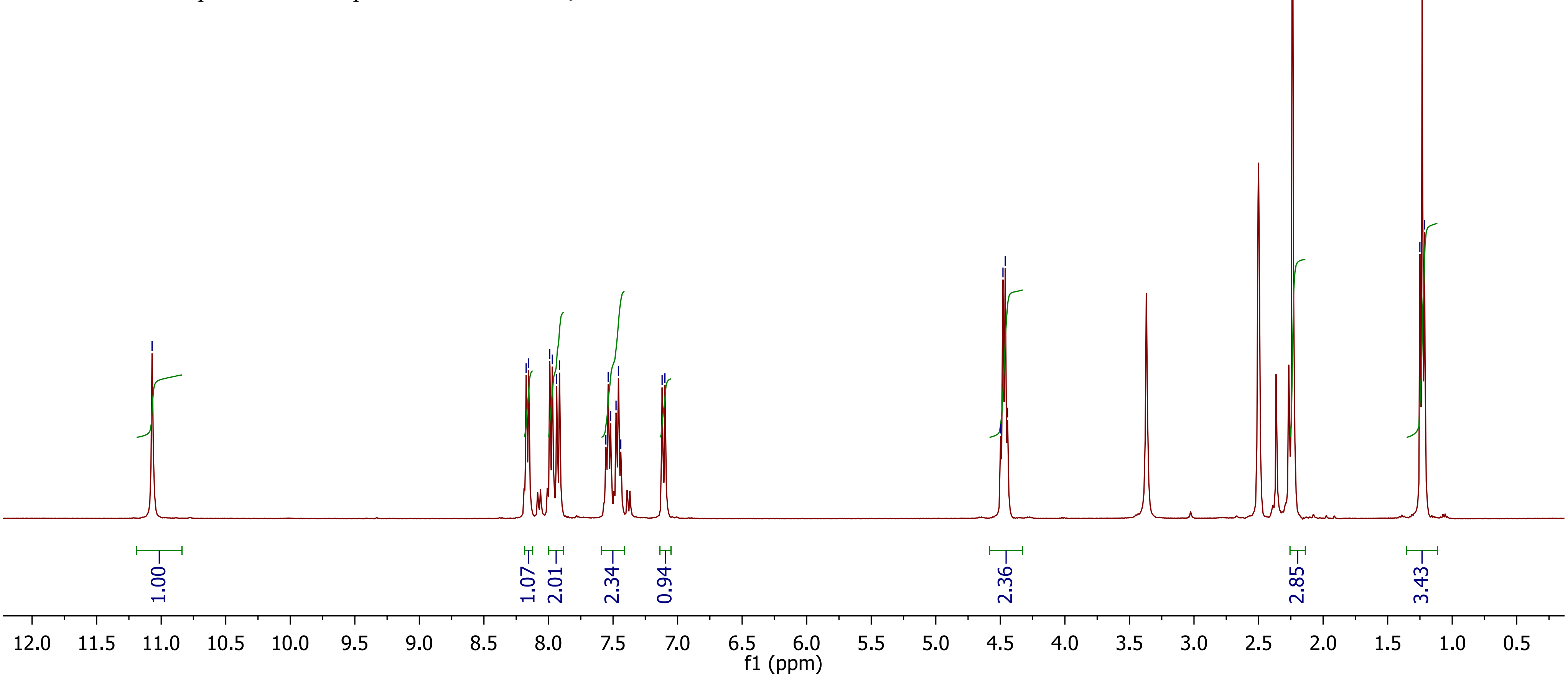

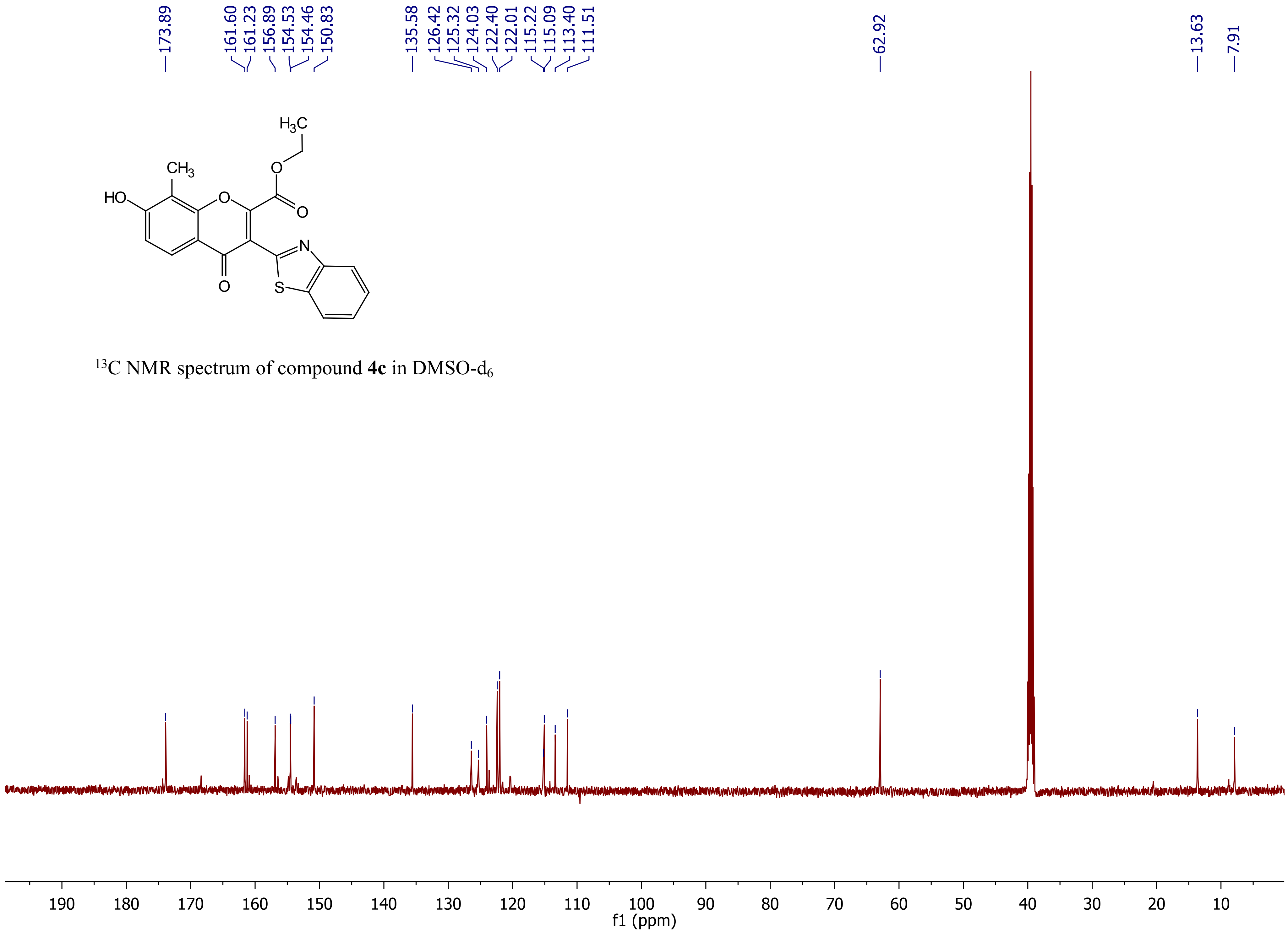

^{13}C NMR spectrum of compound **4c** in DMSO-d_6

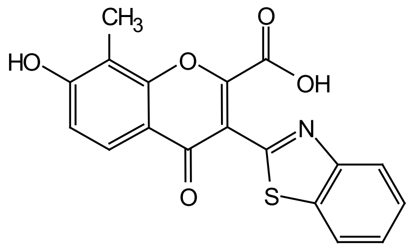

^{1}H NMR spectrum of compound **4d** in DMSO-d_6

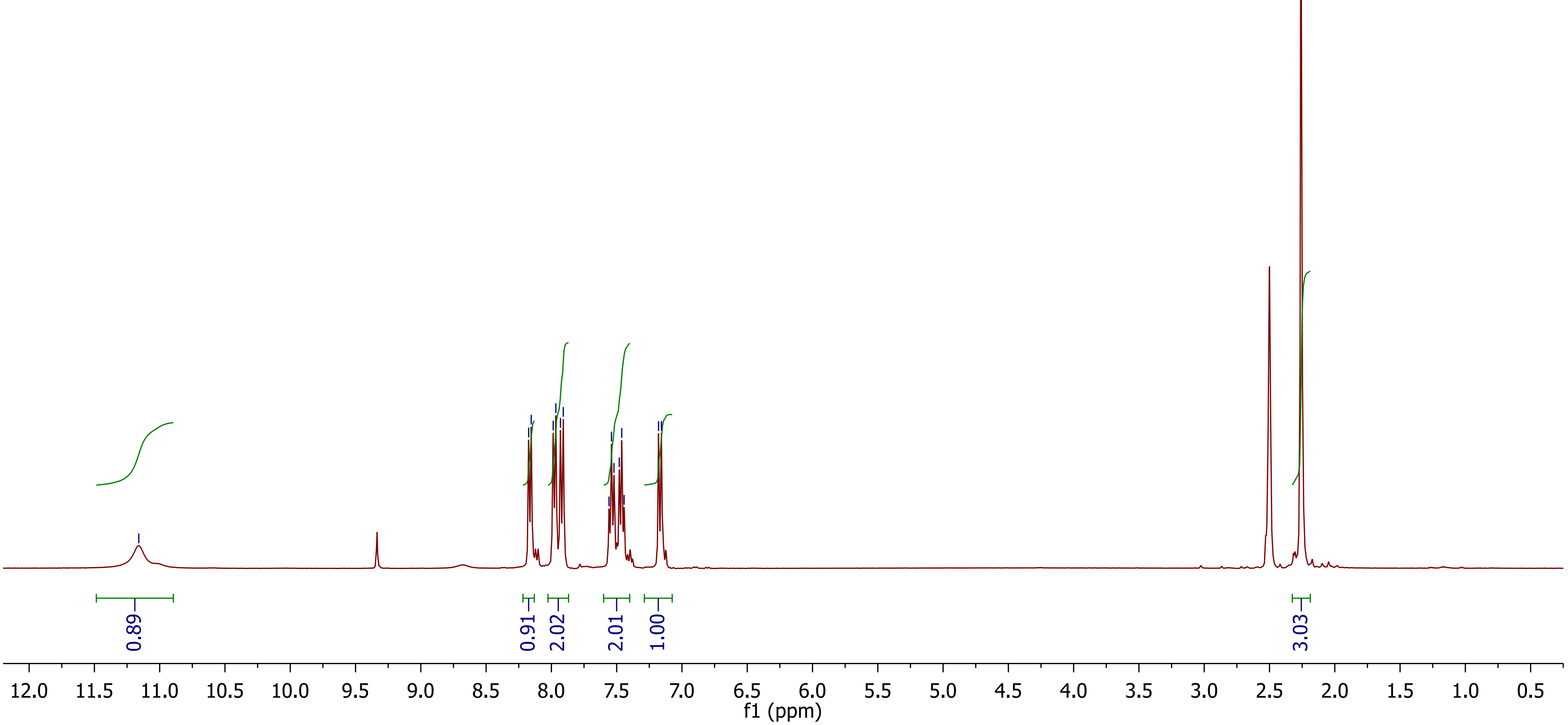

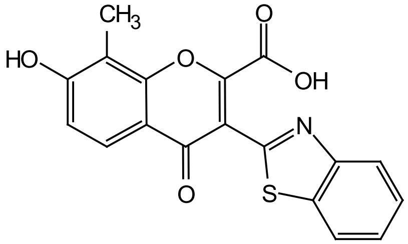

^{13}C NMR spectrum of compound **4d** in DMSO-d_6

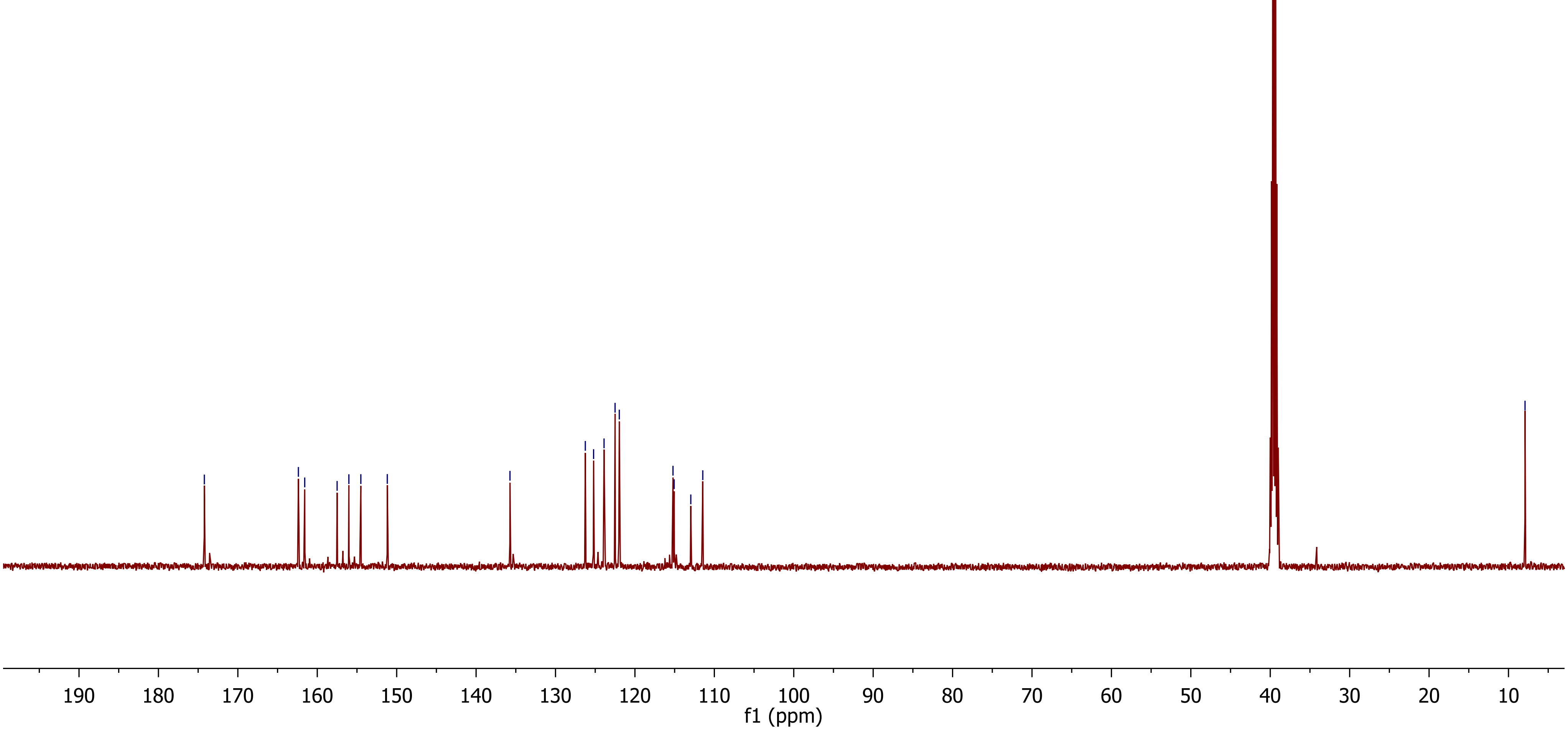

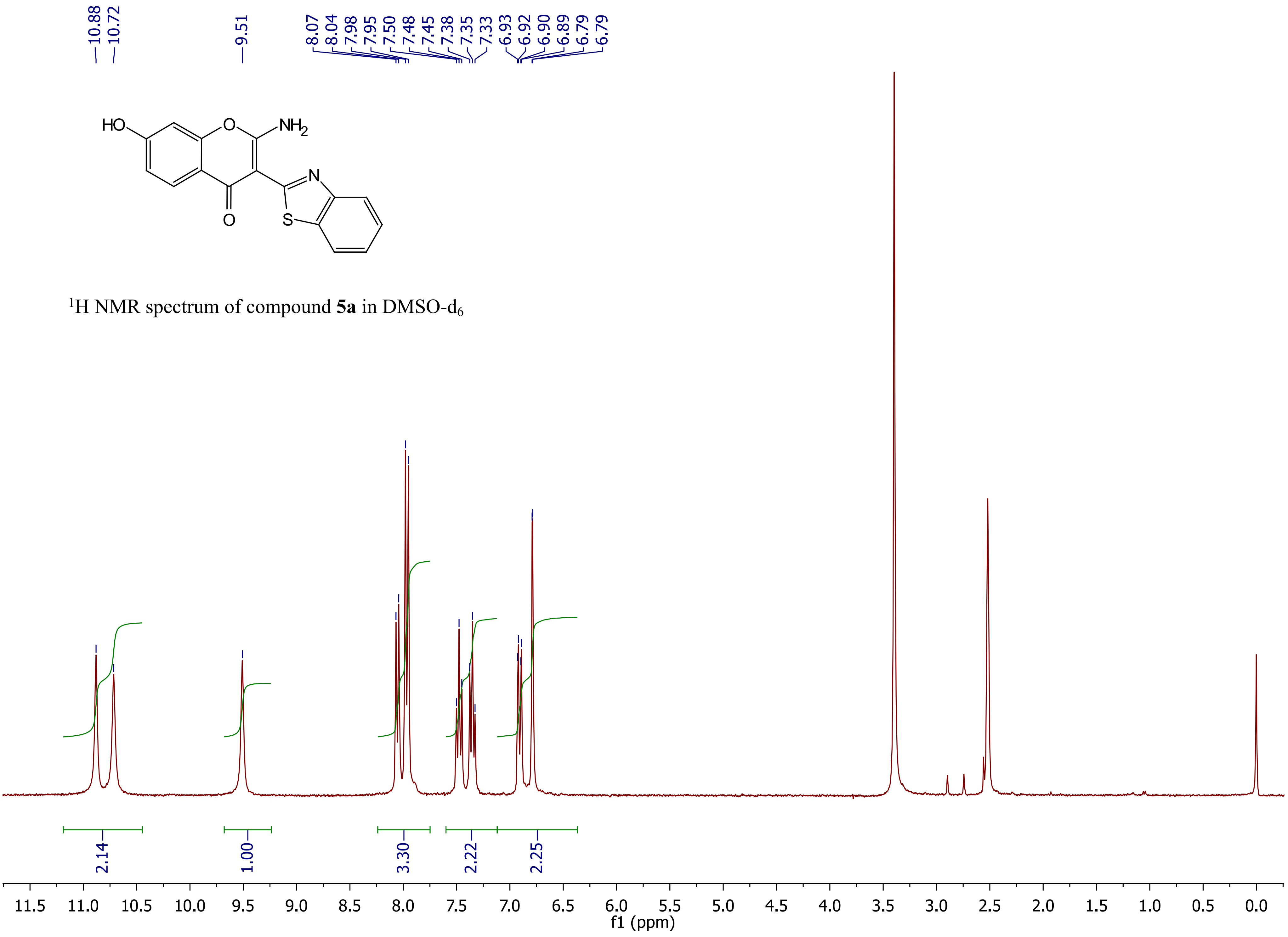

^{1}H NMR spectrum of compound **5a** in DMSO-d_6

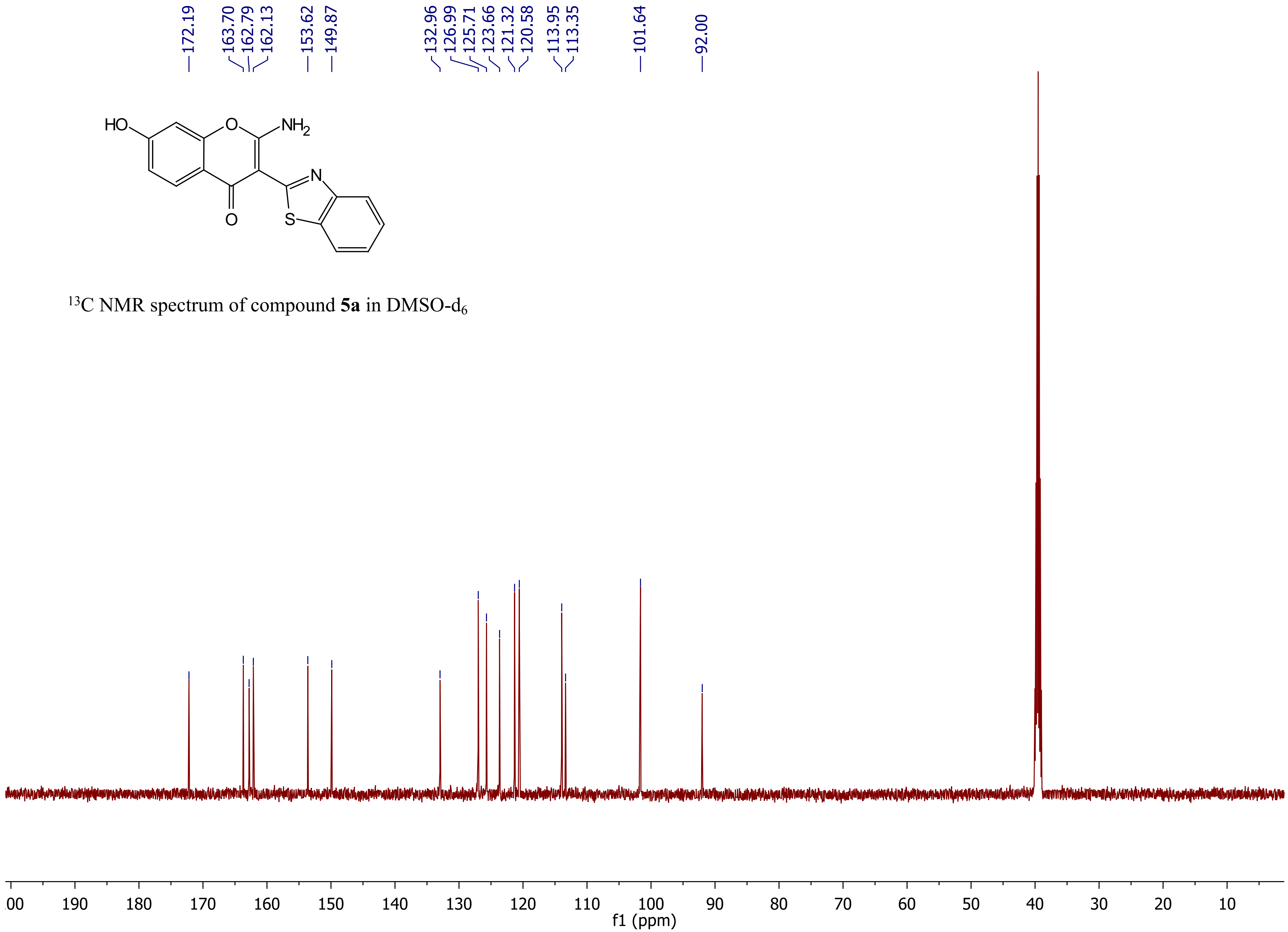

^{13}C NMR spectrum of compound **5a** in DMSO-d_6

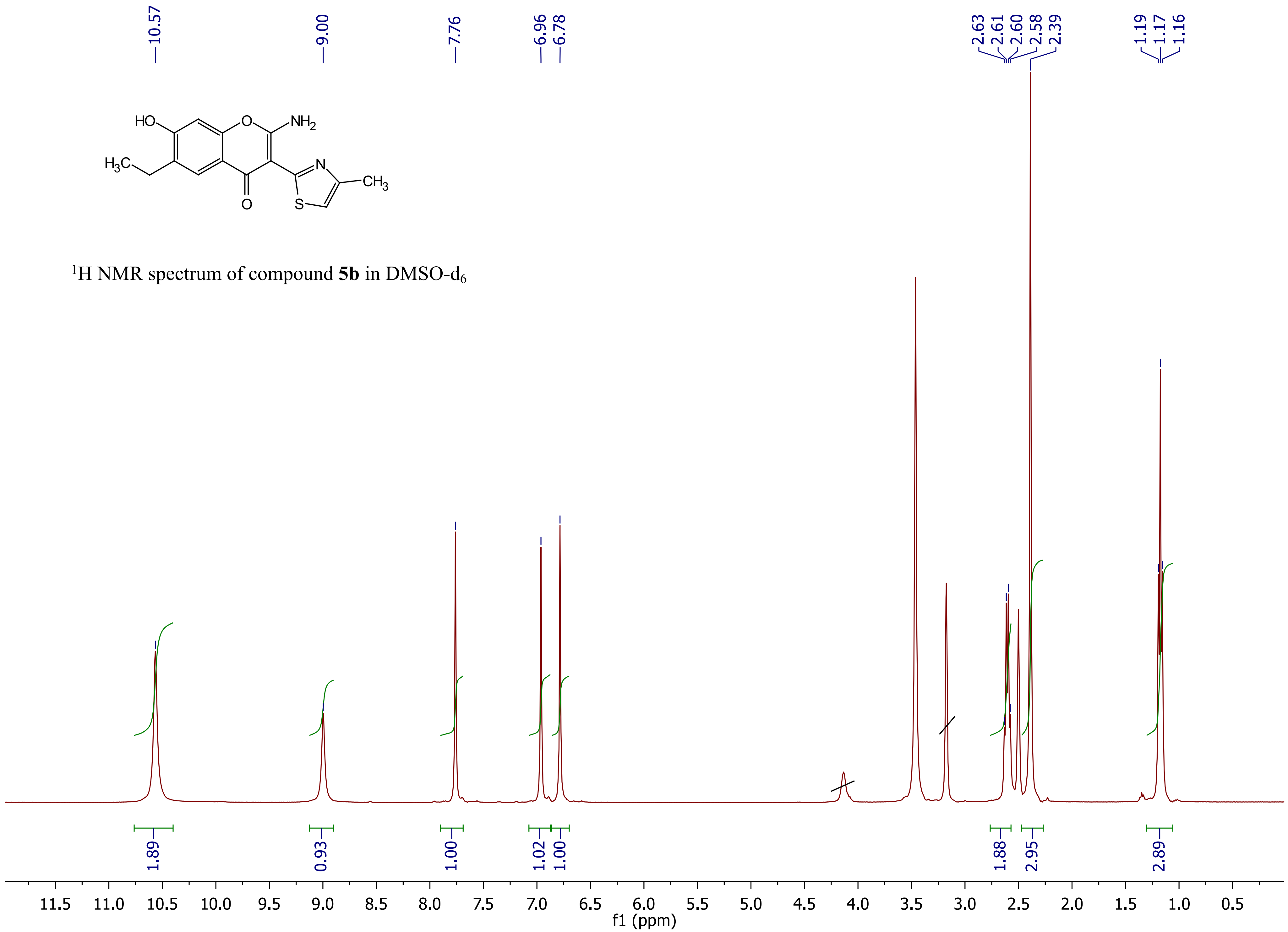

^{1}H NMR spectrum of compound **5b** in DMSO-d_6

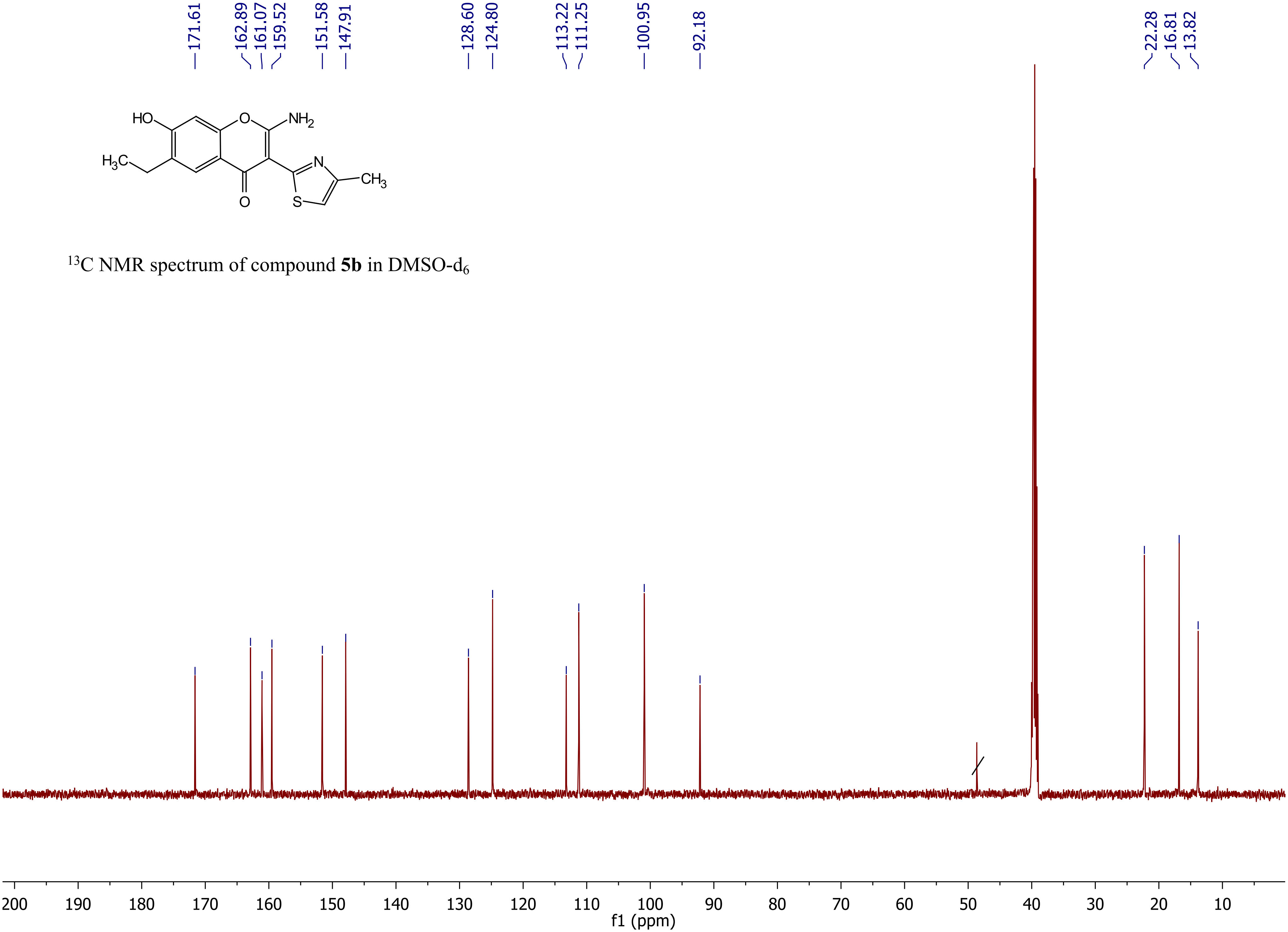

^{13}C NMR spectrum of compound **5b** in DMSO-d_6

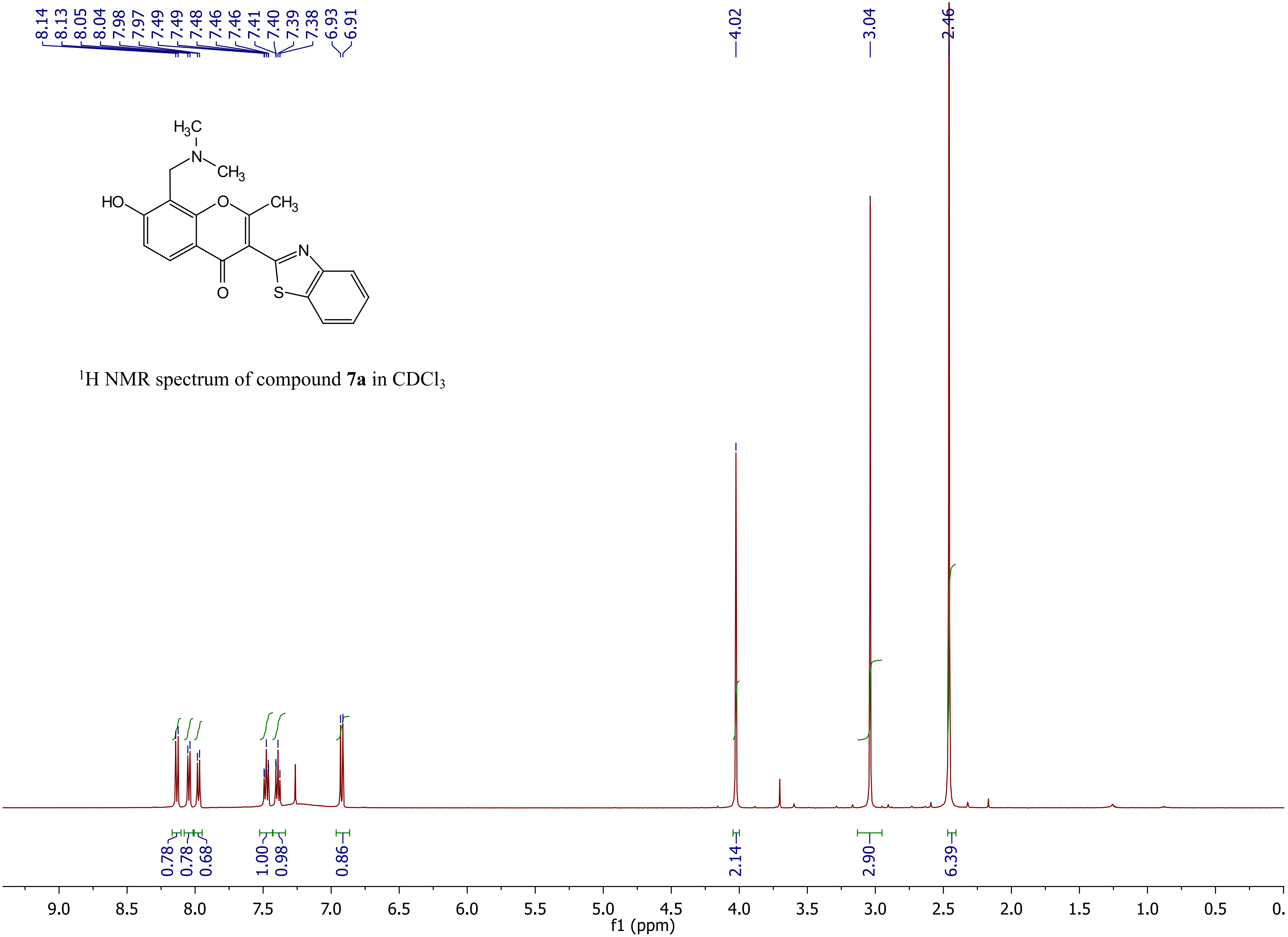

^{1}H NMR spectrum of compound **7a** in $CDCl_3$

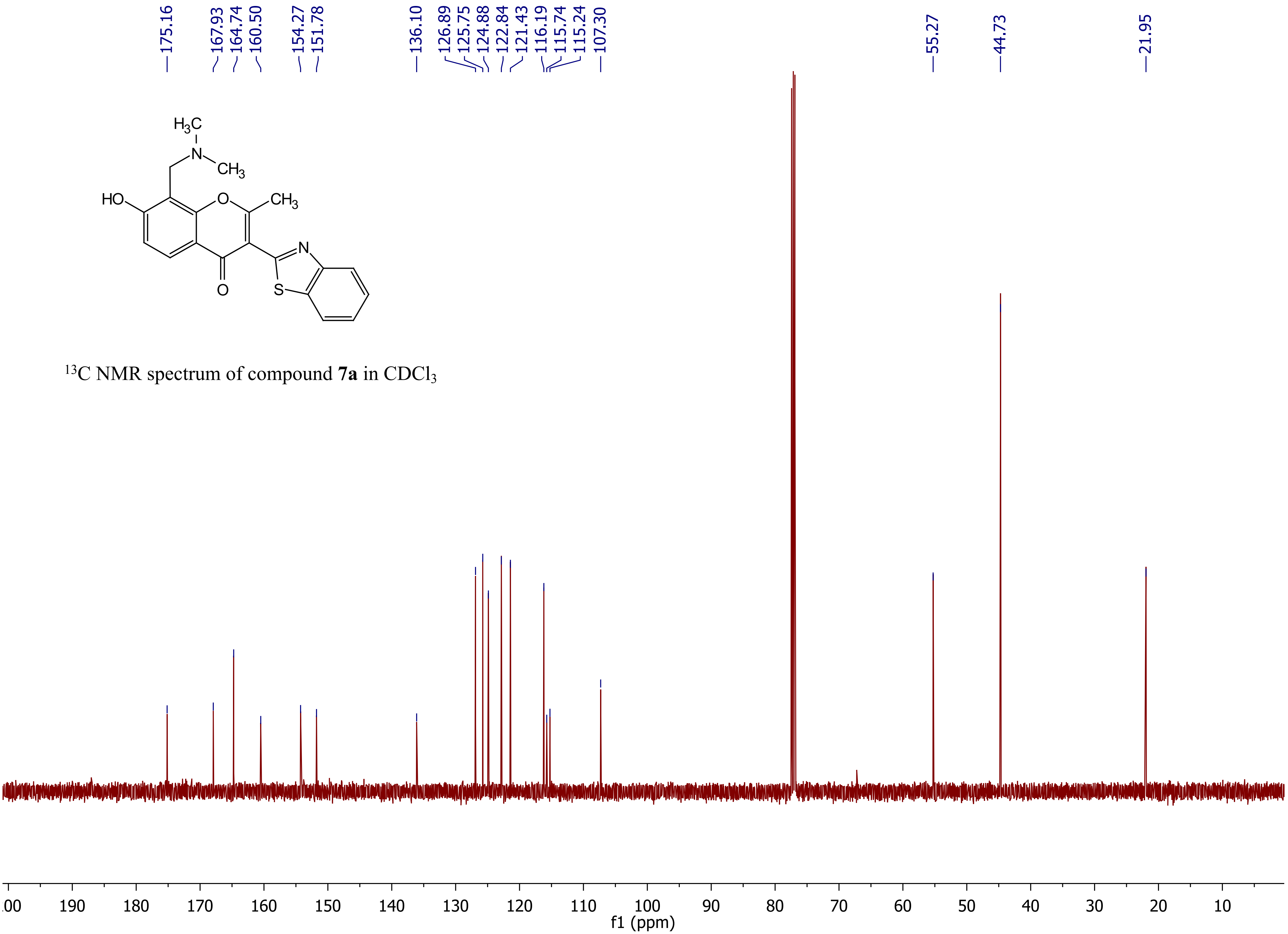

^{13}C NMR spectrum of compound **7a** in $CDCl_3$

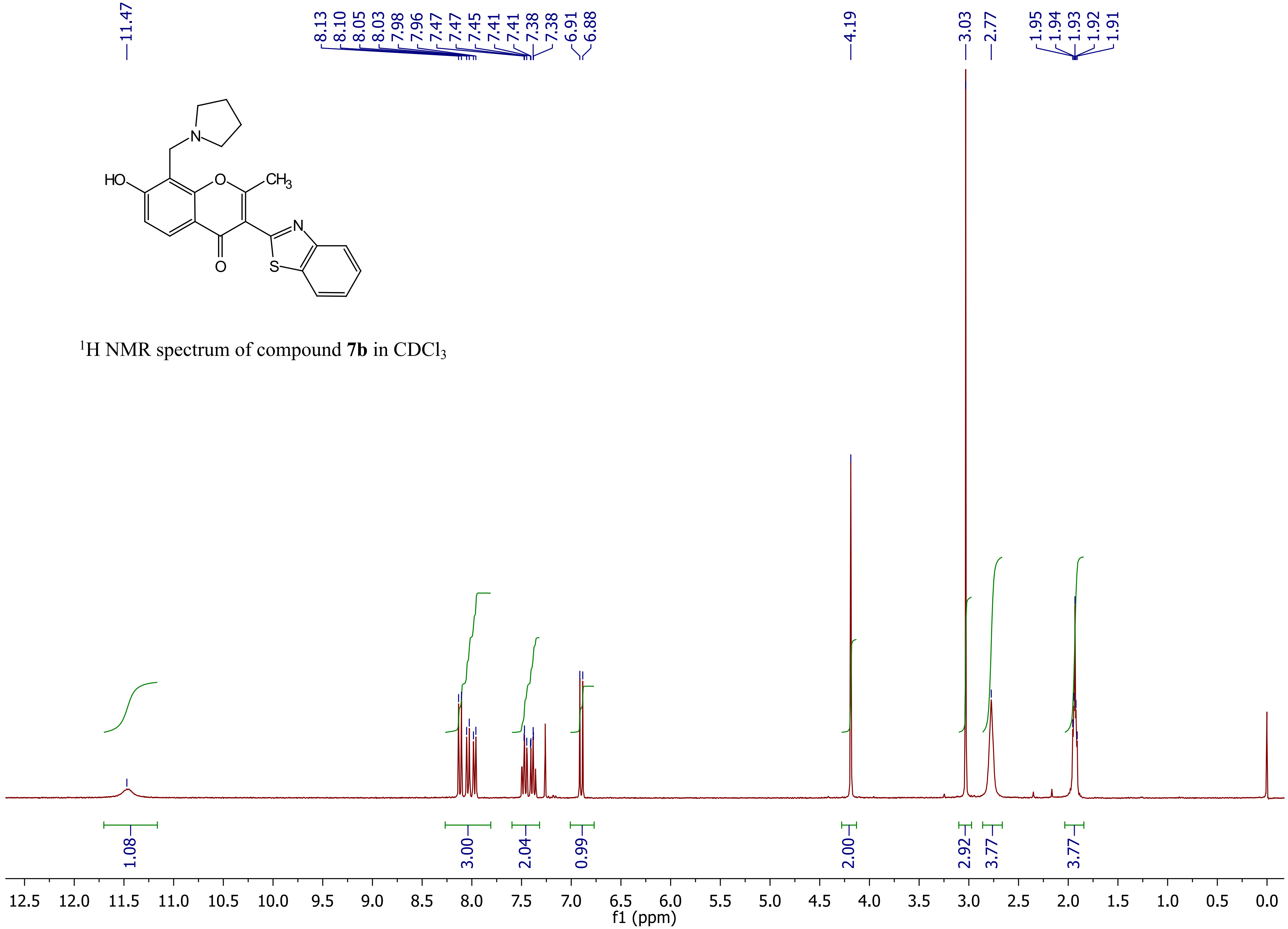

^{1}H NMR spectrum of compound **7b** in $CDCl_3$

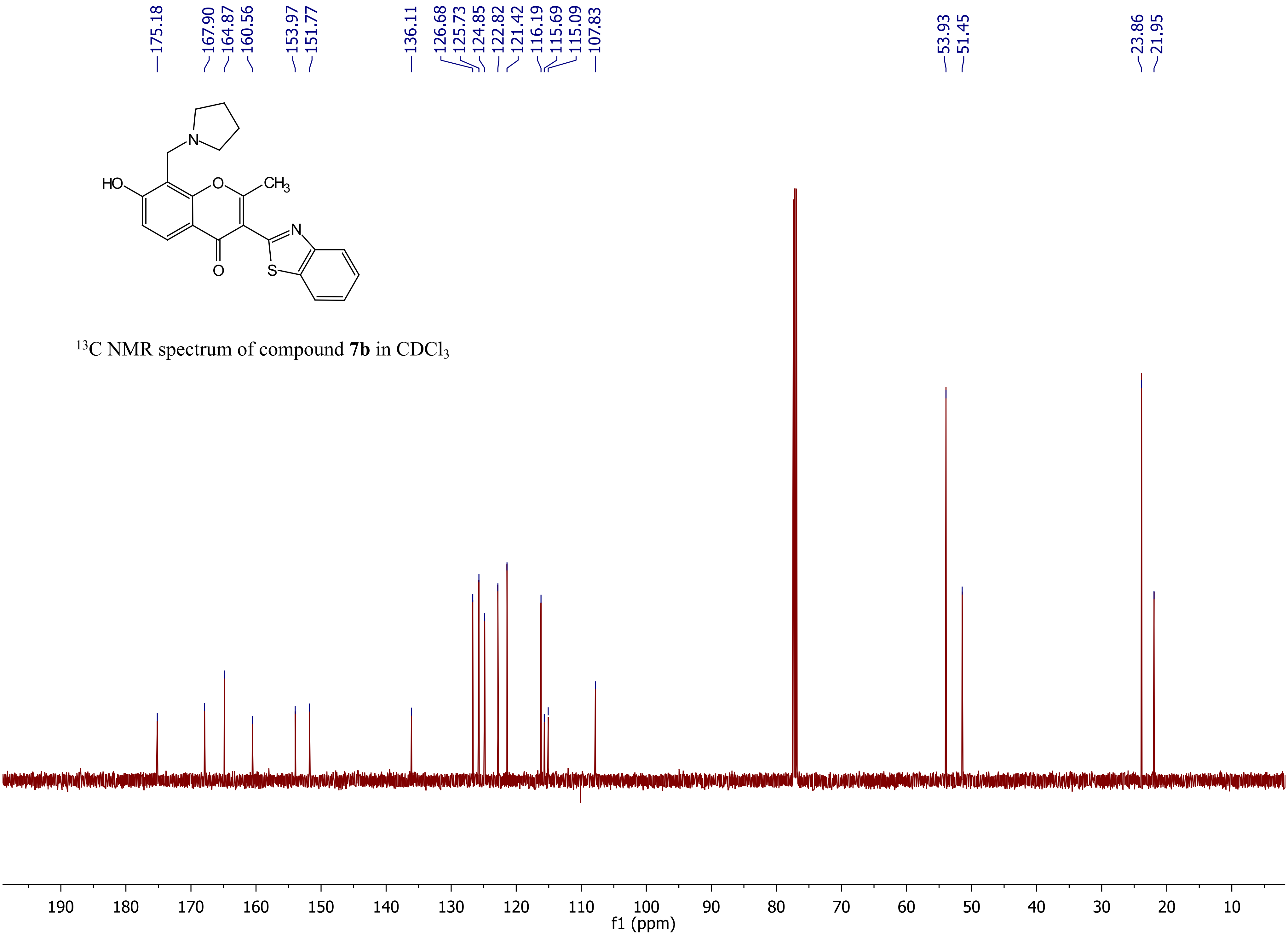

^{13}C NMR spectrum of compound **7b** in $CDCl_3$

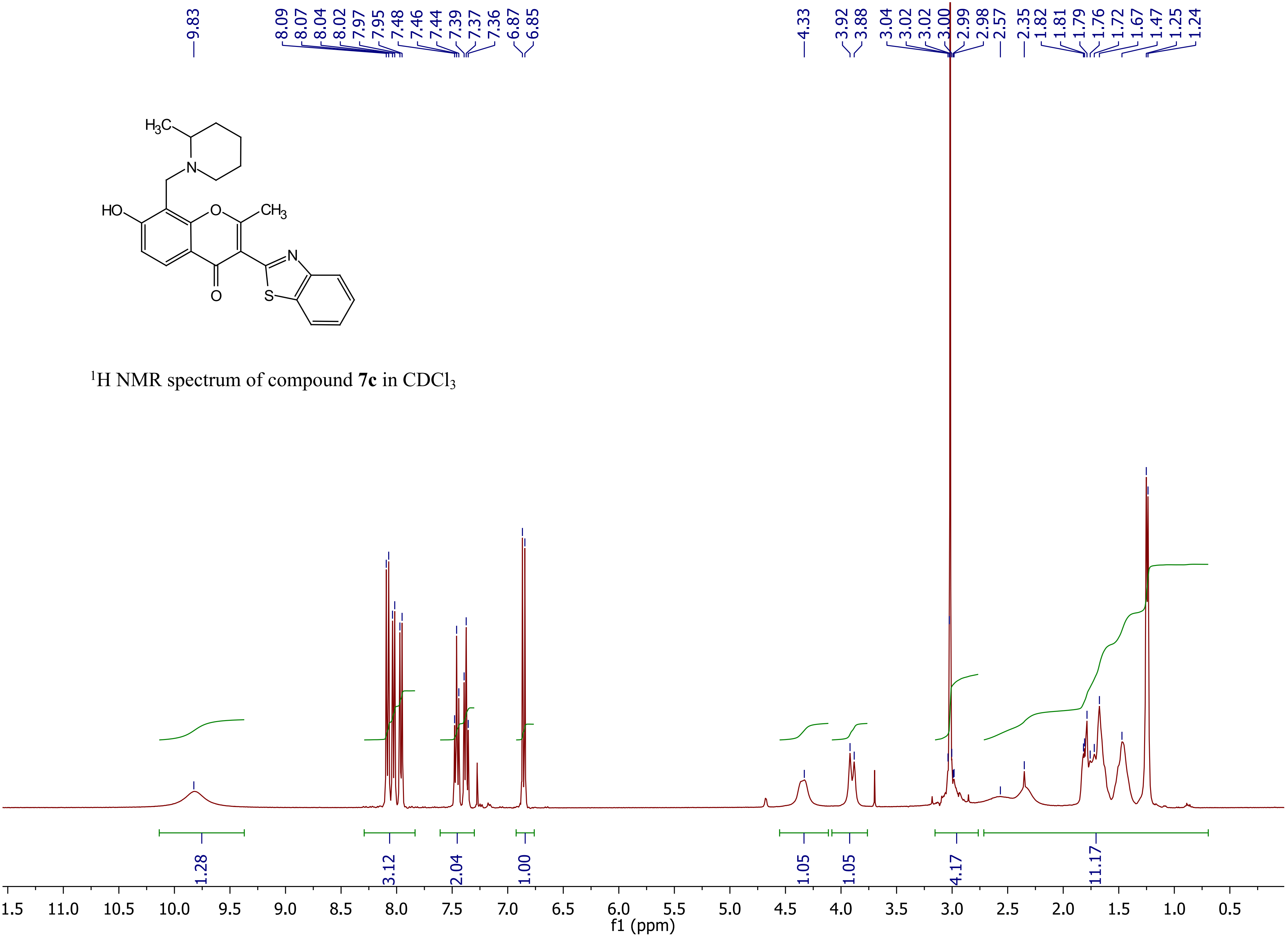

^{1}H NMR spectrum of compound **7c** in $CDCl_3$

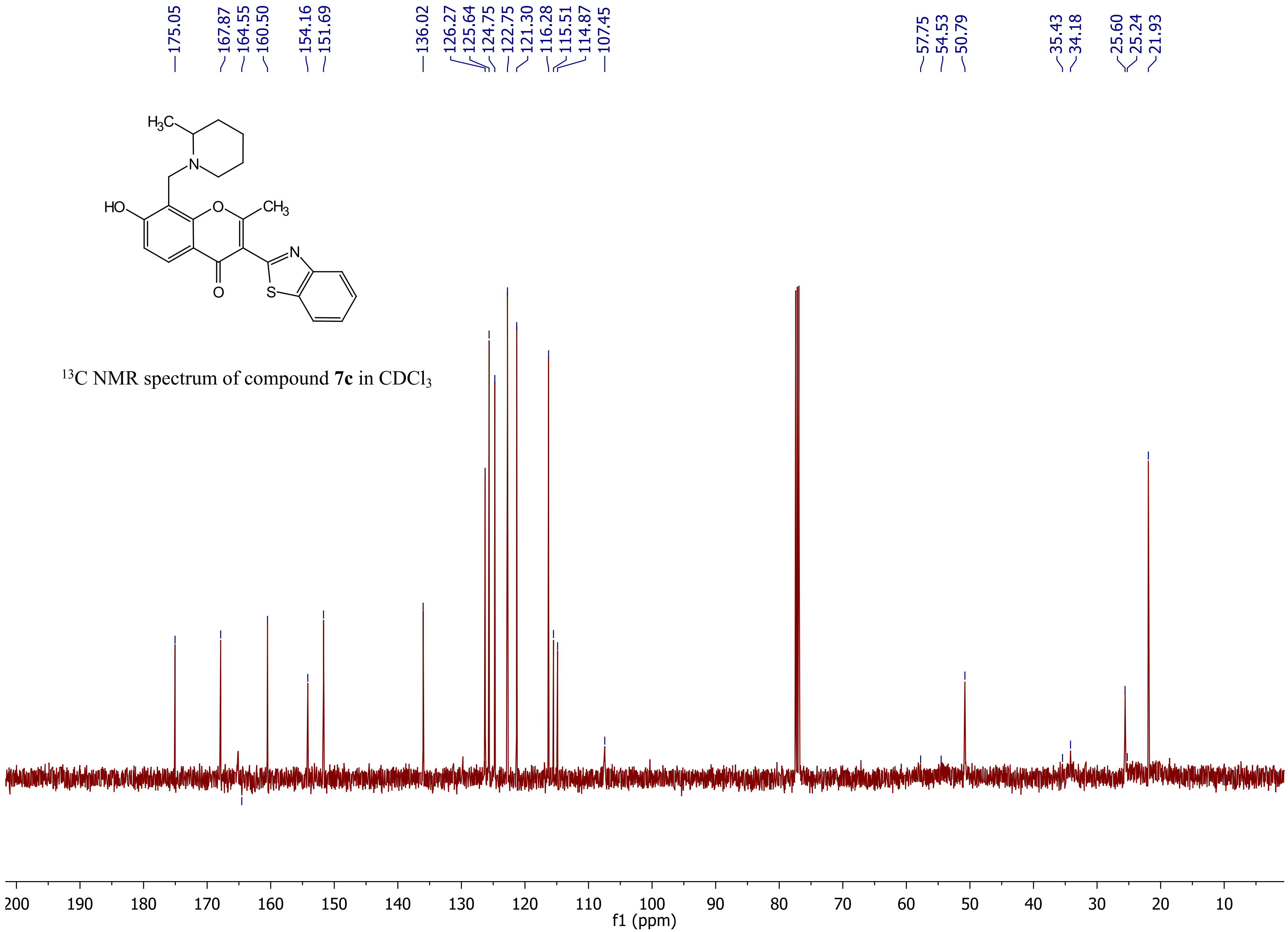

^{13}C NMR spectrum of compound **7c** in $CDCl_3$

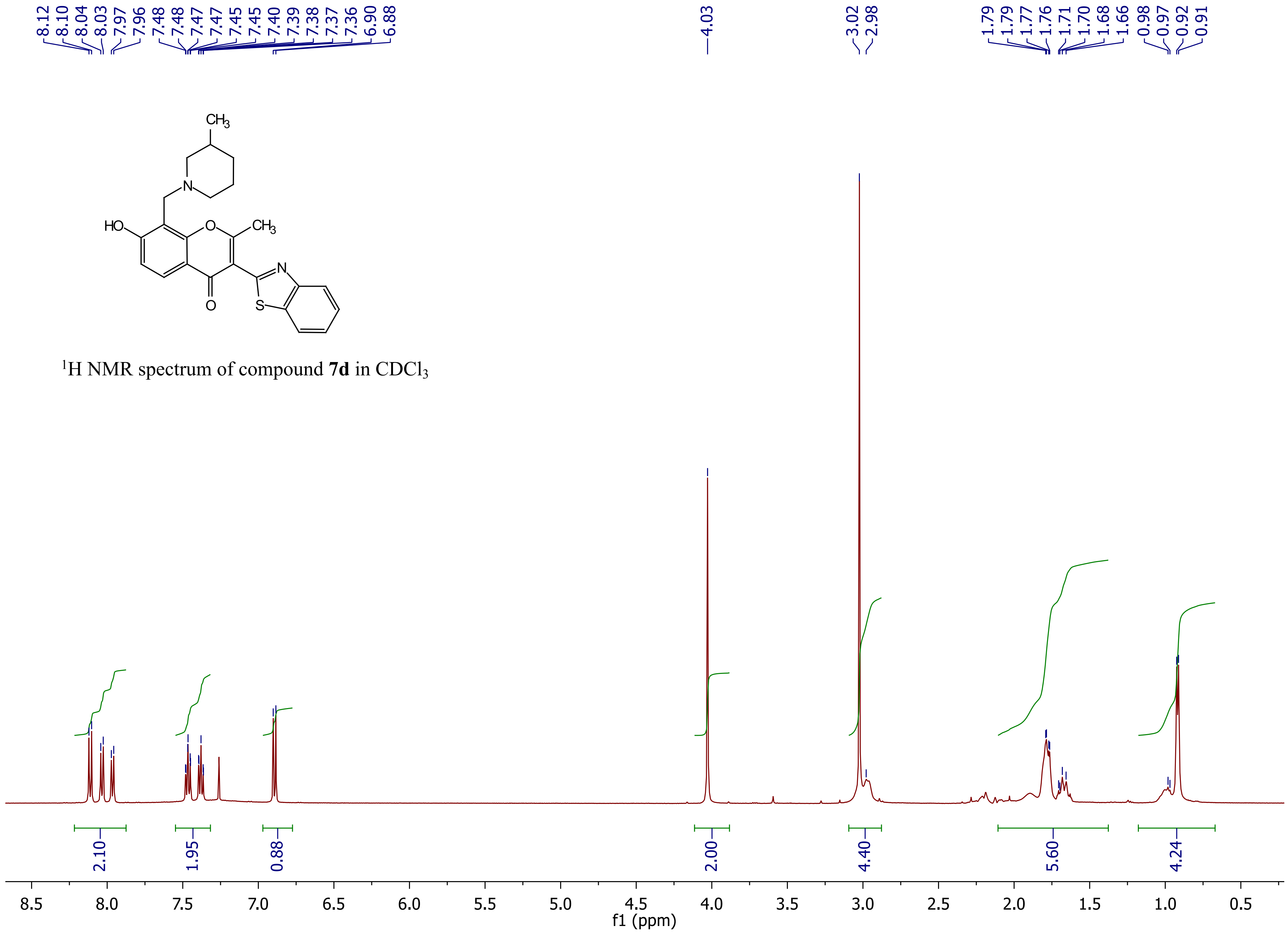

^{1}H NMR spectrum of compound **7d** in $CDCl_3$

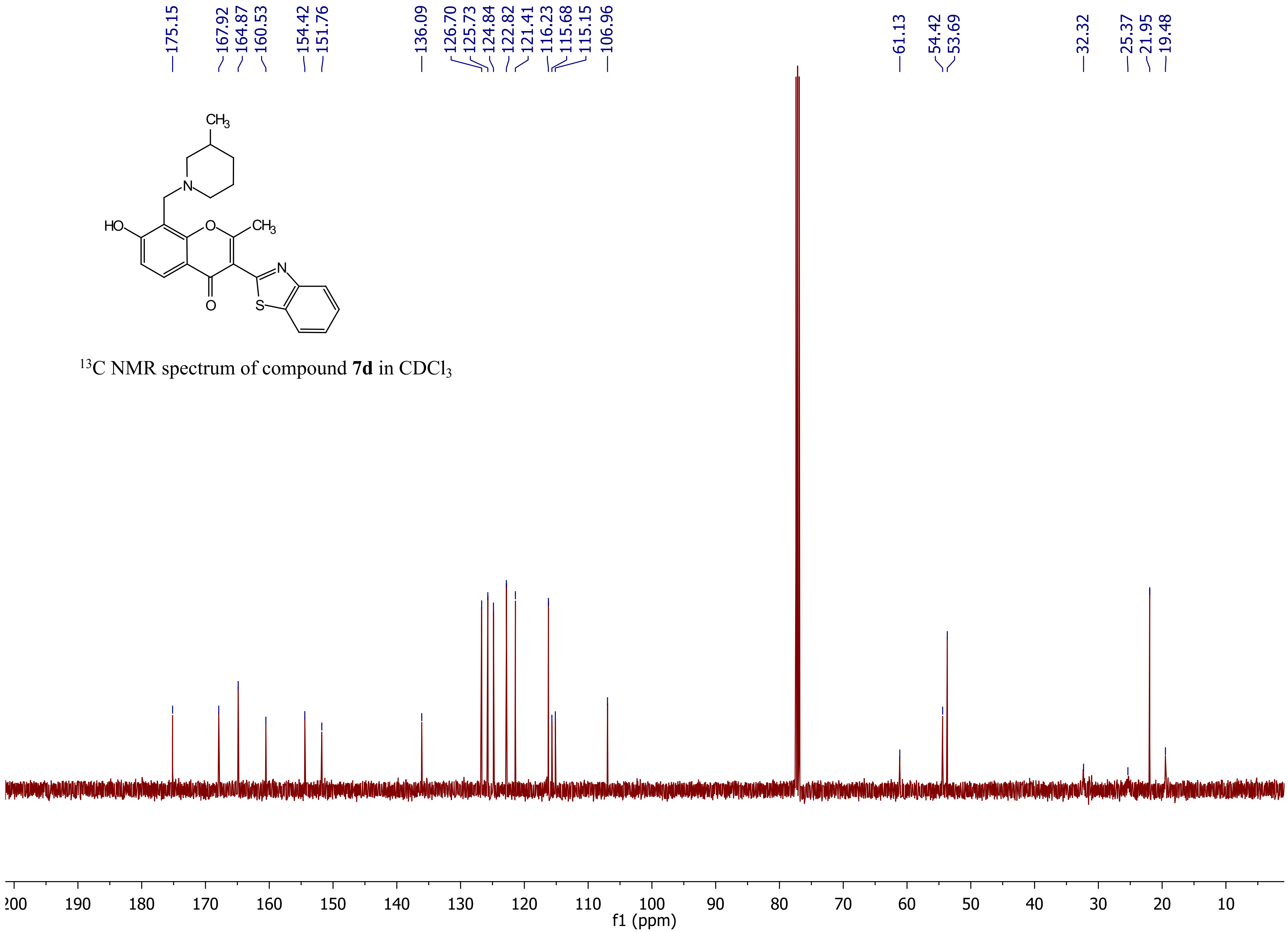

^{13}C NMR spectrum of compound **7d** in $CDCl_3$

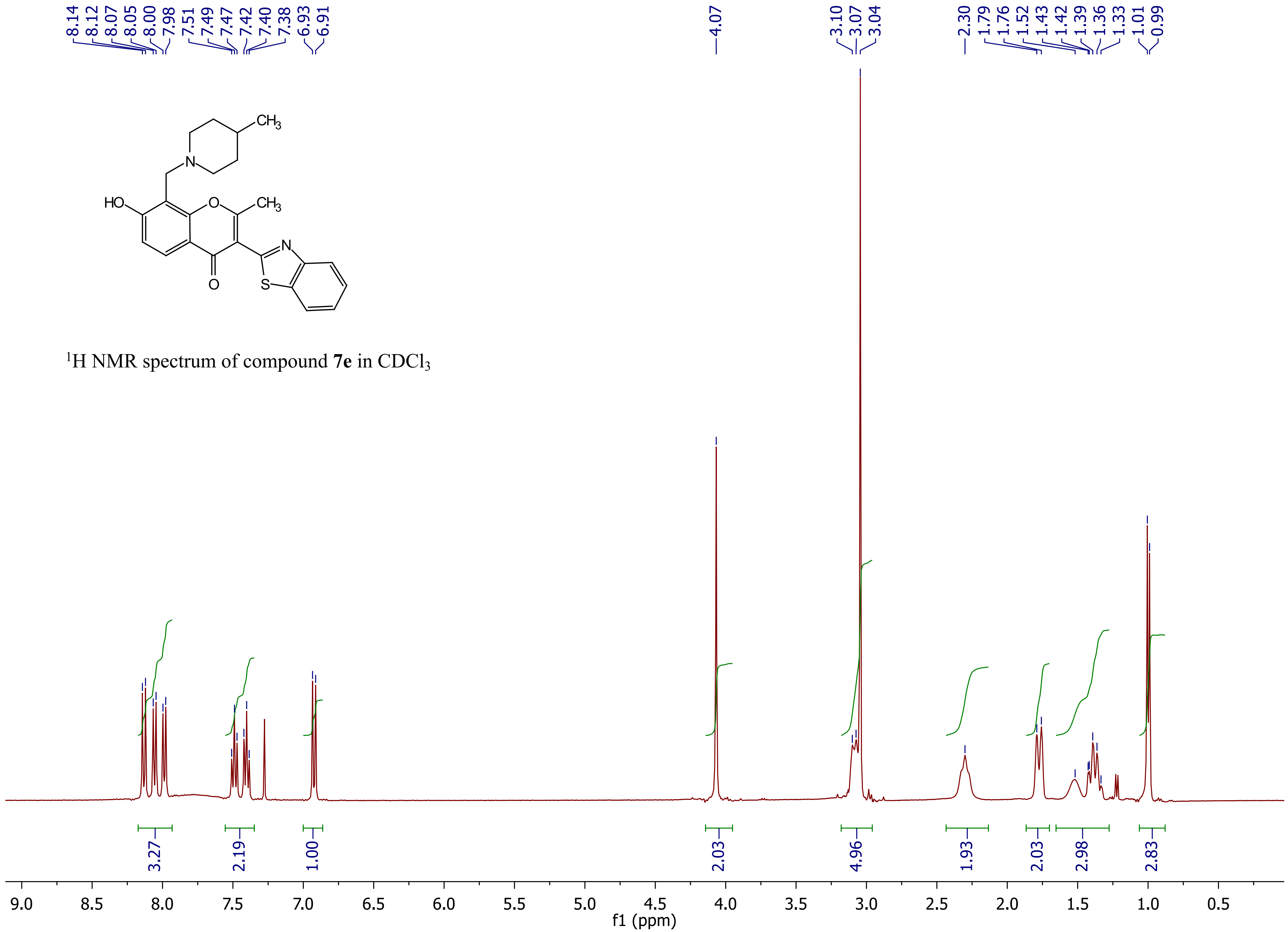

^{1}H NMR spectrum of compound **7e** in $CDCl_3$

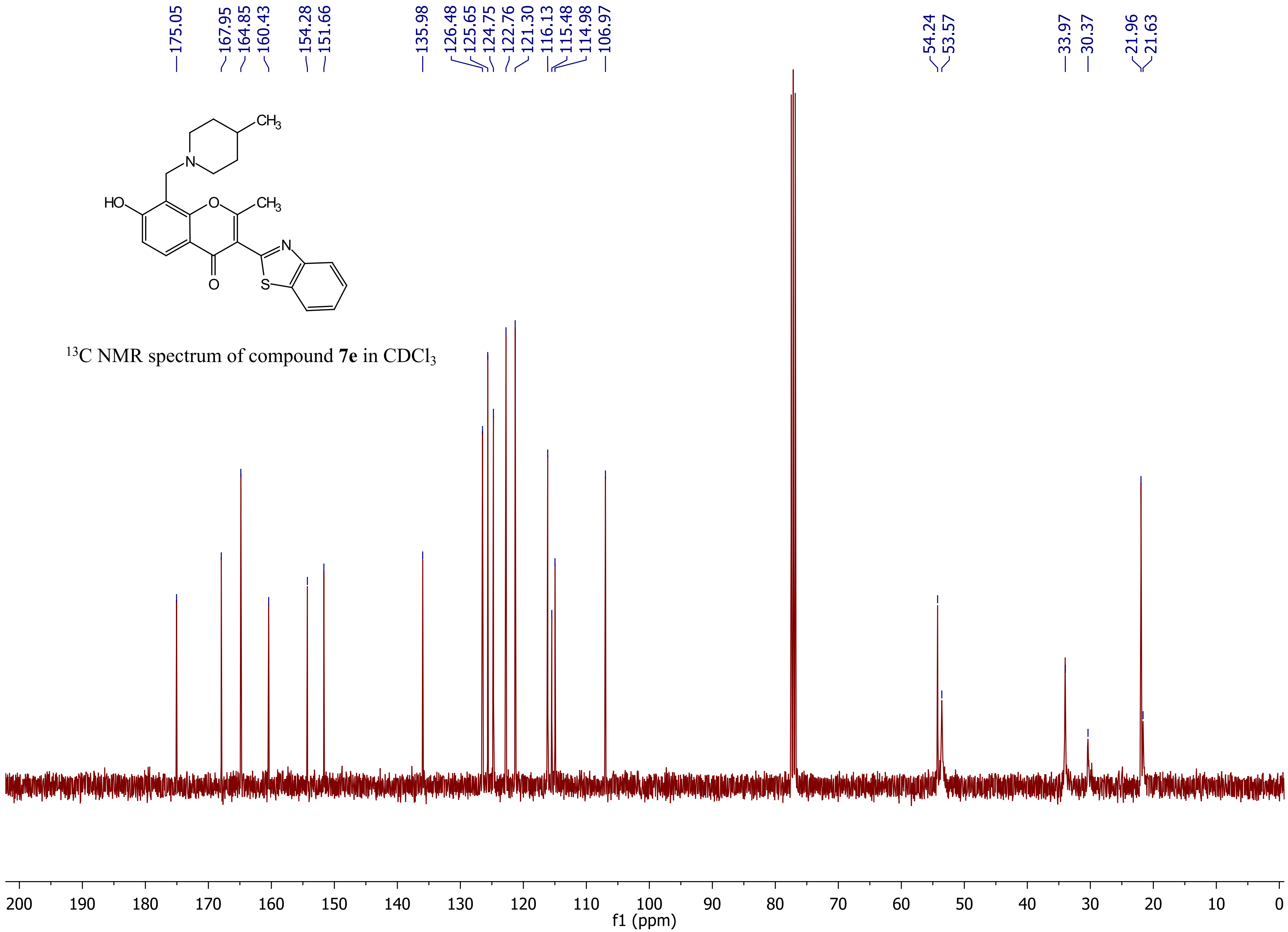

^{13}C NMR spectrum of compound **7e** in $CDCl_3$

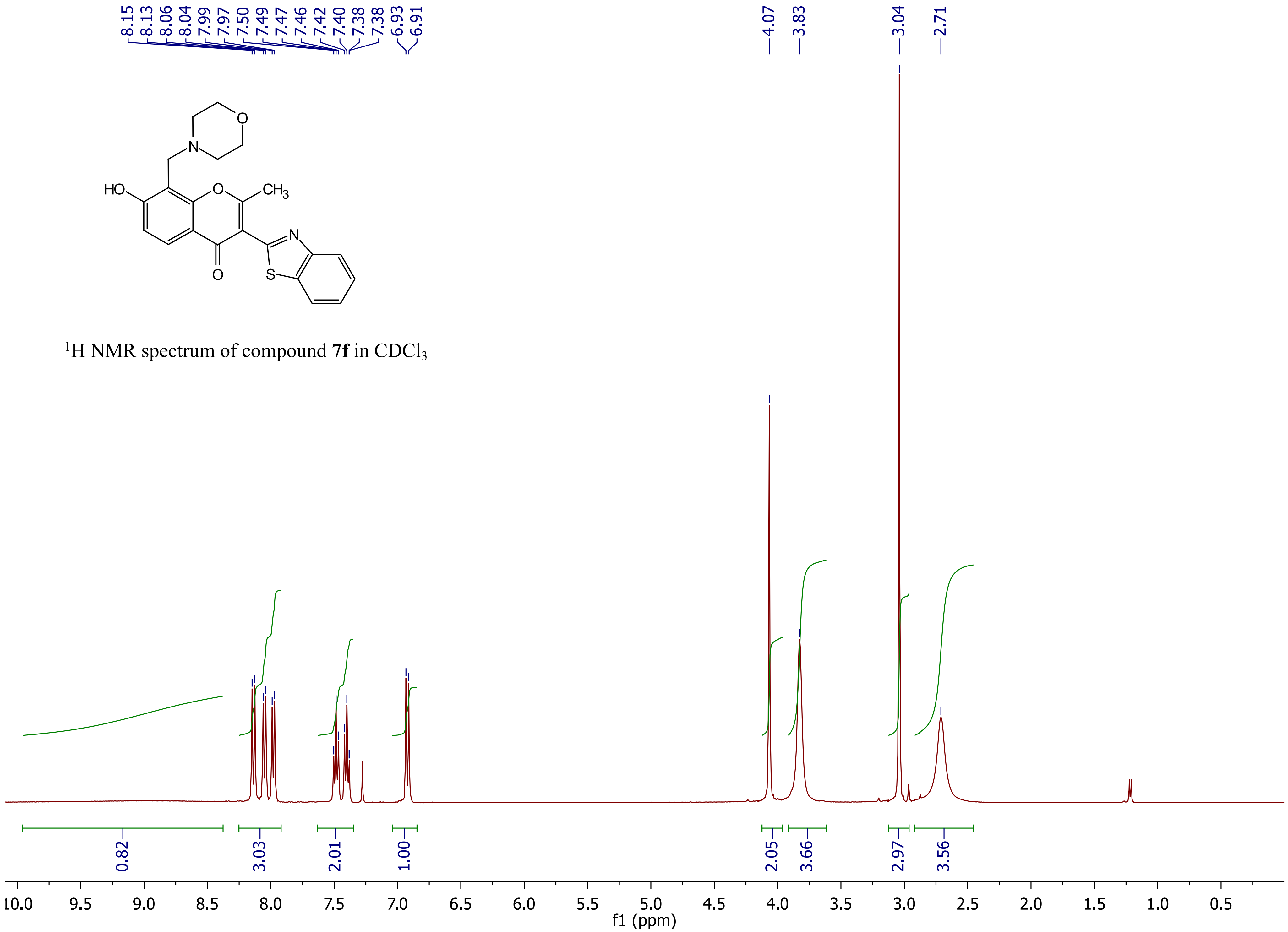

^{1}H NMR spectrum of compound **7f** in $CDCl_3$

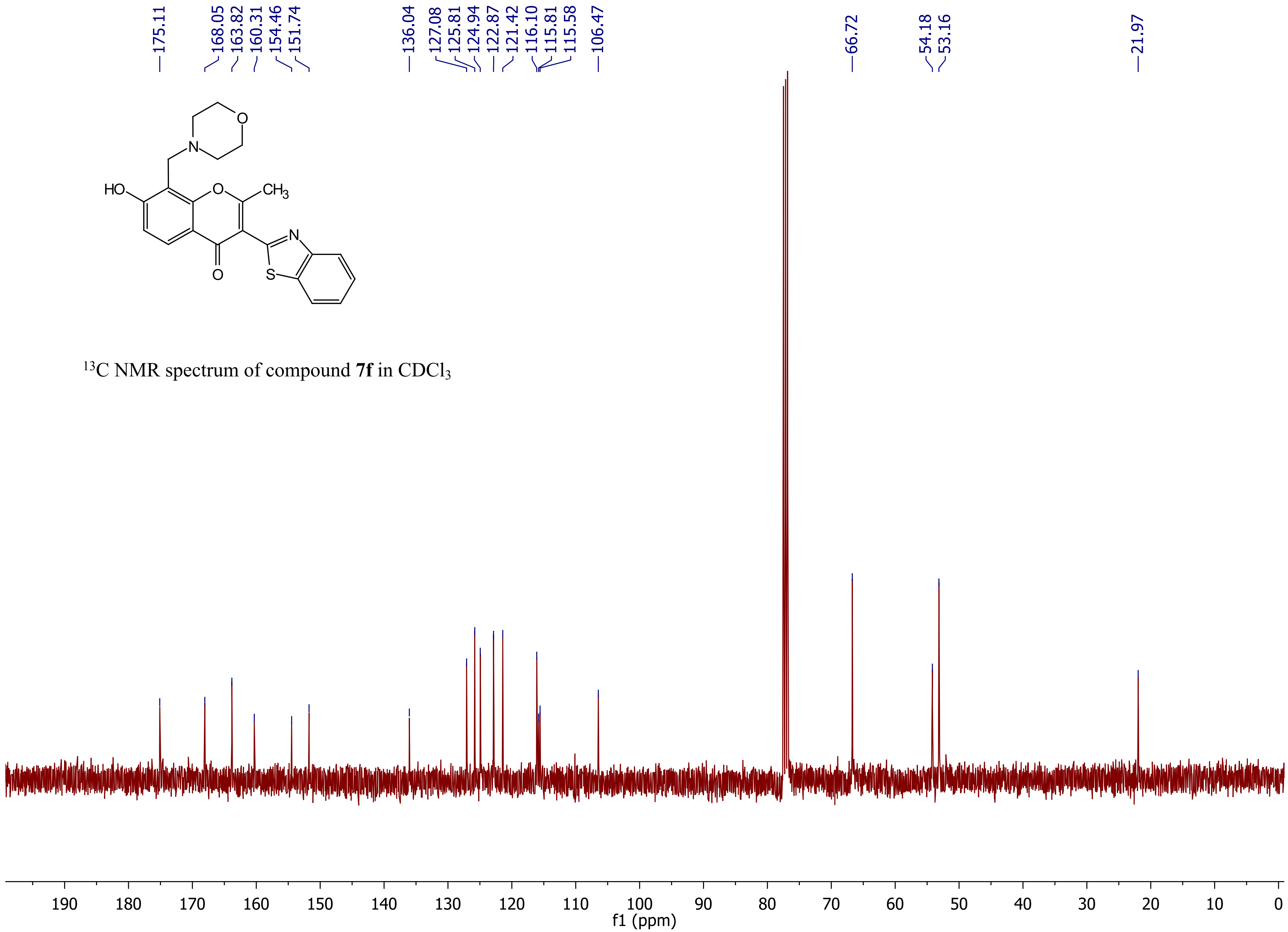

^{13}C NMR spectrum of compound **7f** in $CDCl_3$

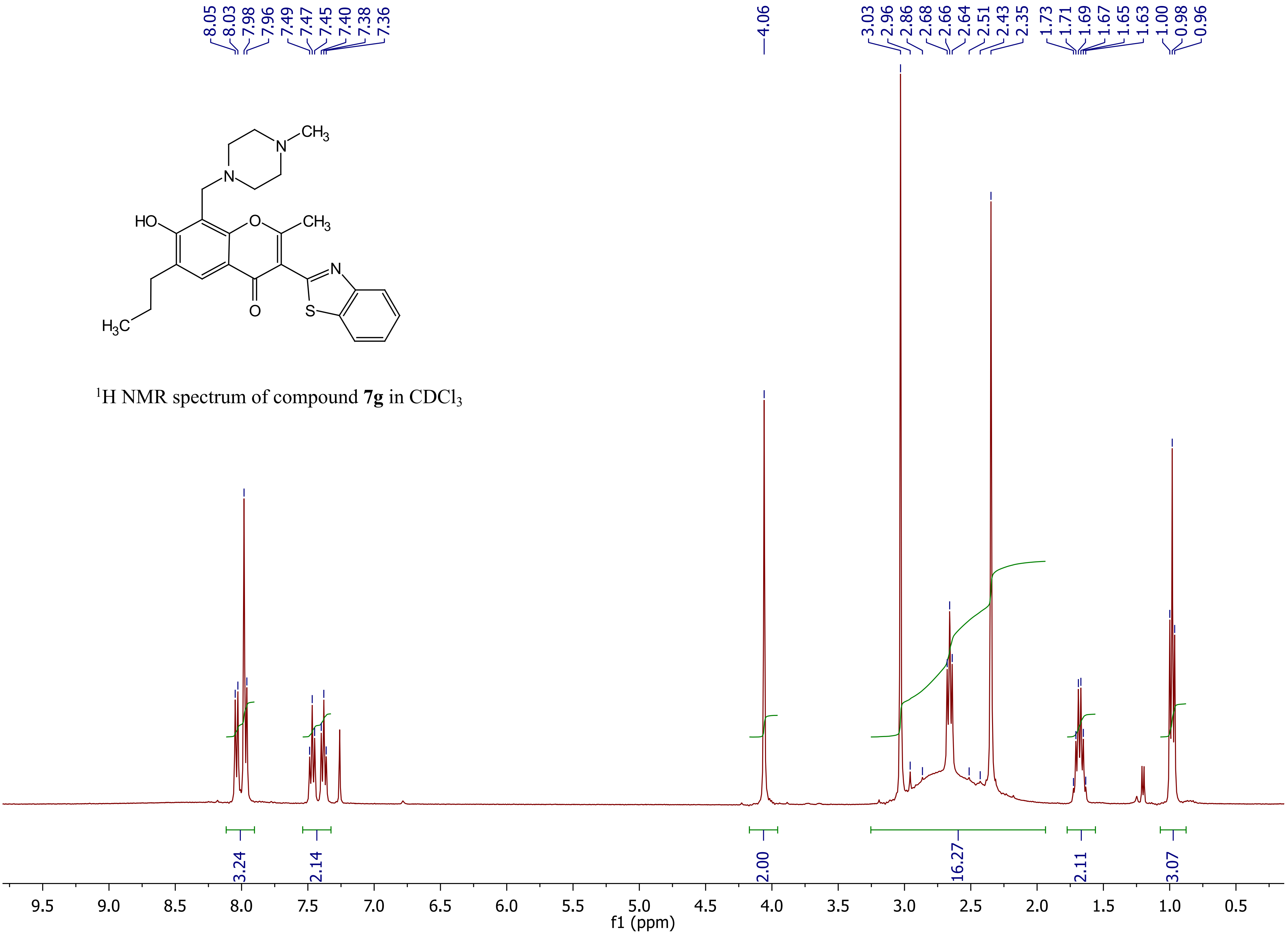

^{1}H NMR spectrum of compound **7g** in $CDCl_3$

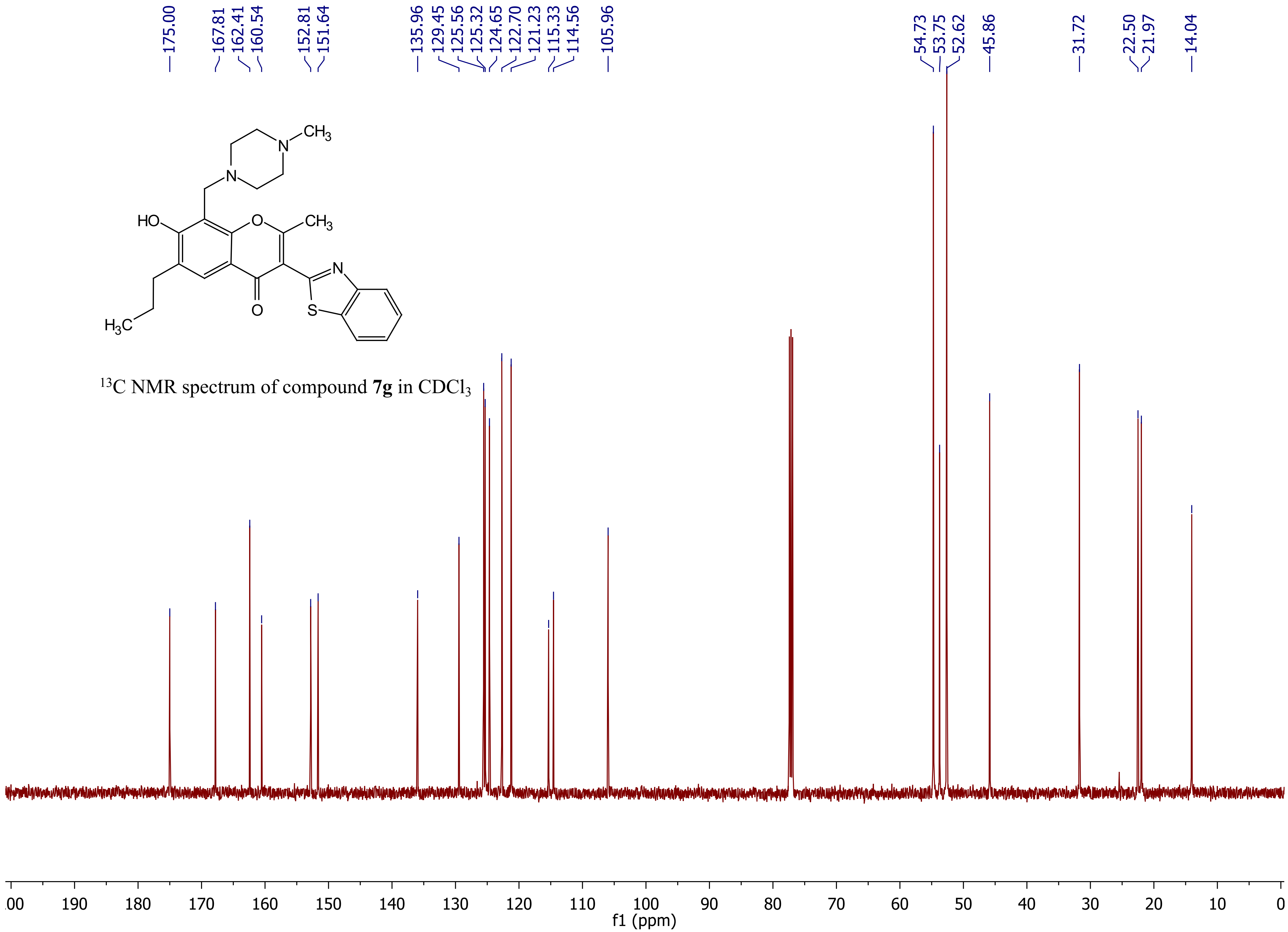

^{13}C NMR spectrum of compound **7g** in $CDCl_3$

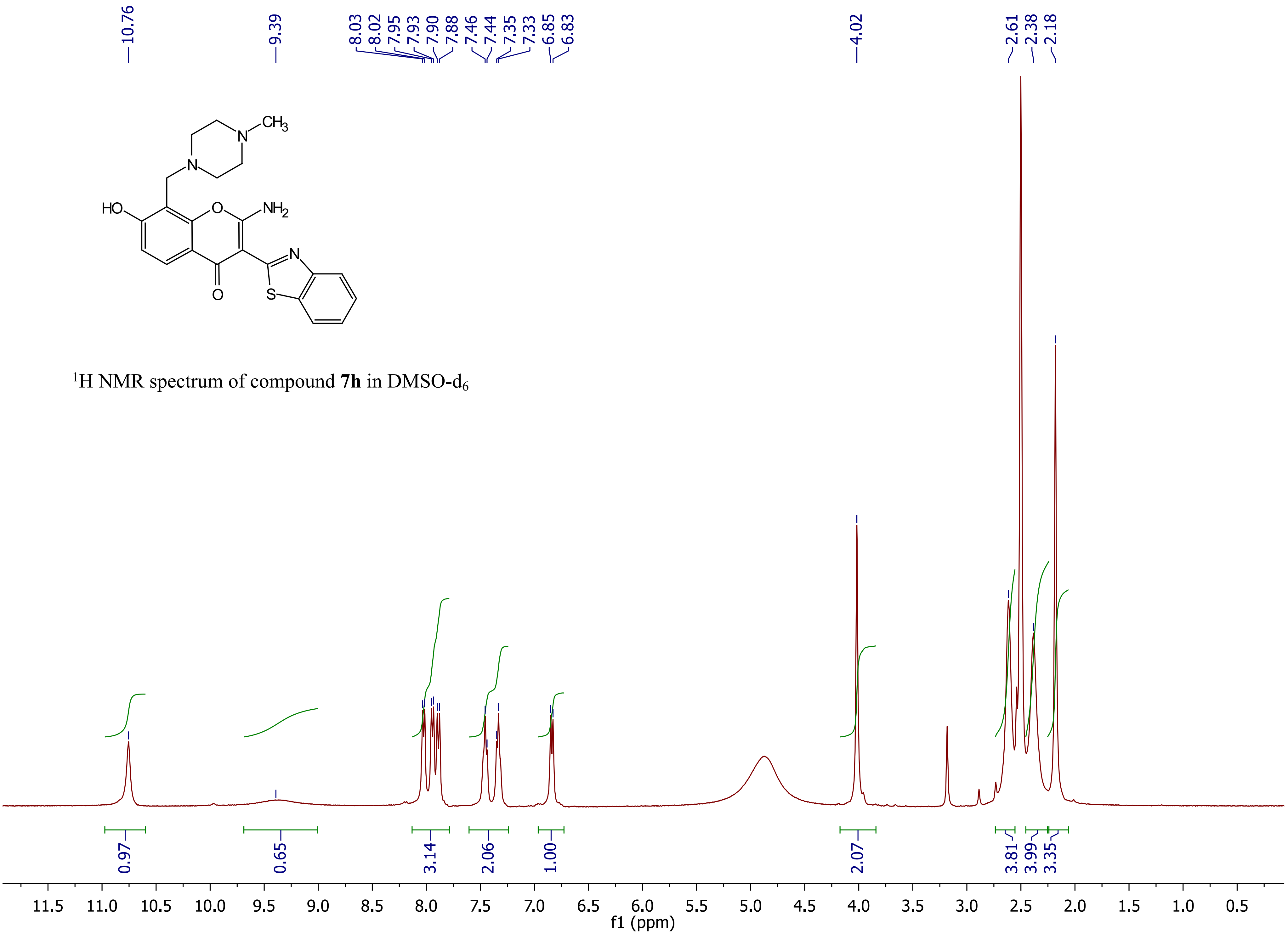

^{1}H NMR spectrum of compound **7h** in DMSO-d_6

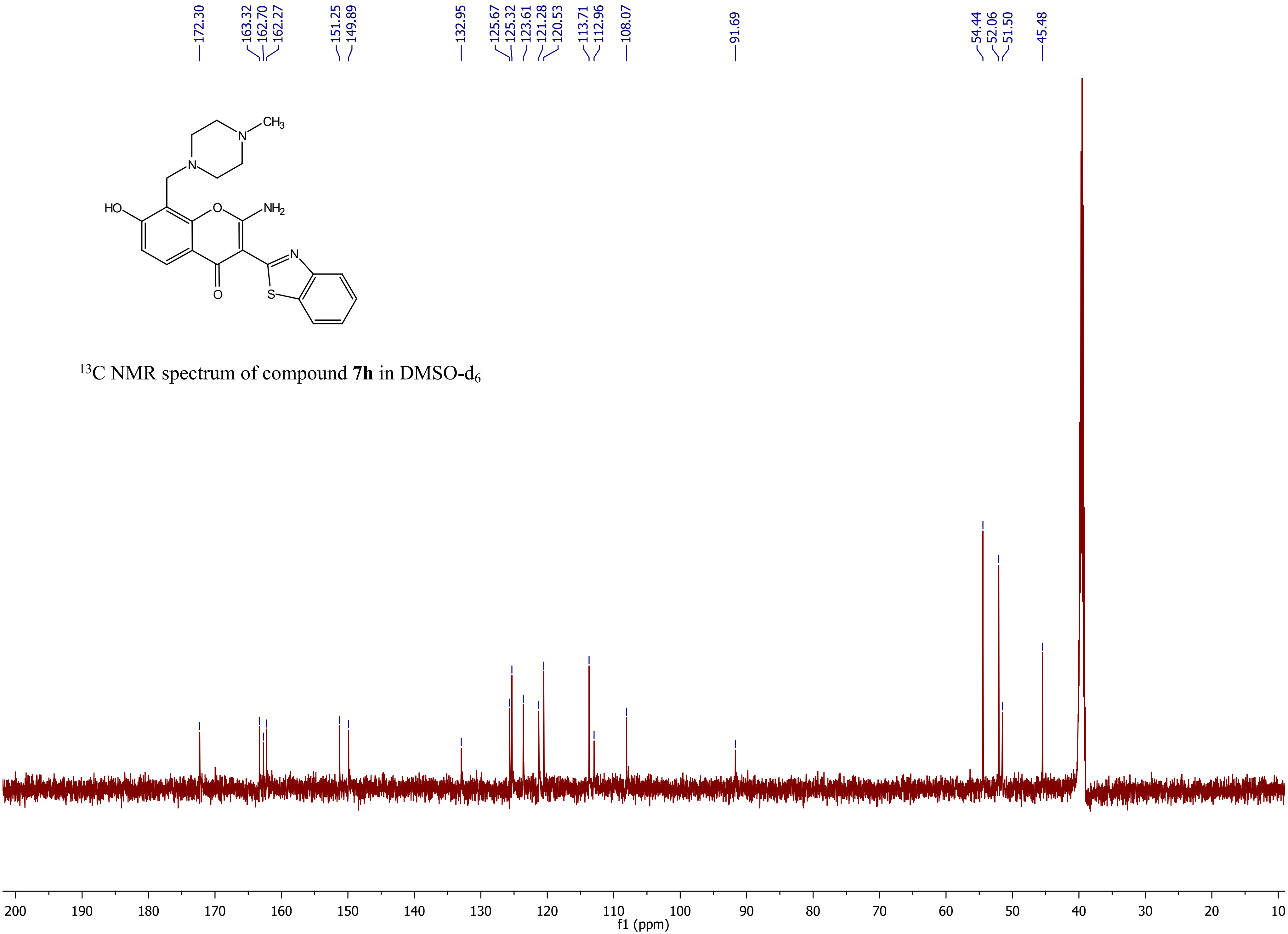

^{13}C NMR spectrum of compound **7h** in DMSO-d$_6$

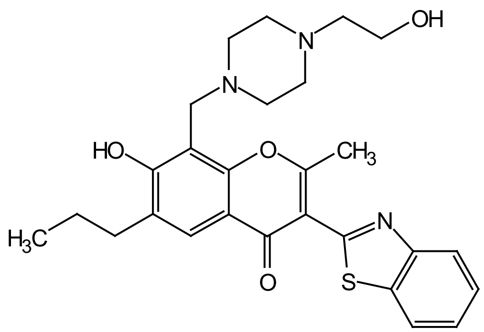

^{1}H NMR spectrun of compound **7i** in $CDCl_3$

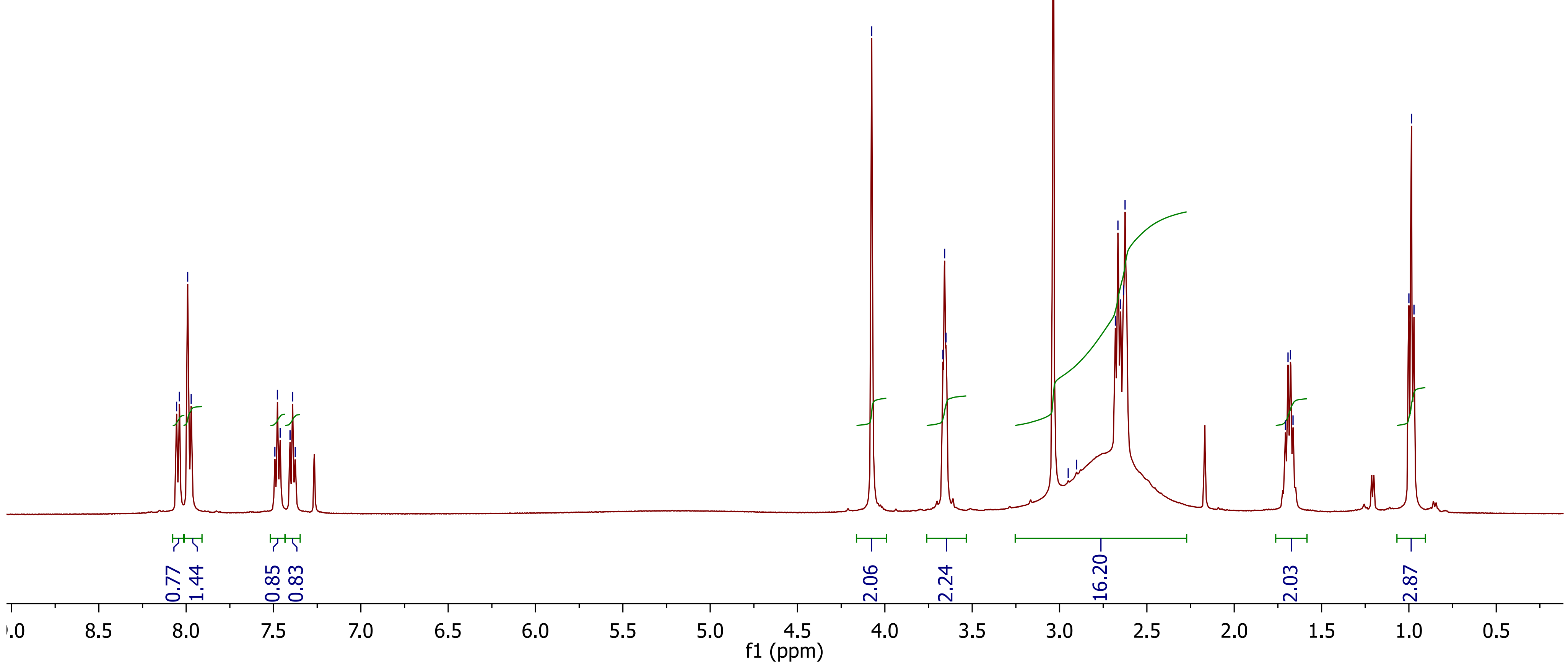

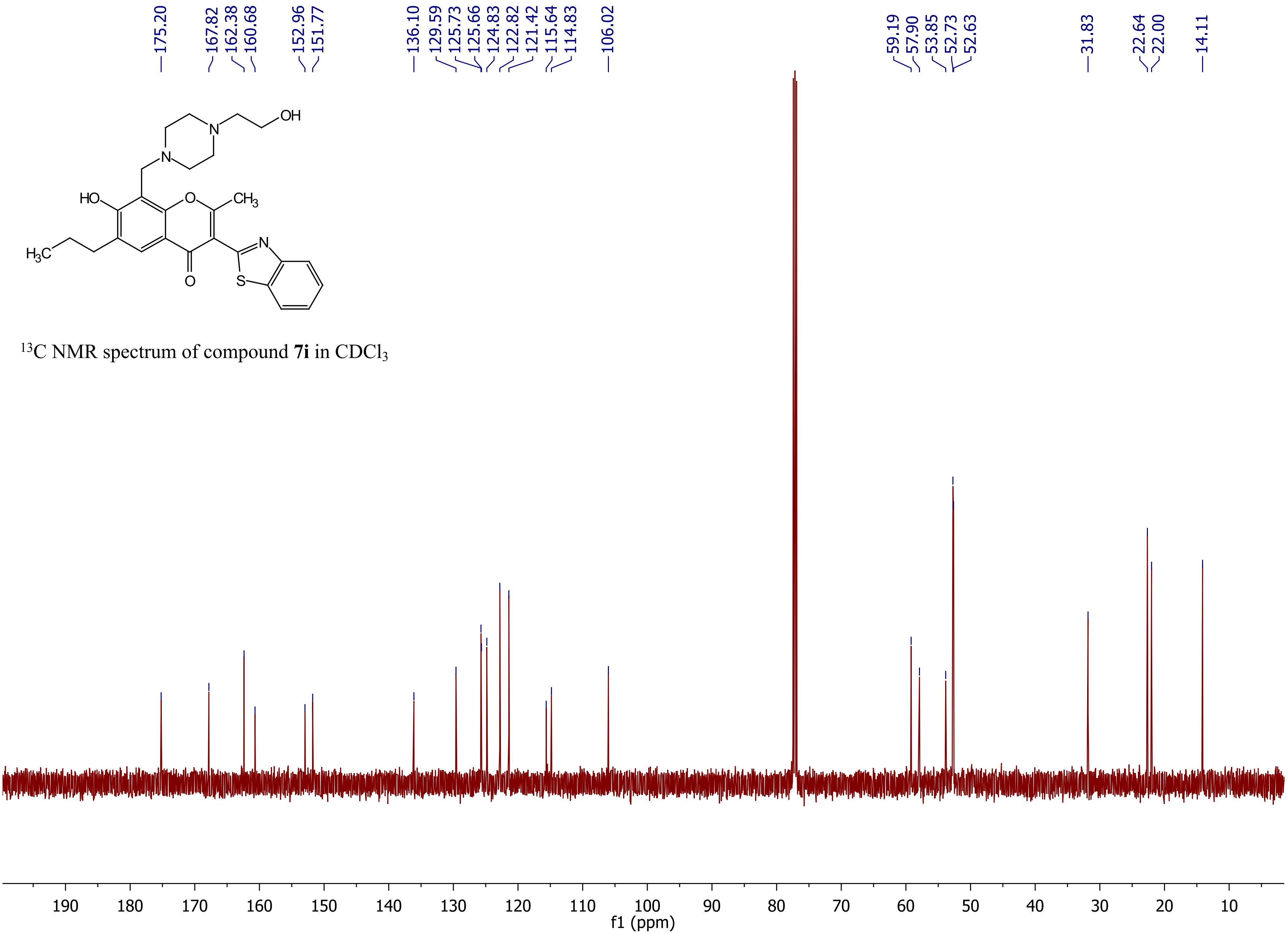

^{13}C NMR spectrum of compound **7i** in $CDCl_3$

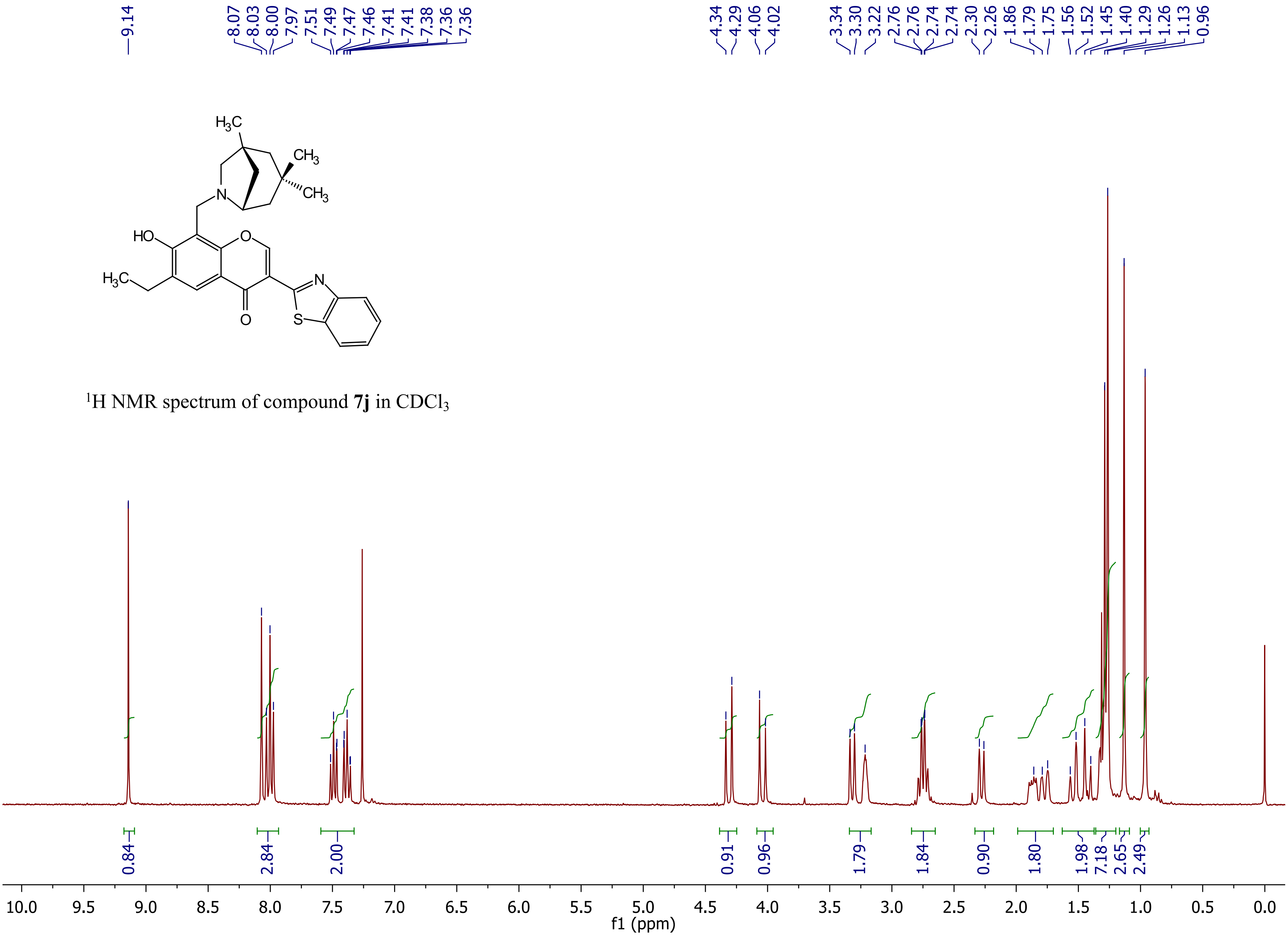

^{1}H NMR spectrum of compound **7j** in $CDCl_3$

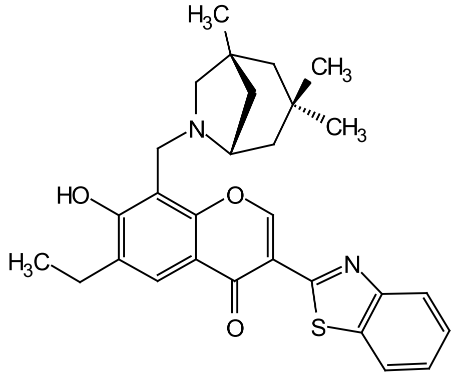

^{13}C NMR spectrum of compound **7j** in $CDCl_3$

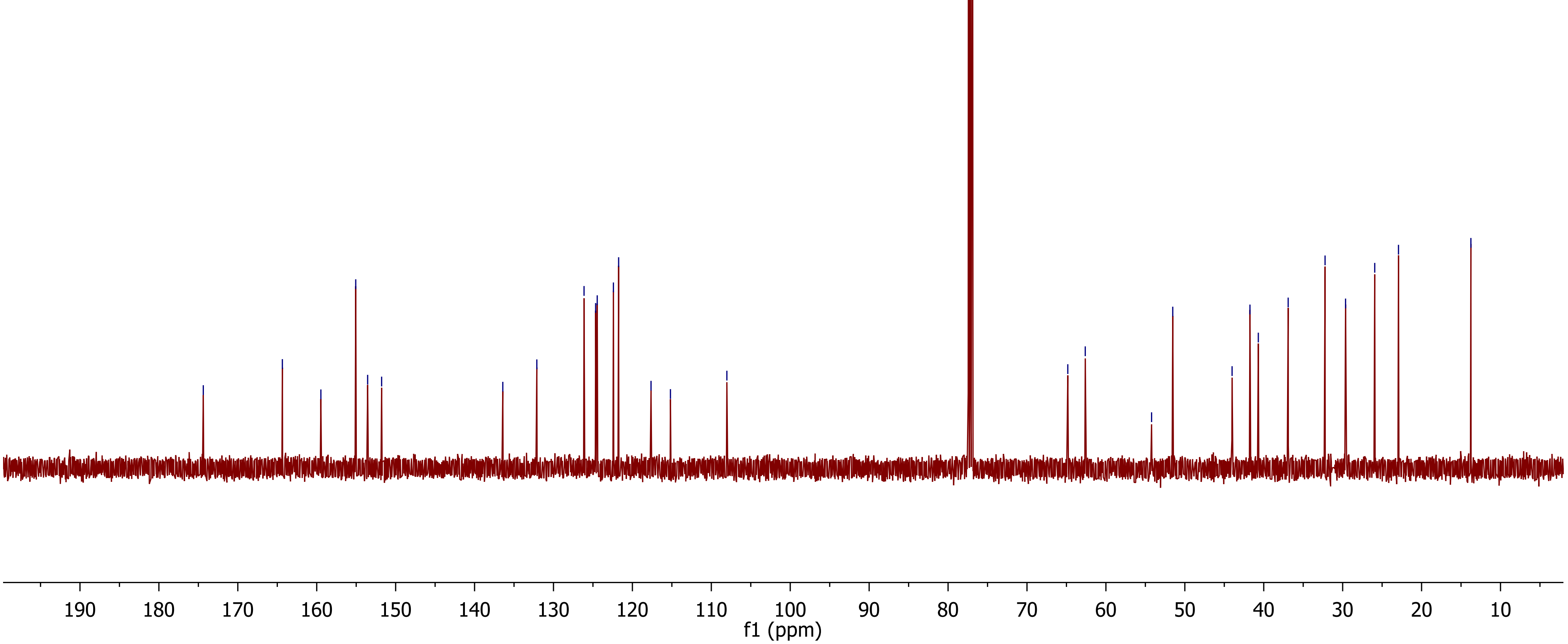

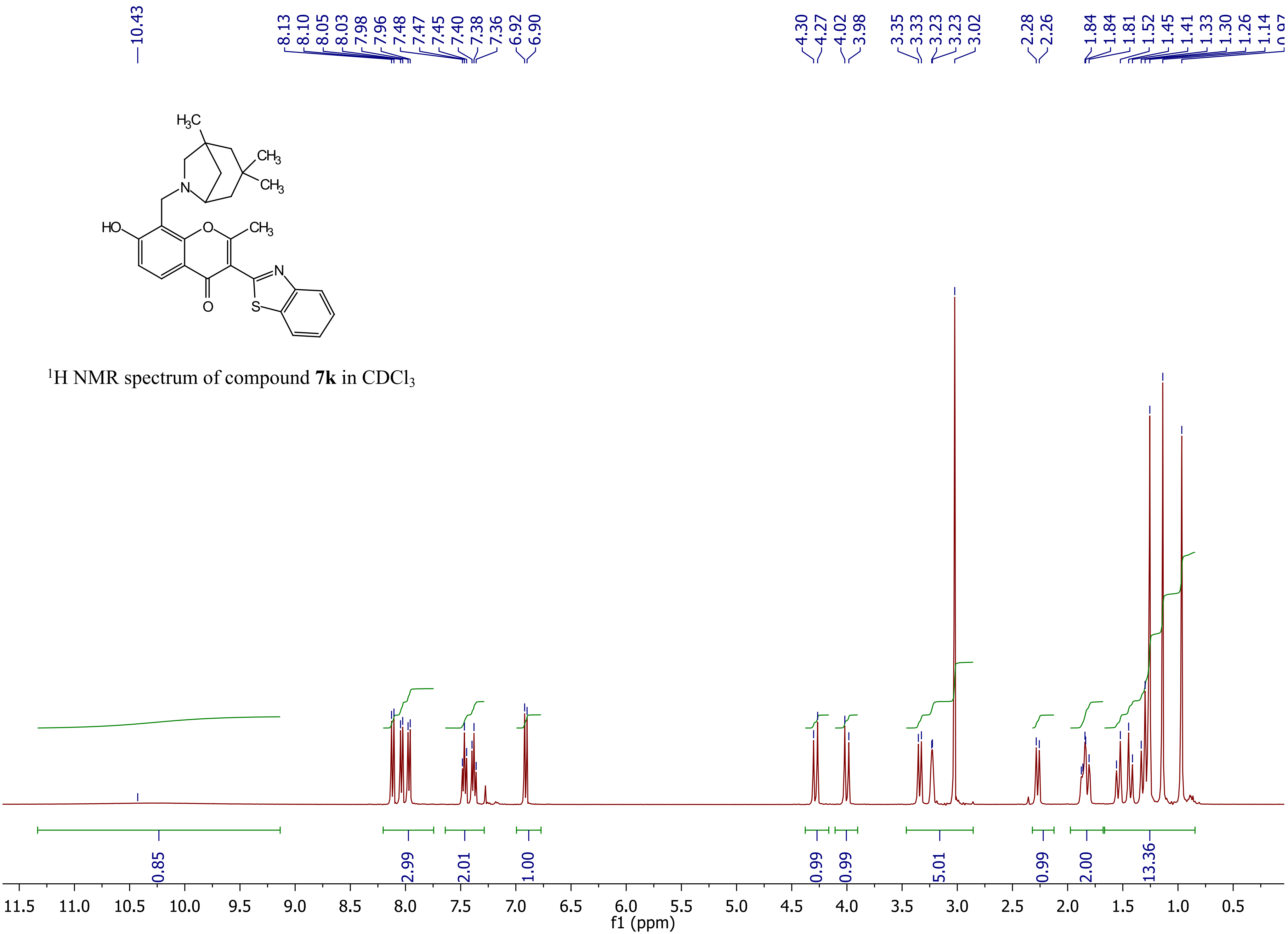

^{1}H NMR spectrum of compound **7k** in $CDCl_3$

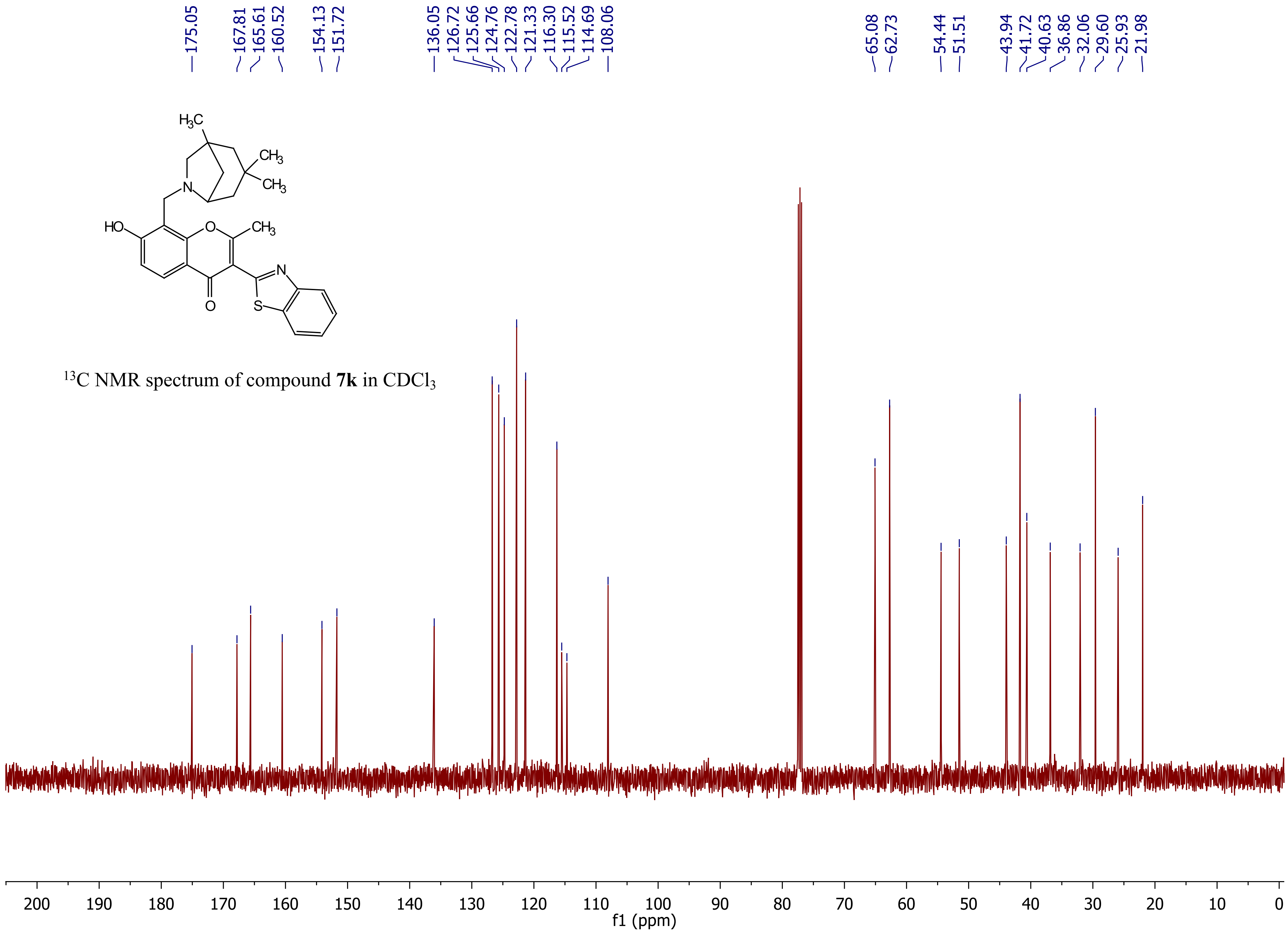

^{13}C NMR spectrum of compound **7k** in $CDCl_3$

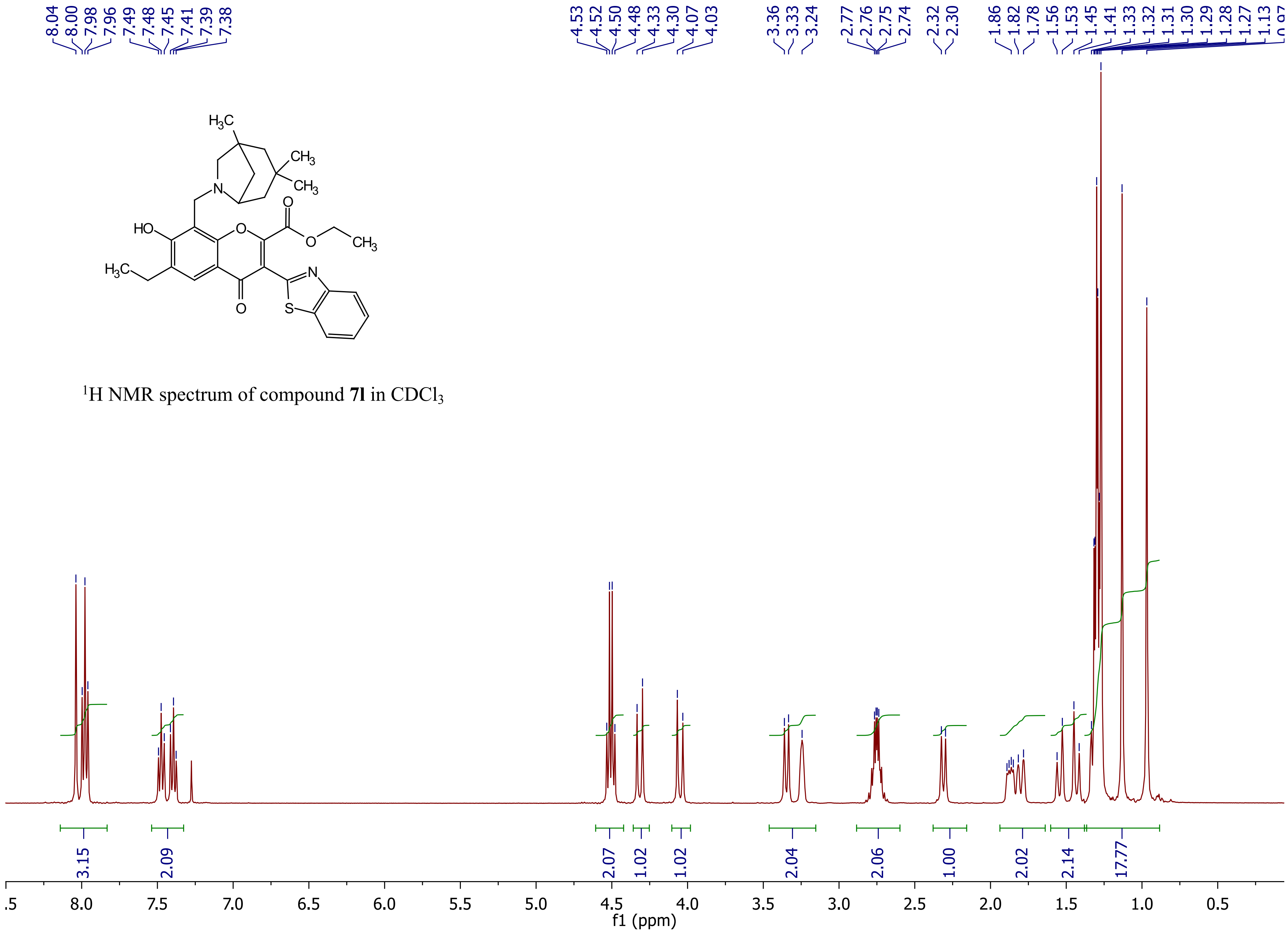

^{1}H NMR spectrum of compound **7l** in $CDCl_3$

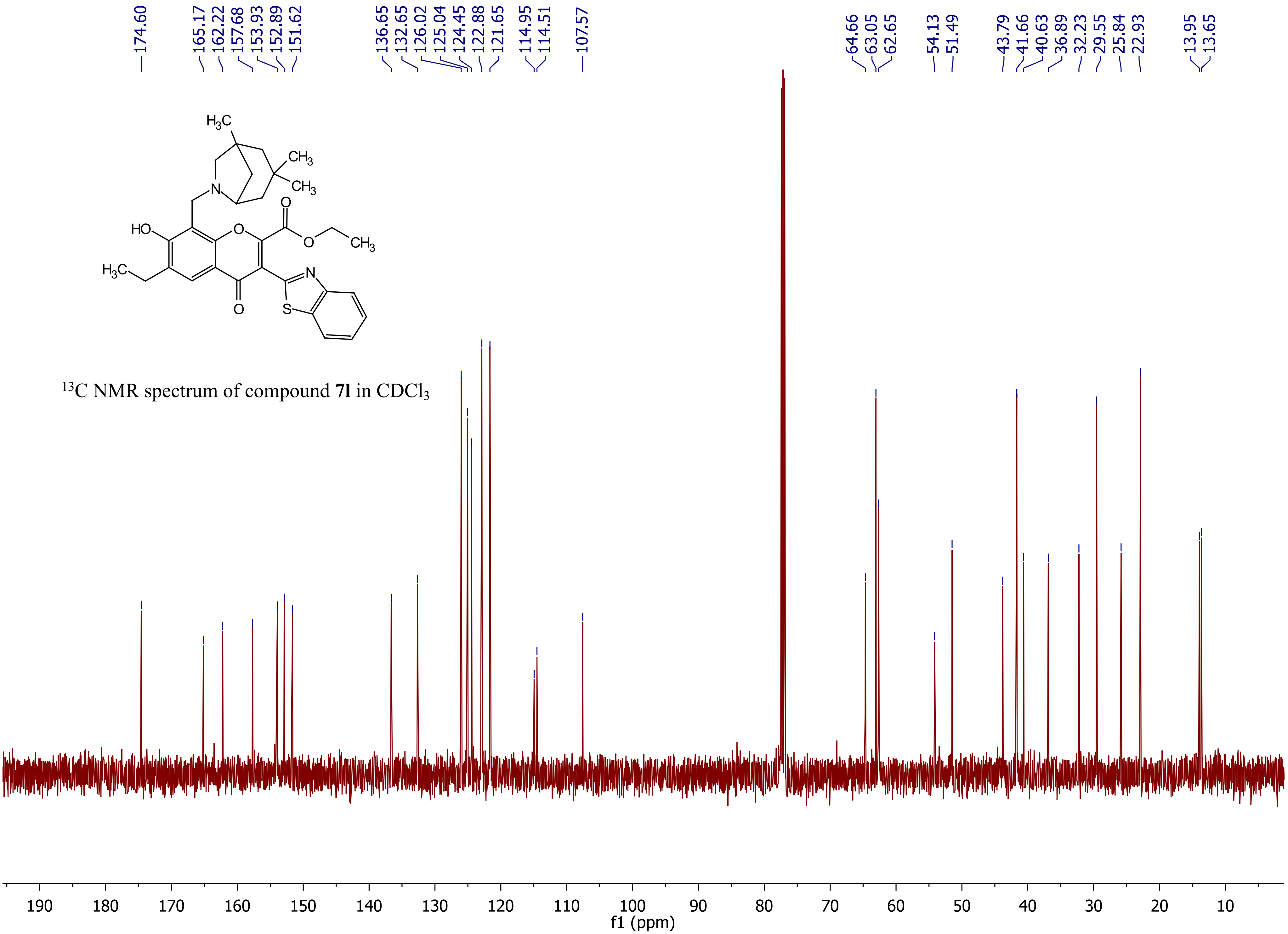

^{13}C NMR spectrum of compound **7l** in $CDCl_3$

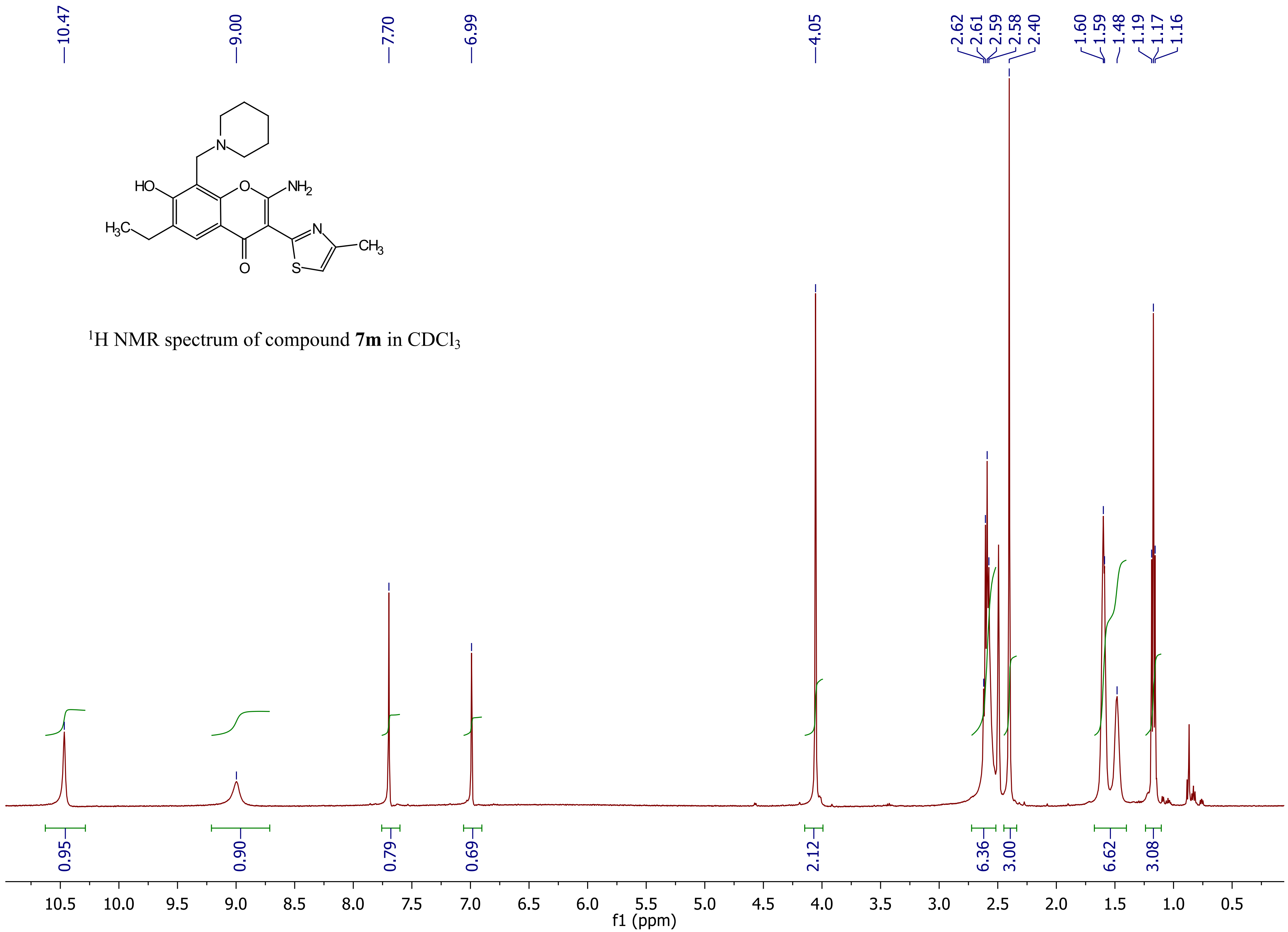

^{1}H NMR spectrum of compound **7m** in $CDCl_3$

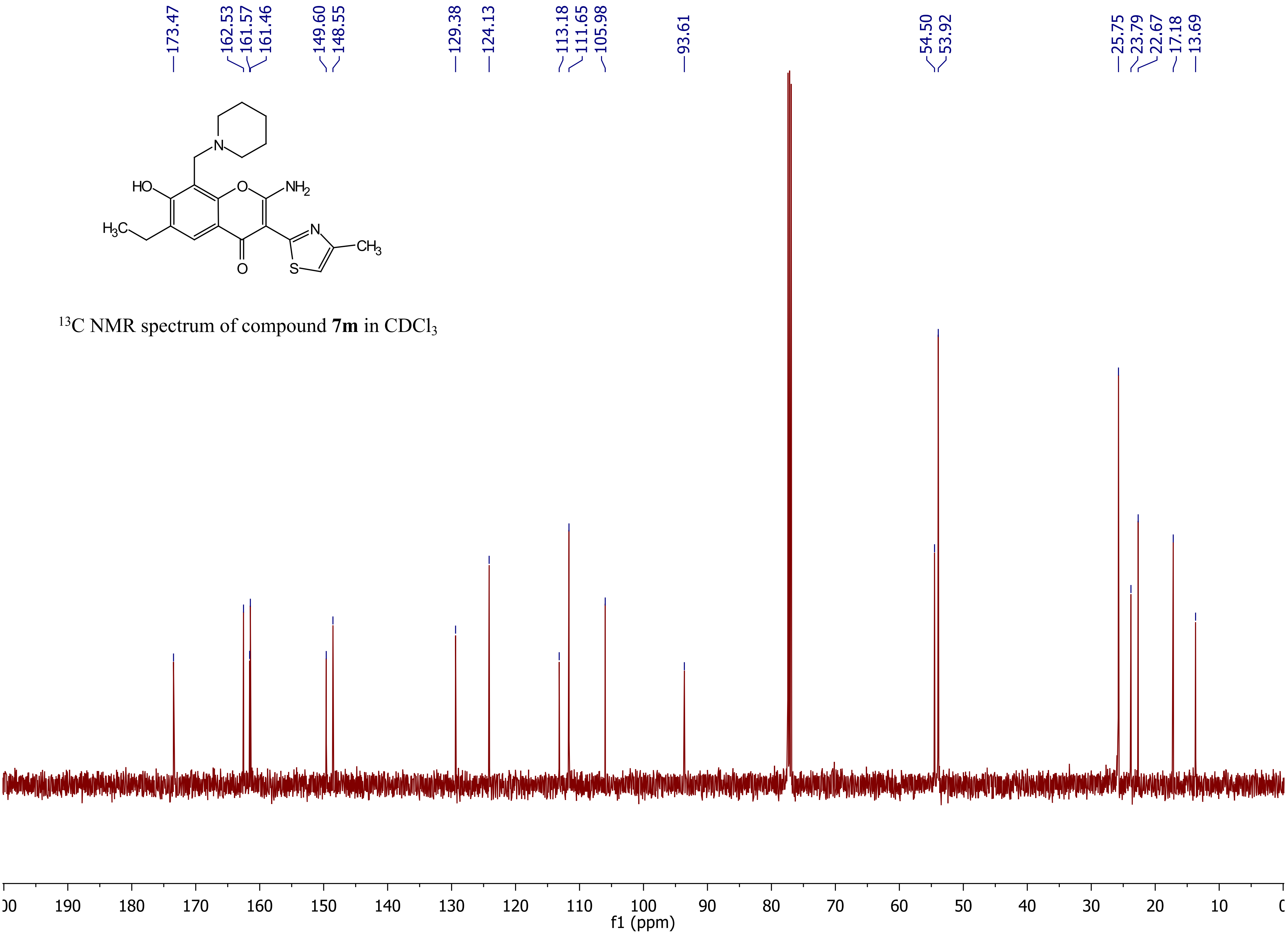

^{13}C NMR spectrum of compound **7m** in $CDCl_3$

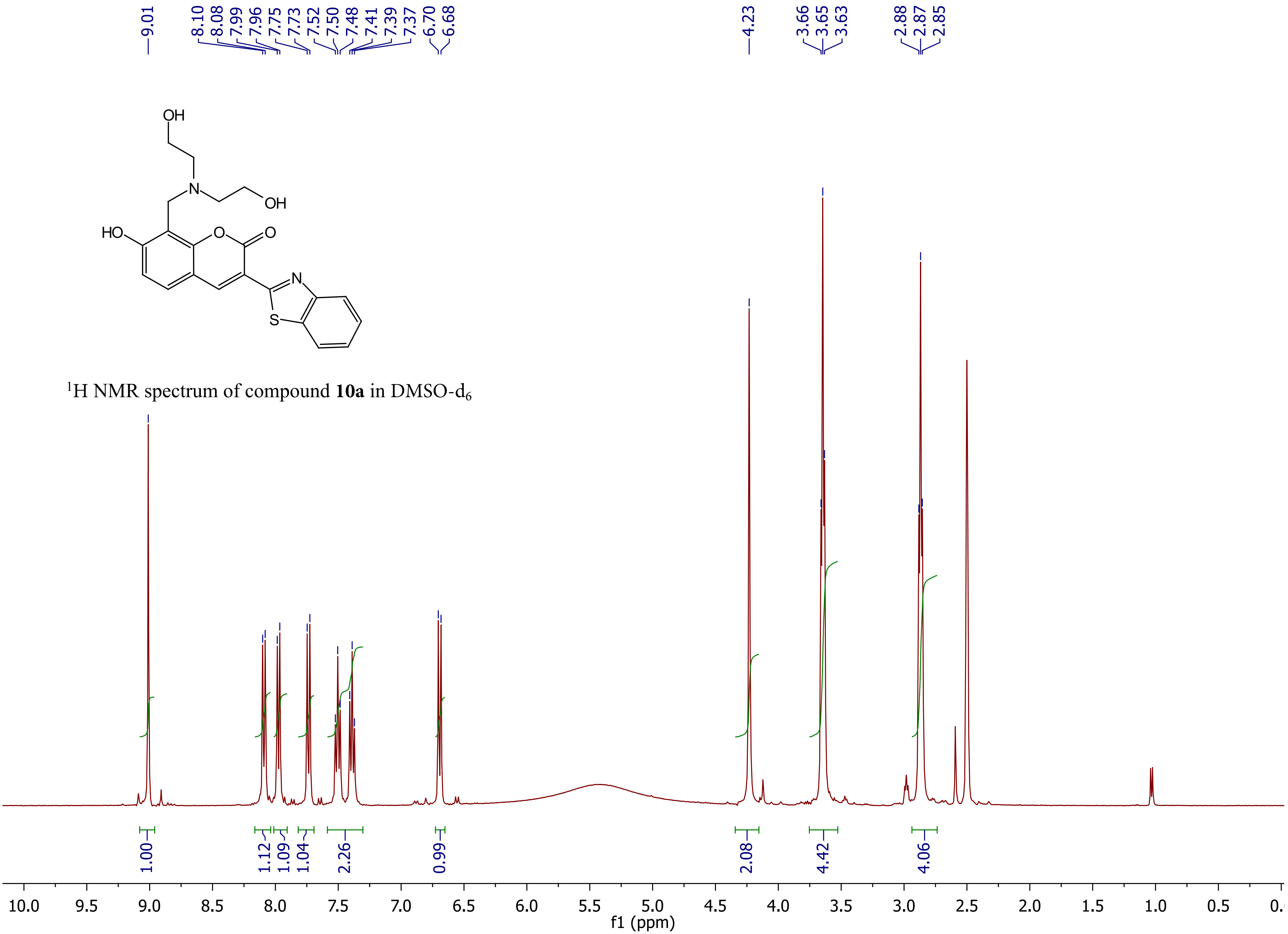

^{1}H NMR spectrum of compound **10a** in DMSO-d$_6$

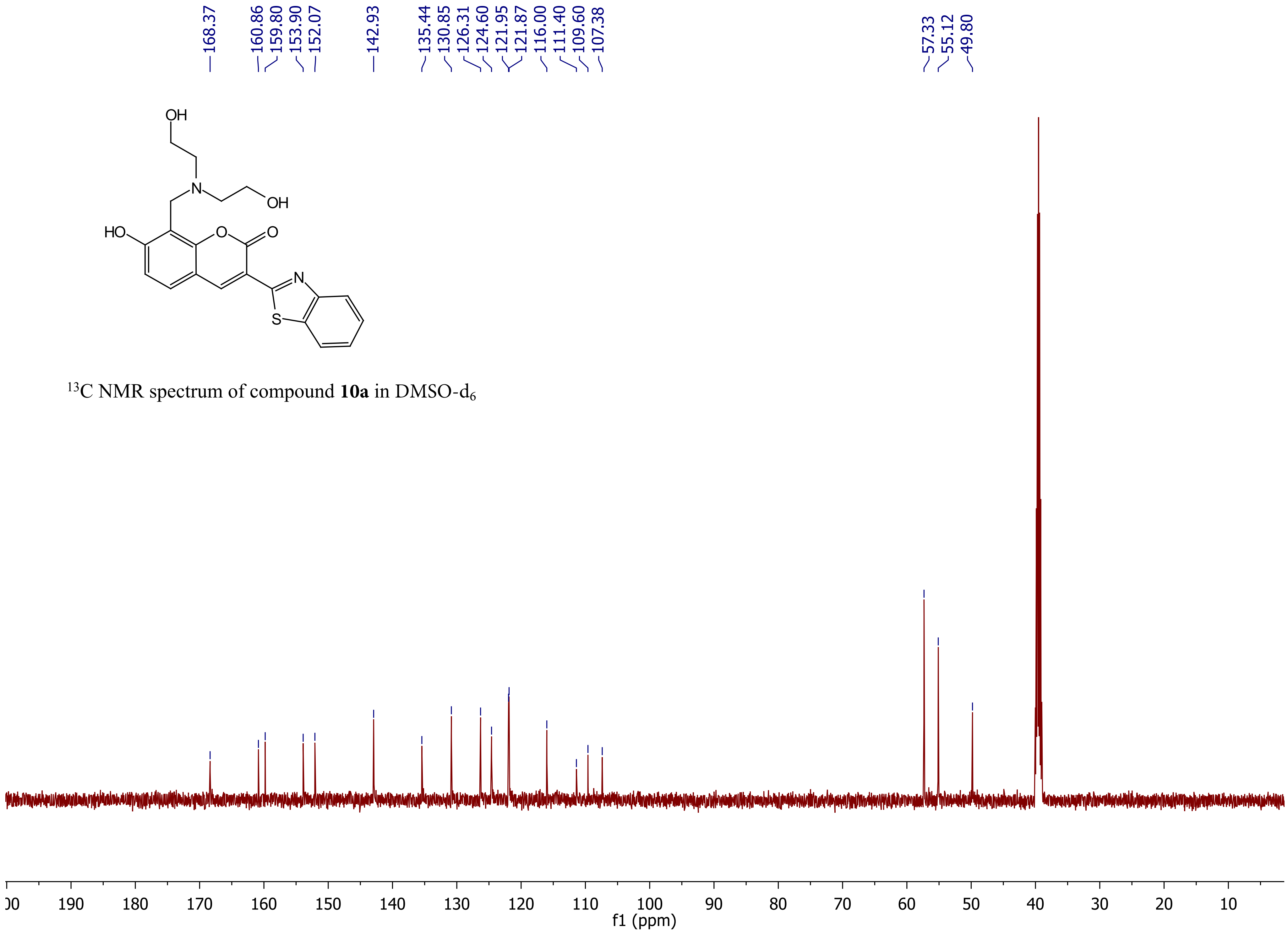

^{13}C NMR spectrum of compound **10a** in DMSO-d_6

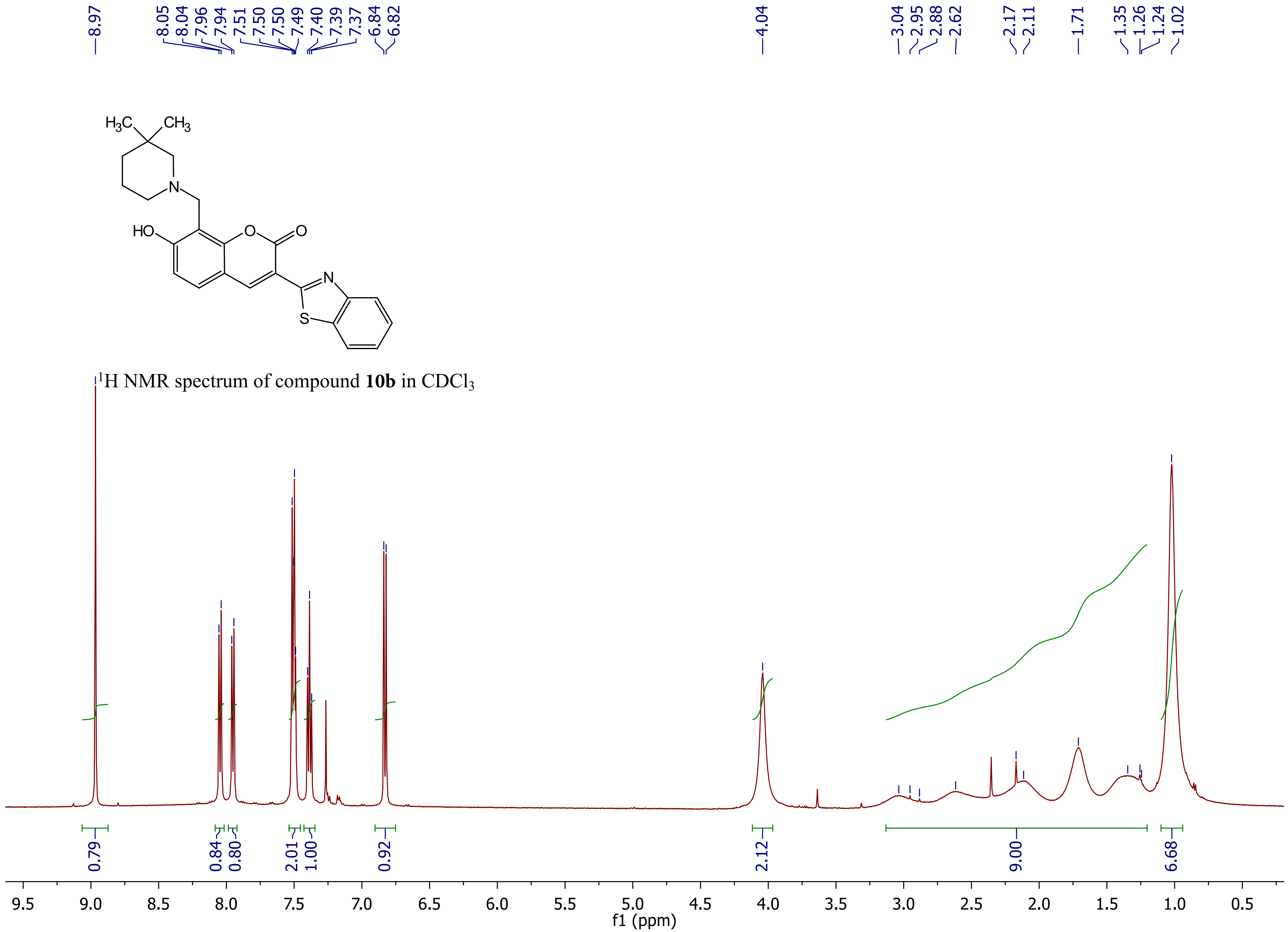

^{1}H NMR spectrum of compound **10b** in $CDCl_3$

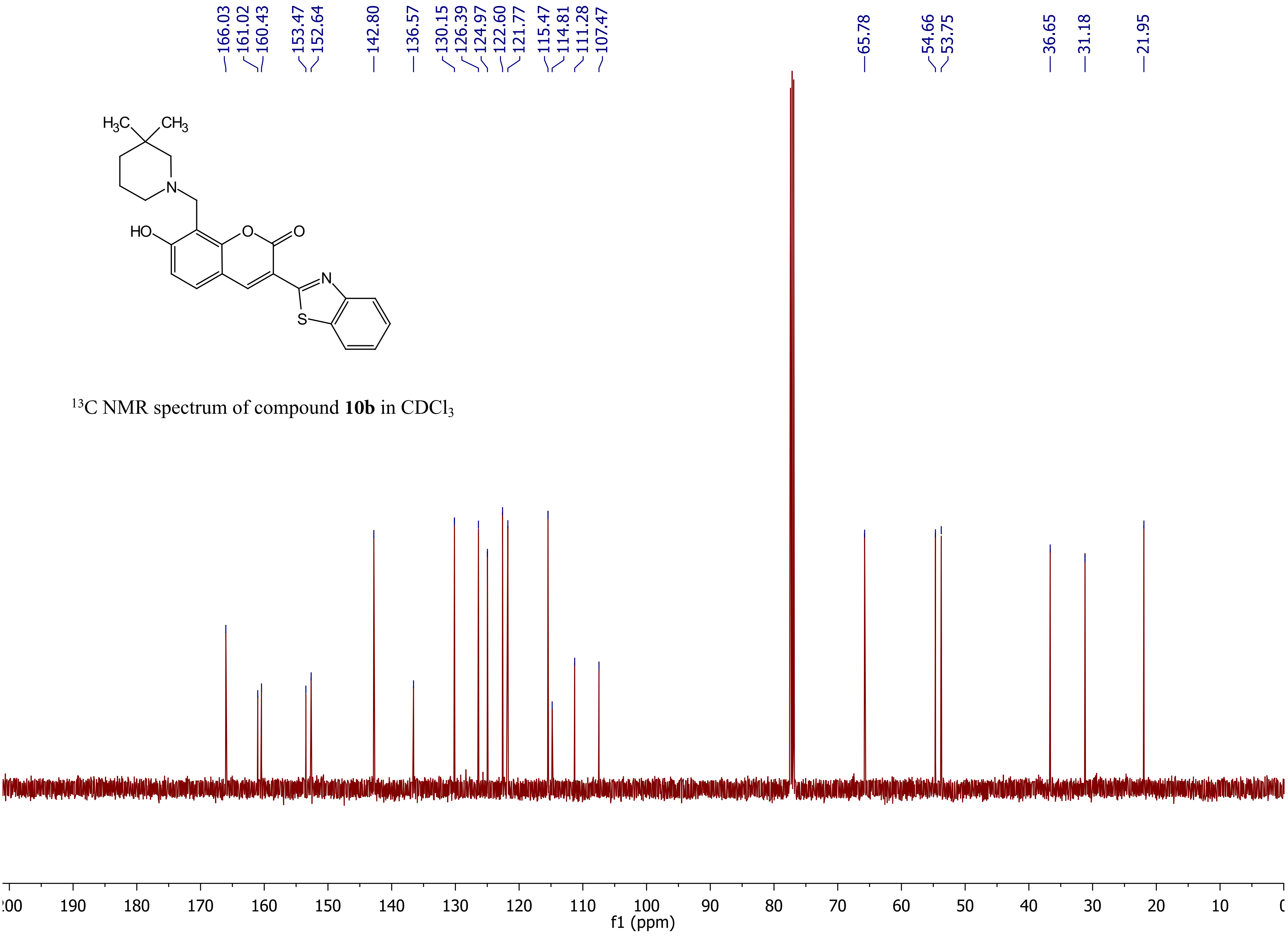

^{13}C NMR spectrum of compound **10b** in $CDCl_3$

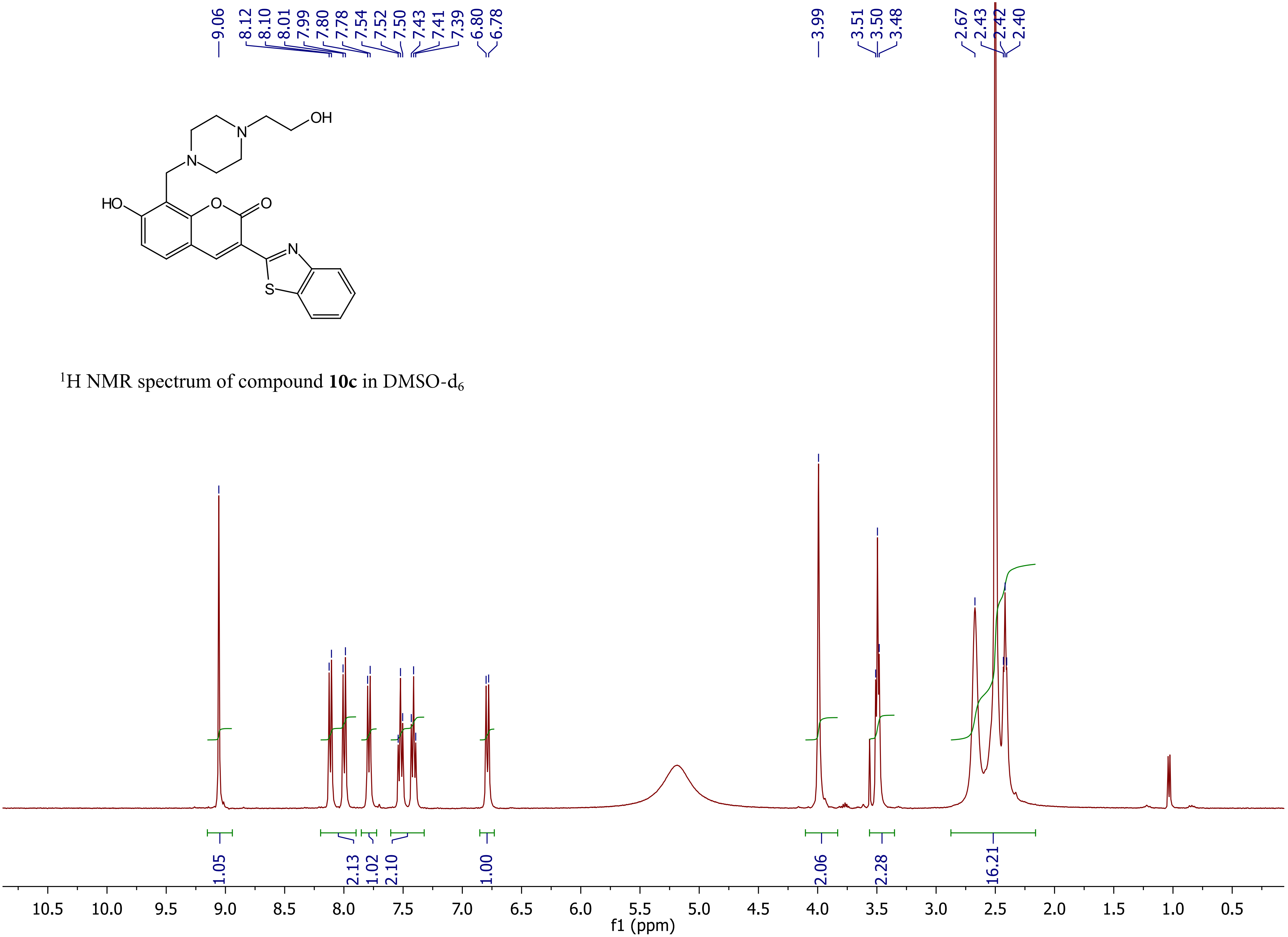

^{1}H NMR spectrum of compound **10c** in DMSO-d_6

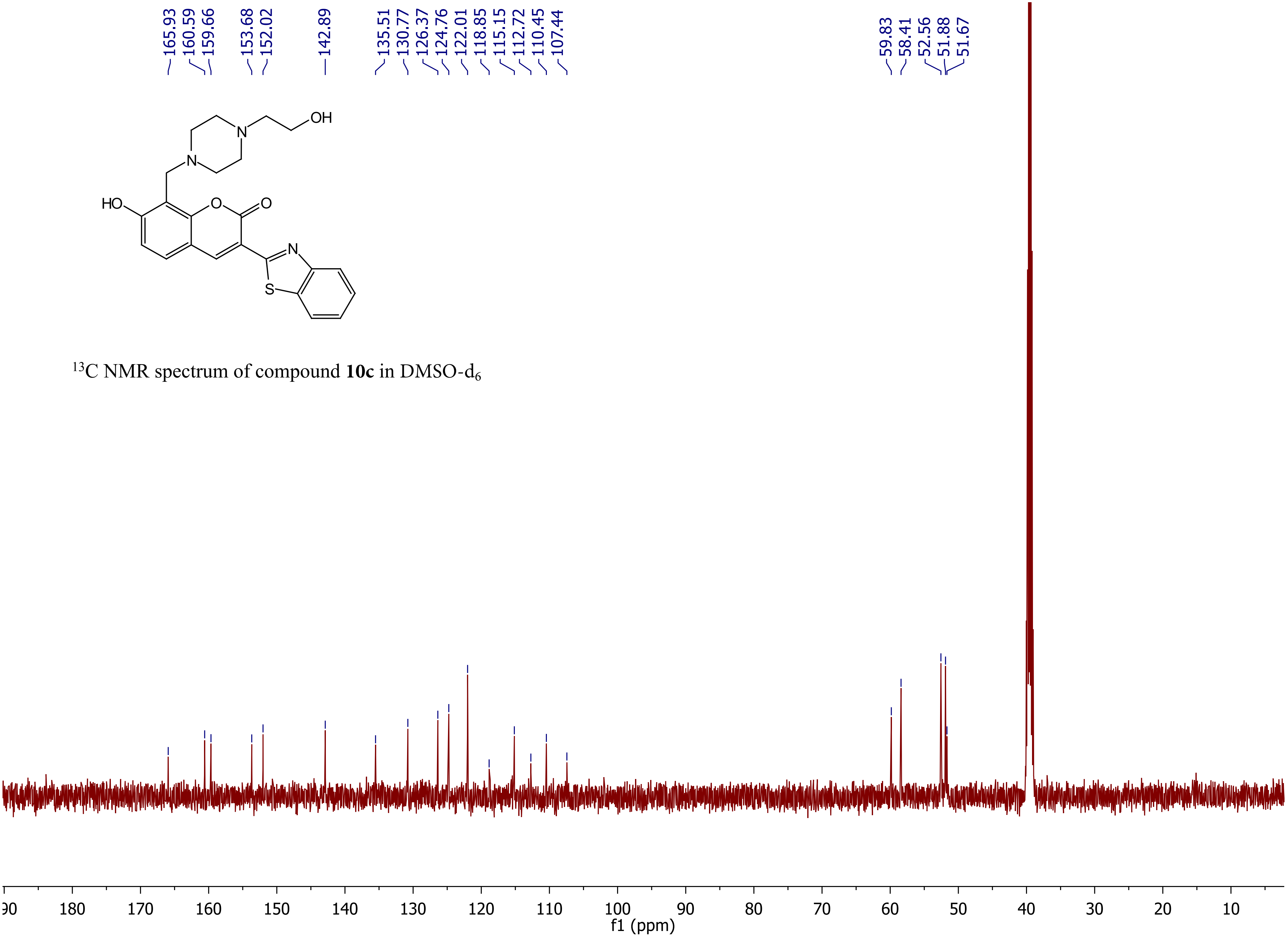

^{13}C NMR spectrum of compound **10c** in DMSO-d_6

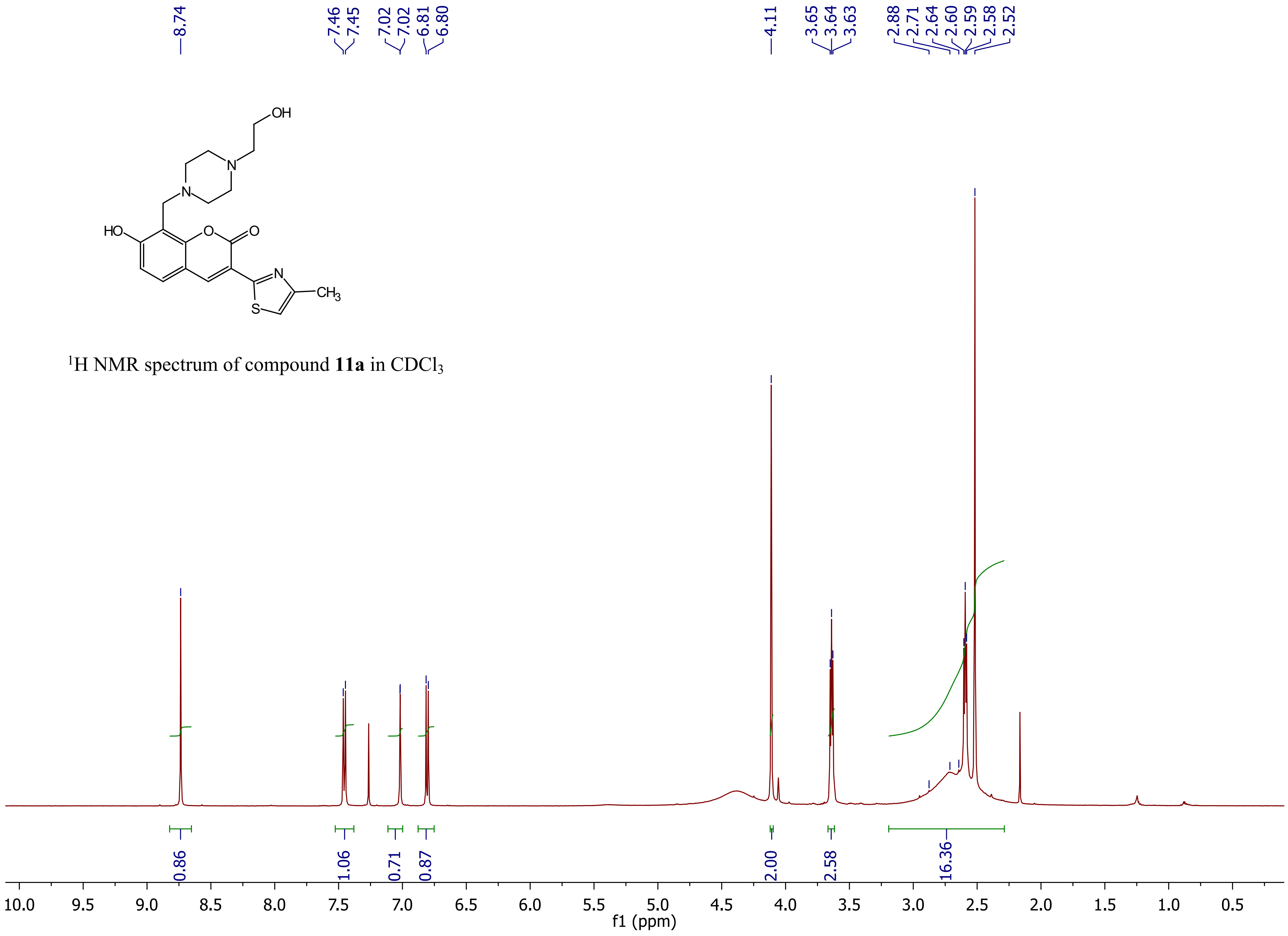

^{1}H NMR spectrum of compound **11a** in $CDCl_3$

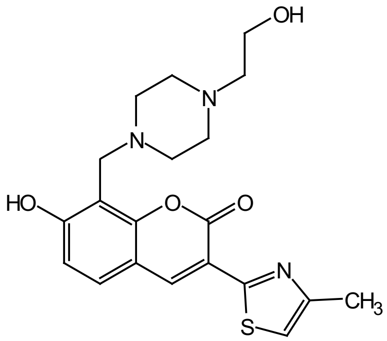

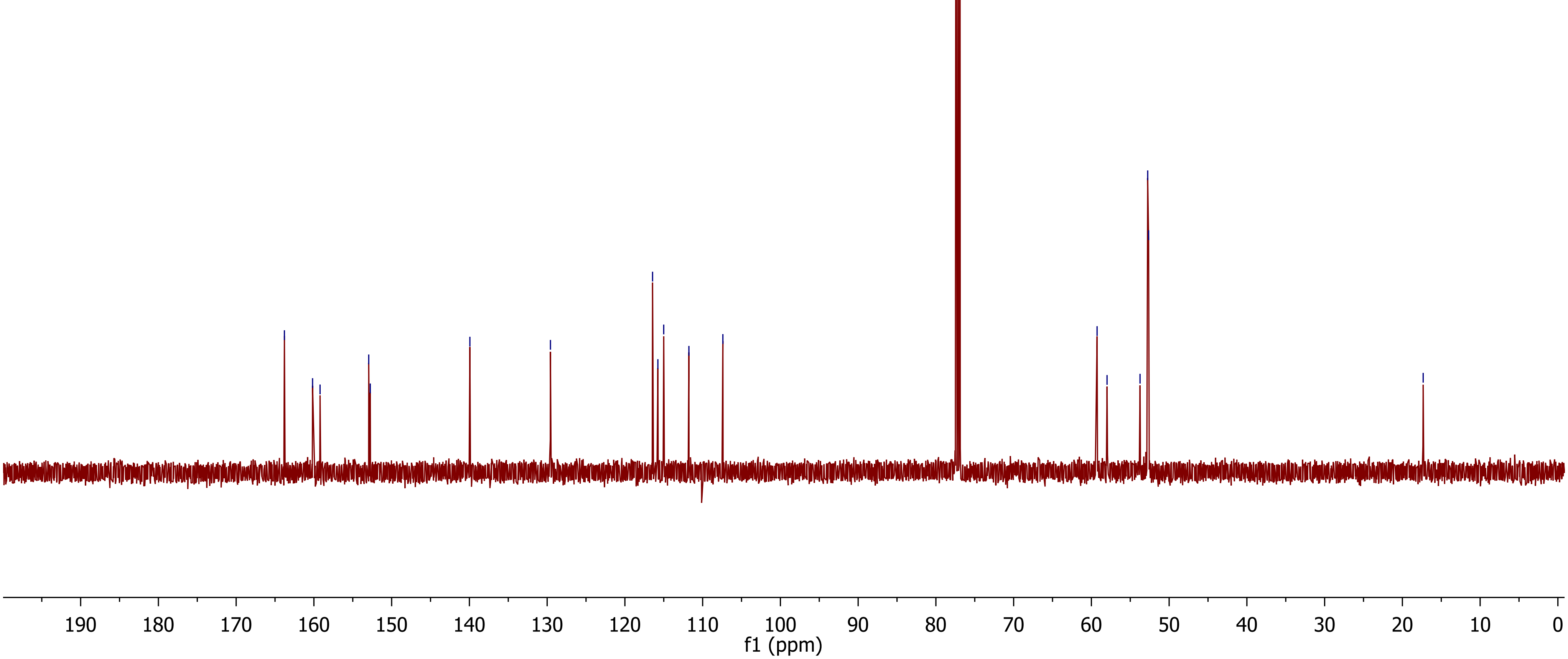

^{13}C NMR spectrum of compound **11a** in $CDCl_3$

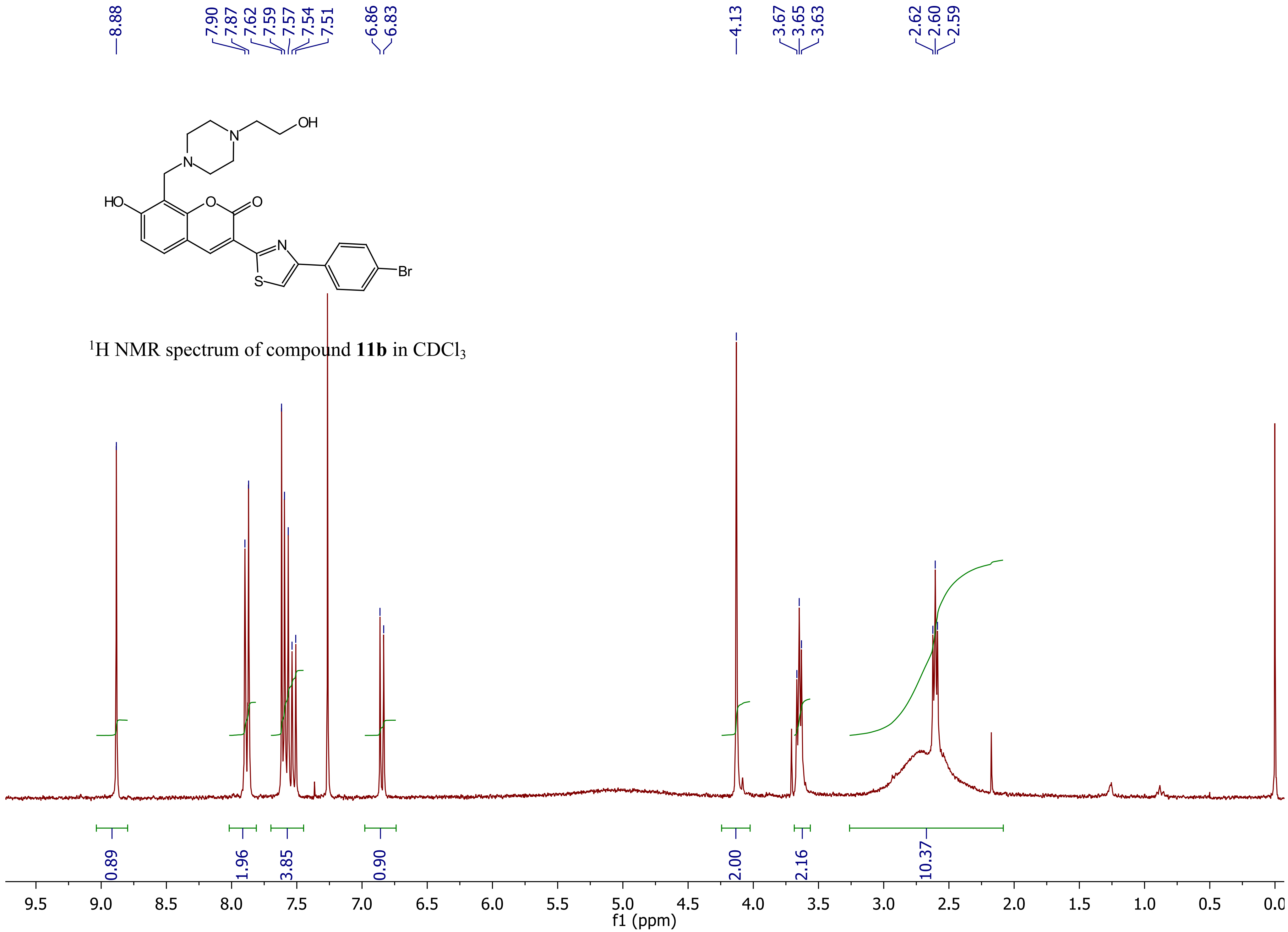

^{1}H NMR spectrum of compound **11b** in $CDCl_3$

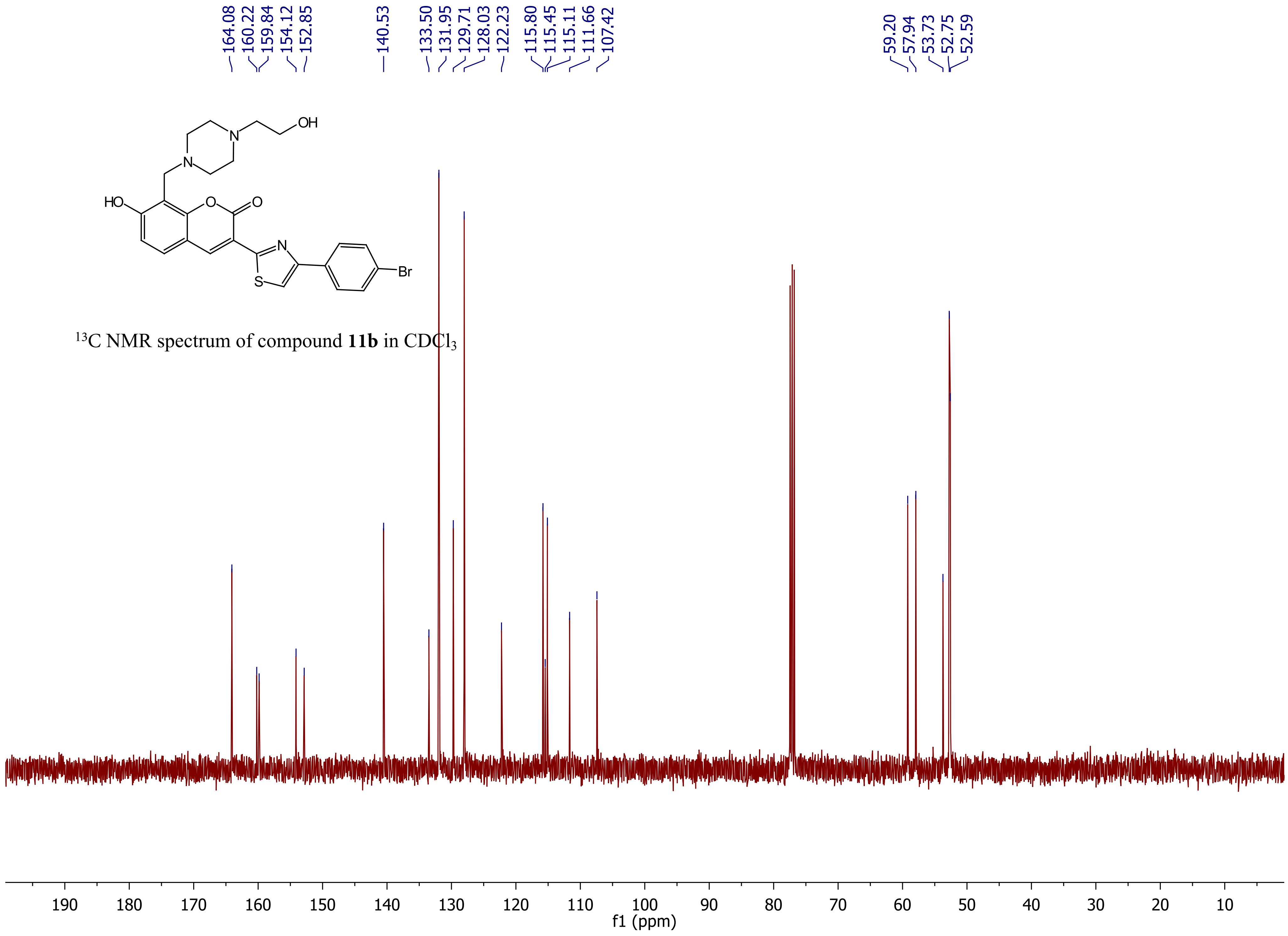

^{13}C NMR spectrum of compound **11b** in $CDCl_3$

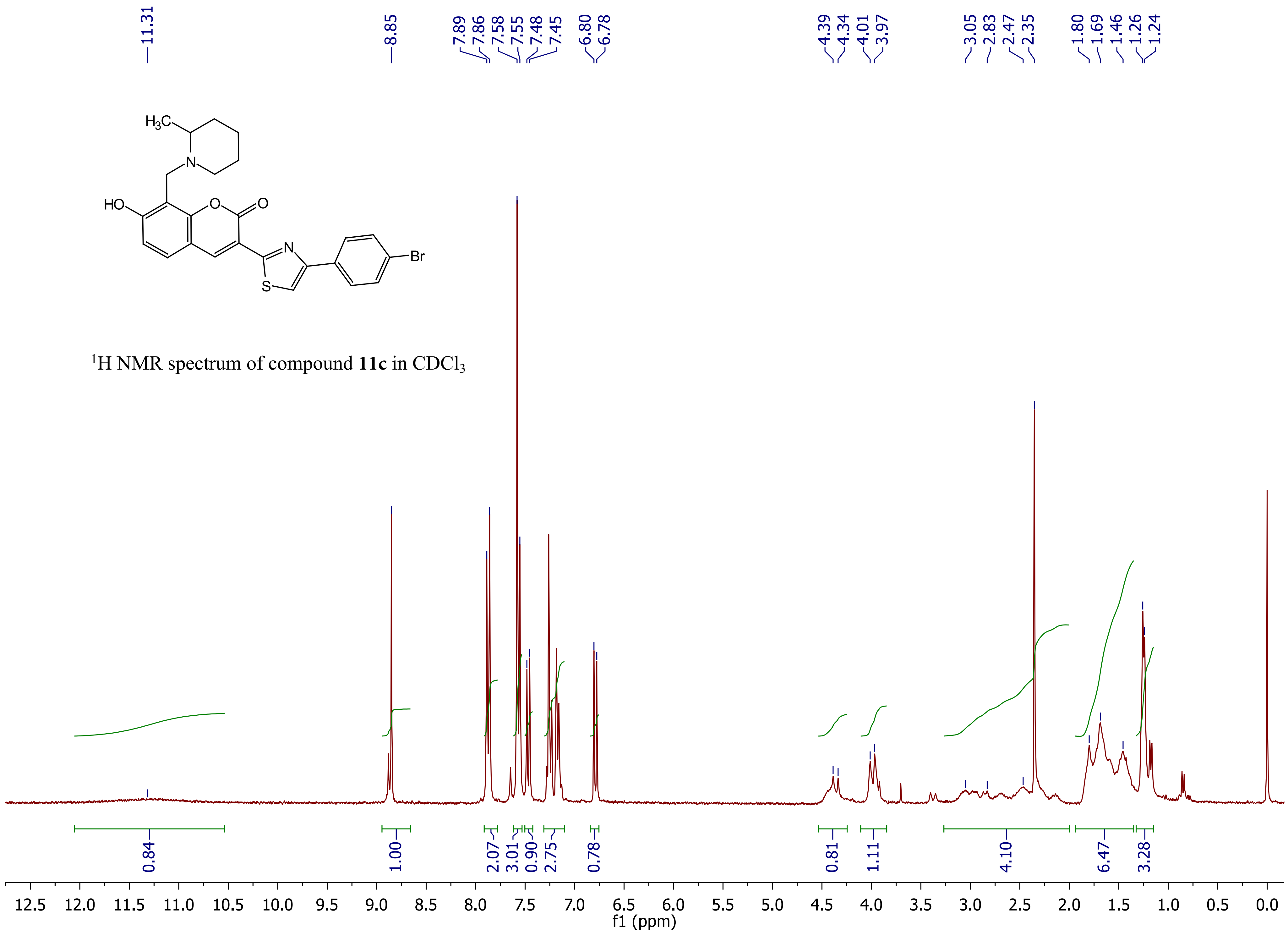

^{1}H NMR spectrum of compound **11c** in $CDCl_3$

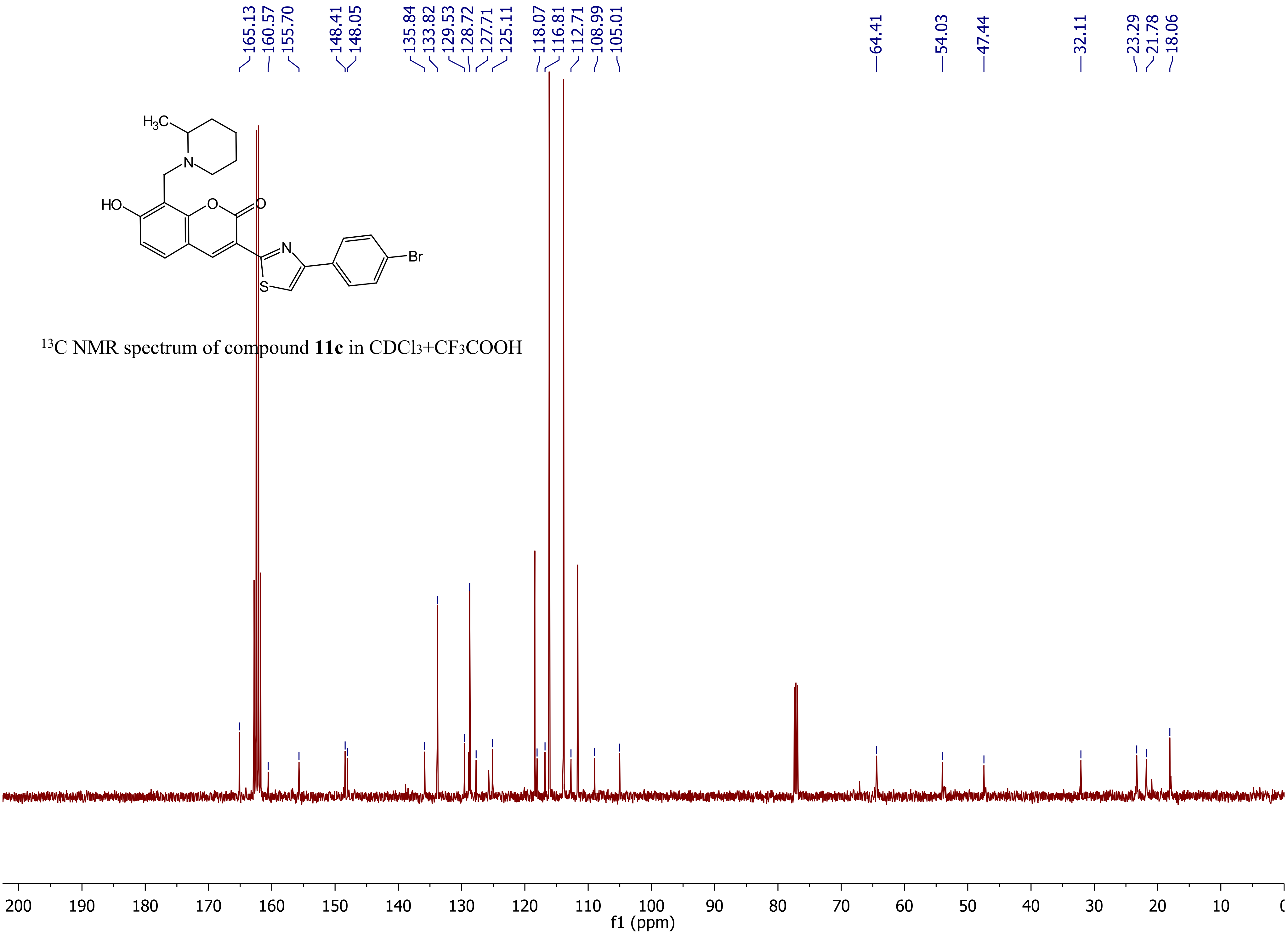

^{13}C NMR spectrum of compound **11c** in $CDCl_3$+CF_3COOH

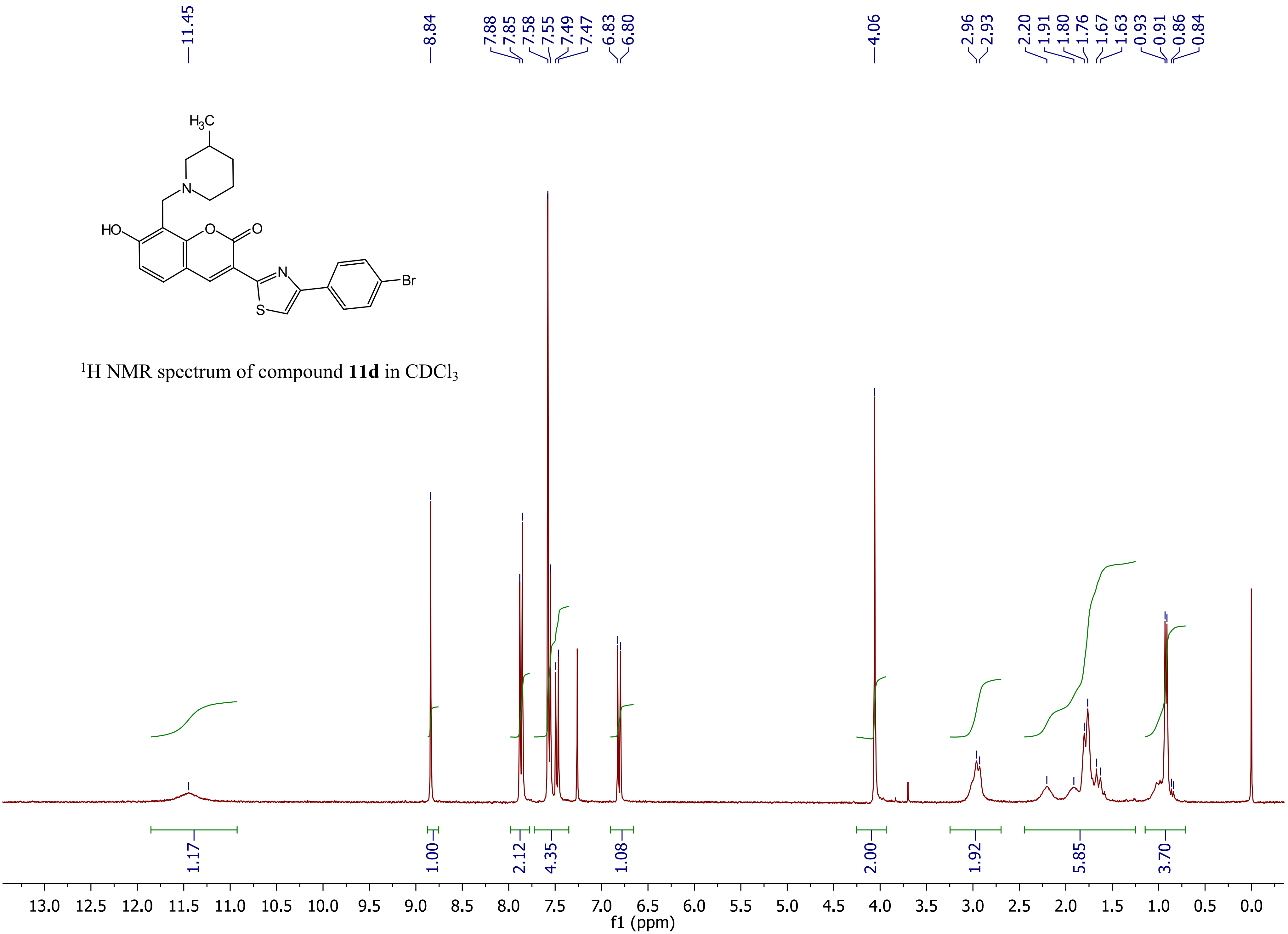

^{1}H NMR spectrum of compound **11d** in $CDCl_3$

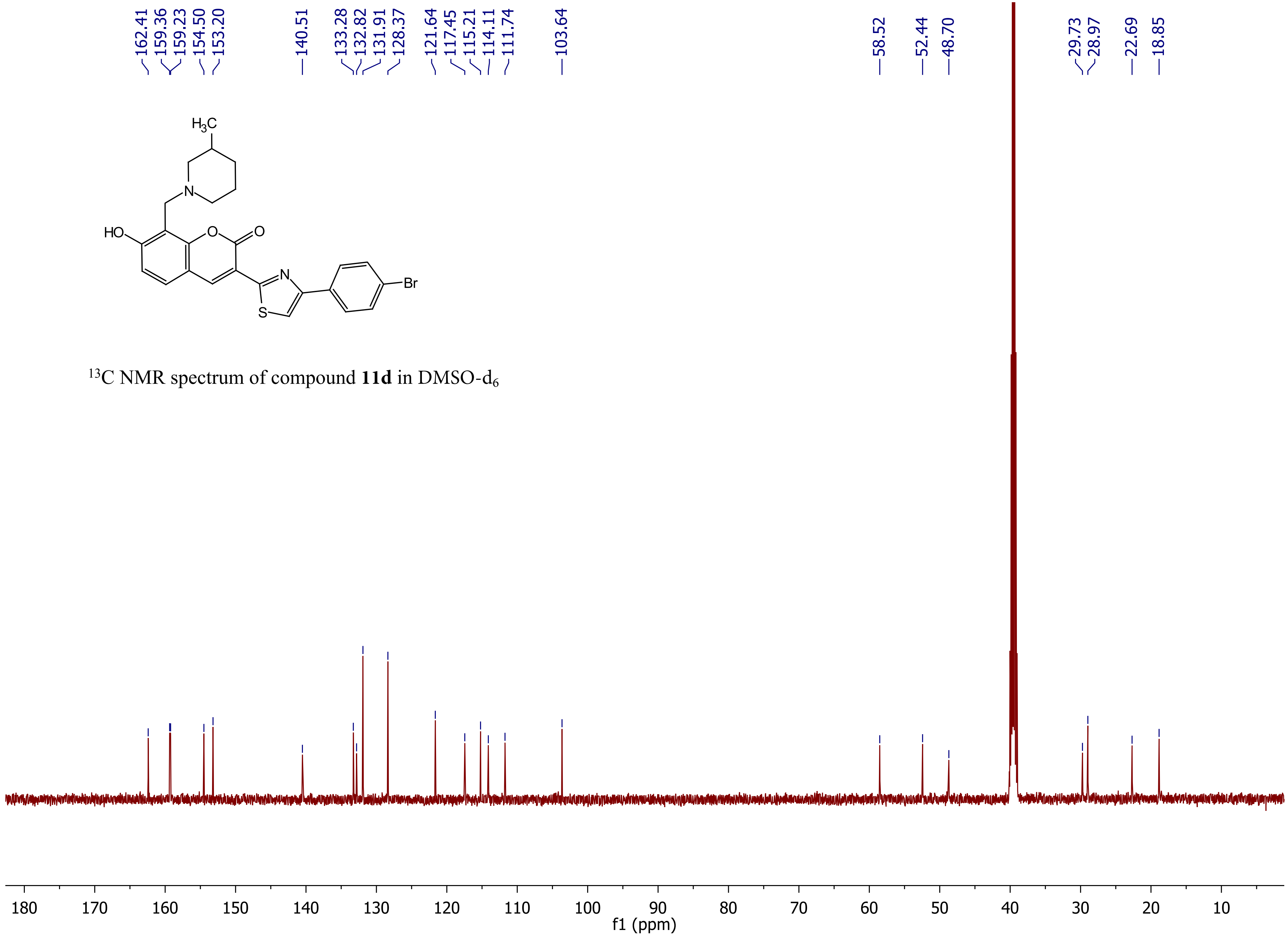

^{13}C NMR spectrum of compound **11d** in DMSO-d_6

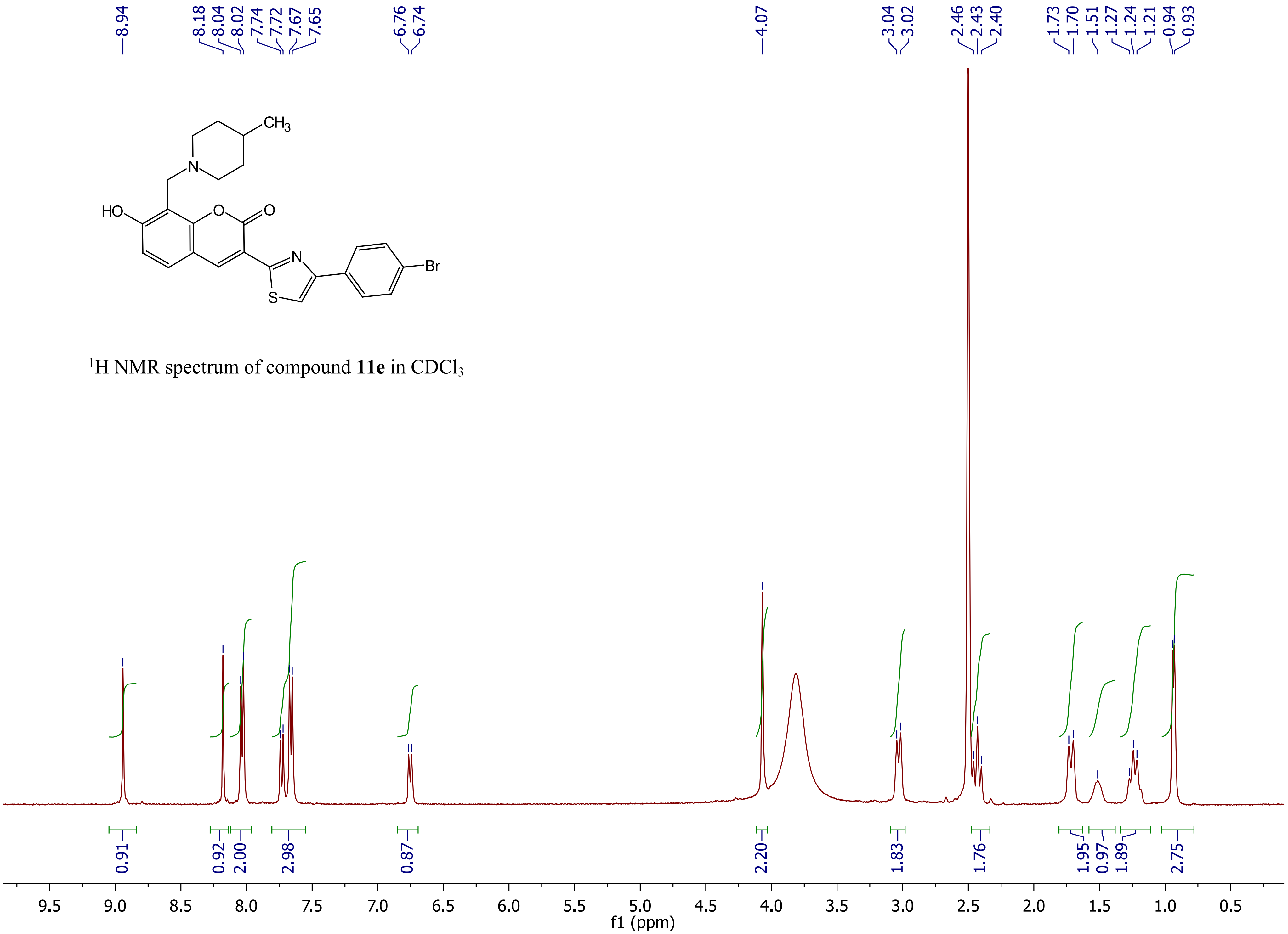

^{1}H NMR spectrum of compound **11e** in $CDCl_3$

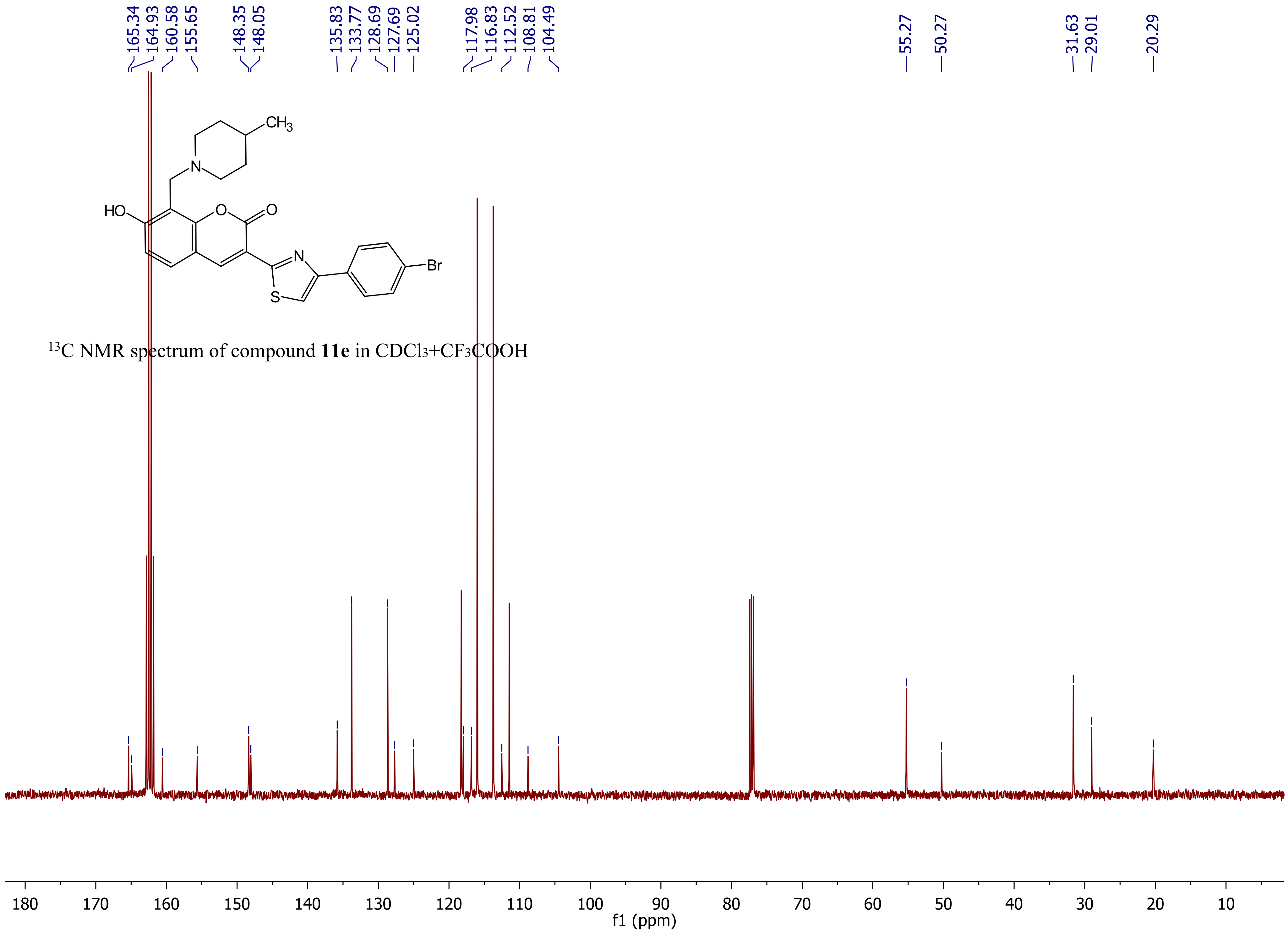

^{13}C NMR spectrum of compound **11e** in $CDCl_3$+CF_3COOH

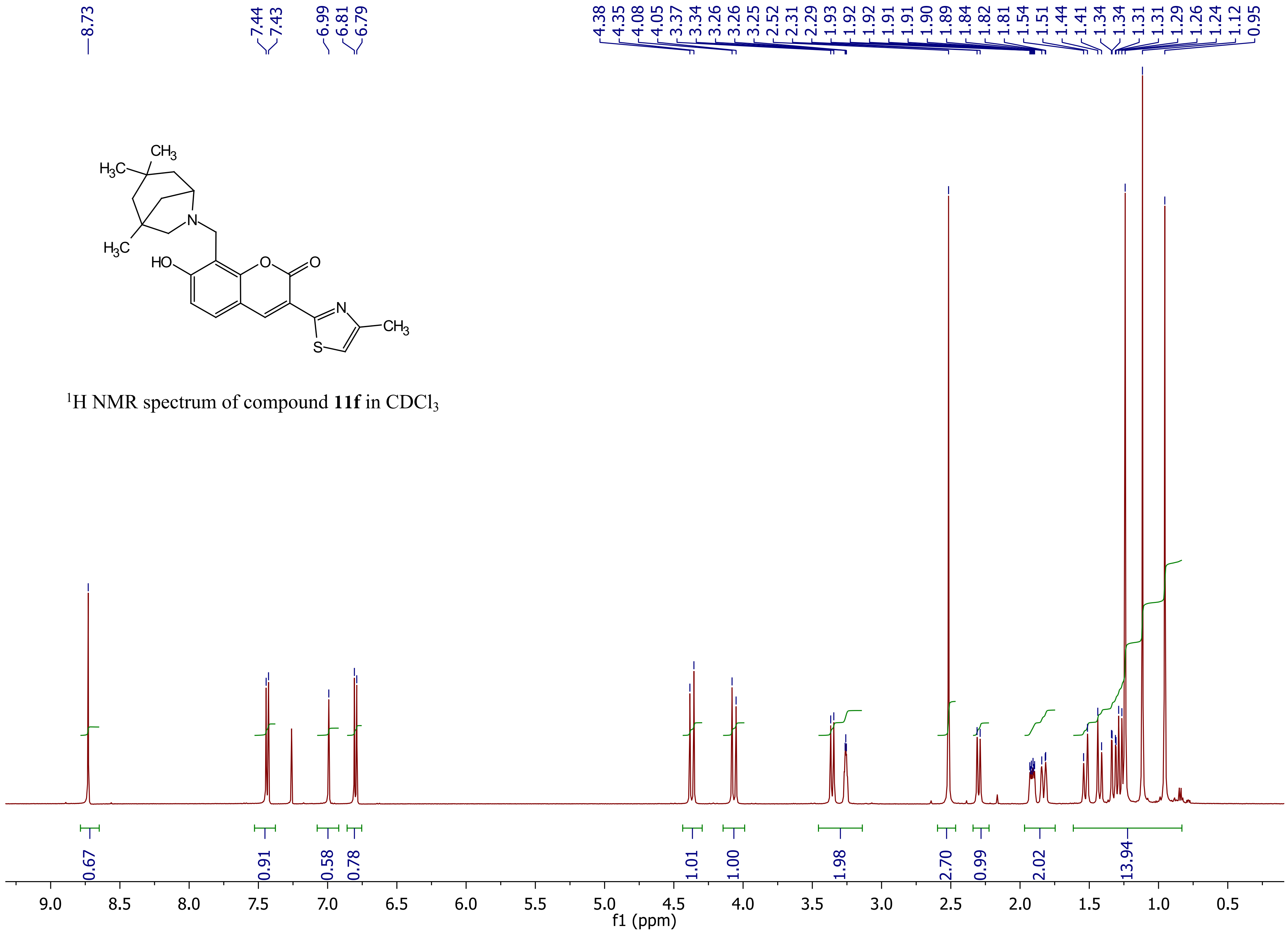

^{1}H NMR spectrum of compound **11f** in $CDCl_3$

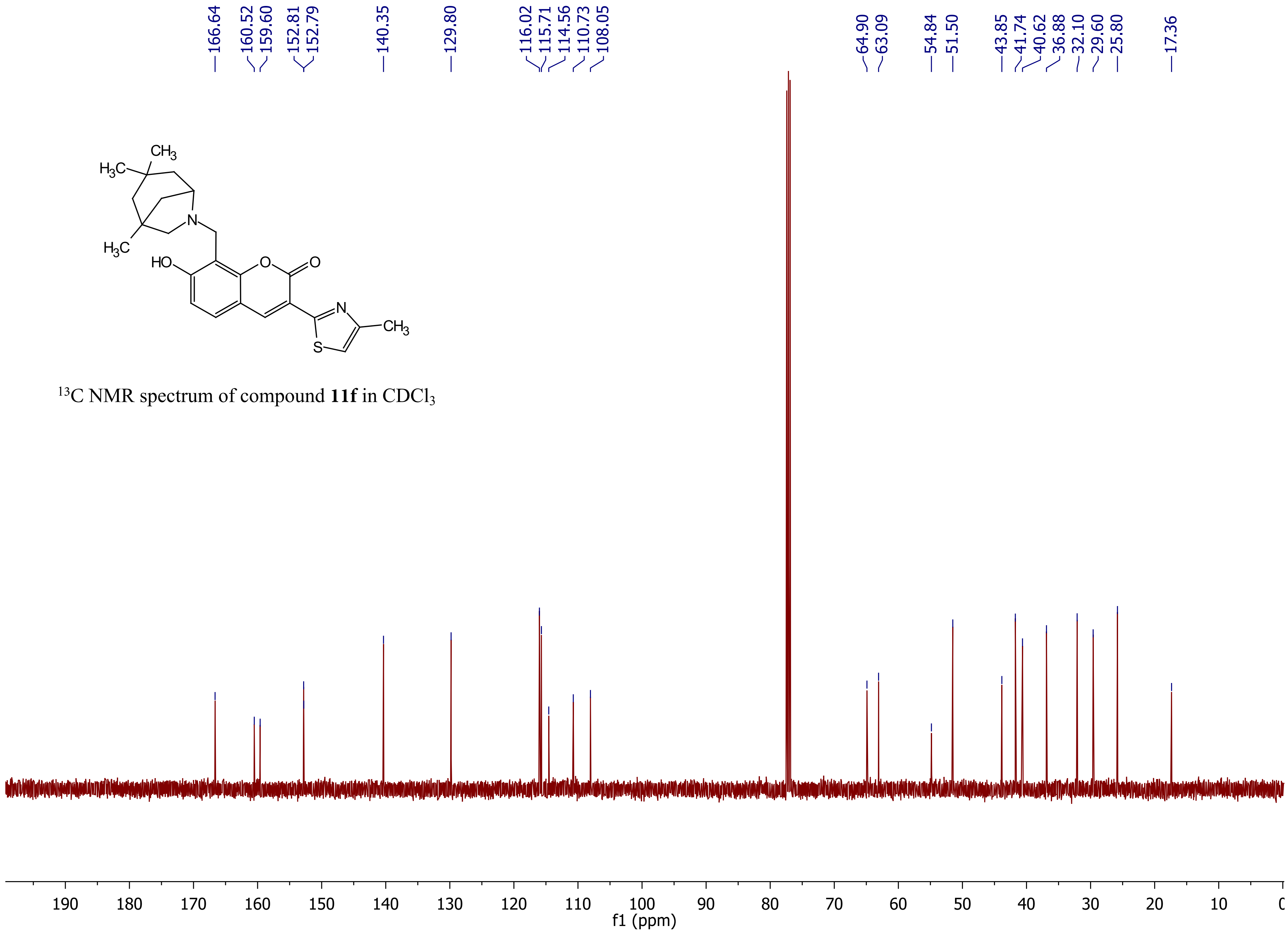

^{13}C NMR spectrum of compound **11f** in $CDCl_3$
